# Causal Effects of Body Mass Index on Airflow Obstruction and Forced Mid-Expiratory Flow: A Mendelian Randomization Study Taking Interactions and Age-Specific Instruments Into Consideration Toward a Life Course Perspective

**DOI:** 10.3389/fpubh.2021.584955

**Published:** 2021-05-11

**Authors:** Nicole Probst-Hensch, Ayoung Jeong, Daiana Stolz, Marco Pons, Paola M. Soccal, Robert Bettschart, Deborah Jarvis, John W. Holloway, Florian Kronenberg, Medea Imboden, Christian Schindler, Gianfranco F. Lovison

**Affiliations:** ^1^Department of Epidemiology and Public Health, Swiss Tropical and Public Health Institute, Basel, Switzerland; ^2^Department of Public Health, University of Basel, Basel, Switzerland; ^3^Clinic of Pulmonary Medicine and Respiratory Cell Research, University Hospital Basel, Basel, Switzerland; ^4^Division of Pulmonary Medicine, Regional Hospital of Lugano, Lugano, Switzerland; ^5^Division of Pulmonary Medicine, Geneva University Hospitals, Geneva, Switzerland; ^6^Lungenpraxis Aarau, Hirslanden Klinik, Aarau, Switzerland; ^7^Medical Research Council-Public Health England, Centre for Environment and Health, Imperial College London, London, United Kingdom; ^8^Population Health and Occupational Disease, National Heart and Lung Institute, Imperial College London, London, United Kingdom; ^9^Human Development and Health, Faculty of Medicine, University of Southampton, Southampton, United Kingdom; ^10^Division of Genetic Epidemiology, Department of Medical Genetics, Molecular and Clinical Pharmacology, Medical University of Innsbruck, Innsbruck, Austria; ^11^Department of Economics, Business and Statistics, University of Palermo, Palermo, Italy

**Keywords:** Mendelian randomization, body mass index, genetic score, lung function, COPD, longitudinal cohort

## Abstract

Obesity has complex links to respiratory health. Mendelian randomization (MR) enables assessment of causality of body mass index (BMI) effects on airflow obstruction and mid-expiratory flow. In the adult SAPALDIA cohort, recruiting 9,651 population-representative samples aged 18–60 years at baseline (female 51%), BMI and the ratio of forced expiratory volume in 1 second (FEV_1_) to forced vital capacity (FVC) as well as forced mid-expiratory flow (FEF25–75%) were measured three times over 20 follow-up years. The causal effects of BMI in childhood and adulthood on FEV1/FVC and FEF25–75% were assessed in predictive (BMI averaged over 1st and 2nd, lung function (LF) averaged over 2nd and 3rd follow-up; *N* = 2,850) and long-term cross-sectional models (BMI and LF averaged over all follow-ups; *N* = 2,728) by Mendelian Randomization analyses with the use of weighted BMI allele score as an instrument variable and two-stage least squares (2SLS) method. Three different BMI allele scores were applied to specifically capture the part of BMI in adulthood that likely reflects tracking of genetically determined BMI in childhood. The main causal effects were derived from models containing BMI (instrumented by BMI genetic score), age, sex, height, and packyears smoked as covariates. BMI interactions were instrumented by the product of the instrument (BMI genetic score) and the relevant concomitant variable. Causal effects of BMI on FEV1/FVC and FEF25–75% were observed in both the predictive and long-term cross-sectional models. The causal BMI- LF effects were negative and attenuated with increasing age, and stronger if instrumented by gene scores associated with childhood BMI. This non-standard MR approach interrogating causal effects of multiplicative interaction suggests that the genetically rooted part of BMI patterns in childhood may be of particular relevance for the level of small airway function and airflow obstruction later in life. The methodological relevance of the results is first to point to the importance of a life course perspective in studies on the etiological role of BMI in respiratory health, and second to point out novel methodological aspects to be considered in future MR studies on the causal effects of obesity related phenotypes.

## Introduction

Obesity, mostly measured as body mass index (BMI) is an established asthma risk factor. Its etiological role with regard to other respiratory phenotypes including chronic obstructive pulmonary disease (COPD) remains unclear (1–4). Observational evidence on the association of obesity with spirometry-derived lung function (LF) is inconclusive (5–12). In adulthood, increasing BMI has been often, but not exclusively, associated with lower forced expiratory volume in 1 second (FEV1) and forced vital capacity (FVC). Bariatric surgery improved FVC and FEV1 in asthmatics over 5 years (13). FEV1/FVC was sometimes preserved or even increased in the presence of excess body weight, but overall the association with airflow obstruction (AO) remains unclear (12). Inconsistencies between studies reflect differences in the study populations (age, health state, ethnicity, lifestyles, environments, socio-economic profile), differences in obesity parameters studied, and statistical models (confounders and effect modifiers considered).

Mechanisms by which obesity in adults can impair LF include increased abdominal pressure due to fat mass, a related decrease in the recoil properties of the chest wall, distal airway closure and lung volume reduction. In addition, excess fat mass may exacerbate systemic and airway inflammation (1, 14–16). In fact, adipose tissue associated immunological and pro-inflammatory factors may already impact on respiratory health during childhood. Weight change patterns in early life were recently associated with dysanapsis in which FVC is higher relative to FEV_1_ as a result of a possible imbalance between alveolar and airway growth (17). Although no study was able to investigate the association of early life weight change patterns with respiratory health in older adults, small airways are known to be frequently involved at a very early stage of COPD and possibly asthma (18).

Small airways are more difficult to study in the absence of a gold standard for measuring their dysfunction. Forced mid-expiratory flow (FEF25–75%, abbreviated as FEF2575 hereafter) is thought to better capture small airways dysfunction than FEV1/FVC (19). It may therefore be more sensitive to reflect chronic effects of obesity on small airways. Few observational BMI–LF studies in adults have considered FEF2575 (20–22). But impulse oscillometry (IOS) studies, more reliable in assessing distal airway function, found increased airway resistance and decreased airway reactance with elevated BMI (15).

Further insight into the causality of the BMI-LF association can be gained by Mendelian randomization (MR) studies (23). Increasingly larger genome-wide association studies (GWAS), primarily in adults, have identified more and more loci associated with BMI at effect sizes and allele frequencies becoming smaller and smaller (24–26), enabling derivation of an instrumental variable. The different GWAS, conducted in adults or in children, allow deriving instrumental variables more specifically targeting either BMI in adulthood or BMI in childhood and thereby reflecting age-related differences in pathways to BMI, an aspect largely ignored in previous studies on BMI and lung function. While the largest BMI GWAS in adults to date (24) (named “Yengo score” in this paper) was not tested for association with childhood BMI, the single nucleotide variants (SNPs) identified in the earlier adult BMI GWAS (named “Speliotes score” in this paper) were explicitly confirmed for association with childhood BMI (26). Yet, the correlation between this latter genetic score with one derived from a recent GWAS meta-analysis on BMI of more than 40 000 children (named “Felix score”) was reported at only 0.73 (25), pointing to differences in genetic pathways determining childhood vs. adulthood BMI.

Only one, large MR meta-analysis has investigated the causal effect of BMI on adult LF and it applied the adult BMI-derived genetic score (“Speliotes score”) (25, 26), but not the childhood BMI-derived genetic score (“Felix score”) (25). This study relied on FEV1, FVC and BMI measured at a single time point in almost 500,000 participants, and supported a causal effect of BMI (2). The causal effect of BMI on other LF parameters relevant to asthma and COPD such as FEV1/FVC, the physiological parameter used to define AO, and FEF2575 (27–30), has not been investigated using an MR approach.

The SAPALDIA cohort with 20 years of BMI and LF follow-up offered the opportunity to study the chronicity of BMI-LF association over an extended period of time in the context of an MR study. We evaluated causal effects (a) of BMI averaged over time points 1 and 2 on lung function averaged over time points 2 and 3 (predictive model) and (b) of BMI and lung function averaged over 3 time points (long-term cross-sectional model). Since BMI fluctuates over time, we instrumented long-term average BMI as a more meaningful exposure measure than BMI from a single time point. Similarly, since lung function fluctuates over time and is measured with error (compliance of participants, field worker effects, spirometry device effects), we focused on long-term average LF as a more meaningful outcome phenotype than level or change in lung function. We applied MR to BMI, even though BMI is only an imprecise measure of adiposity for the following reasons: First, better instrument is available for BMI thanks to large GWAS, compared to other adiposity metrics. Second, SAPALDIA has not longitudinally measured other adiposity metrics as complete as BMI. Third, BMI has been the most common obesity metric associated with lung function in previous studies. The study *a priori* focused on FEV1/FVC and FEF2575 as outcomes and *a priori* instrumented BMI in three different ways (Yengo score, Speliotes score, Felix score) in an attempt to specifically capture the part of BMI in adulthood that reflects the tracking of genetically determined BMI in childhood.

## Methods

### Study Population

SAPALDIA has been described previously (31). Random population samples aged 18–60 years were invited in eight Swiss study areas for the baseline survey in 1991 (SAP1). Of the 9,651 baseline participants, 8,047 (83.4%) participated in follow-up SAP2 (2001/3) and 6,139 (63.6%) in follow-up SAP3 (2010/11). This paper was restricted to participants in all three surveys with complete spirometry, BMI, genotype and covariate data for the respective causal model (Supplementary Figures 1A,B).

Ethical approval was obtained for each survey and study area from the central ethics committee of the Swiss Academy of Medical Sciences and the Cantonal Ethics Committees. Participants provided informed consent. All methods were performed in accordance with the relevant guidelines and regulations.

### Lung Function

Spirometry was conducted with heated-wire spirometers (SensorMedics, Yorba Linda, California) (SAP1 & SAP2), and by portable, ultrasonic EasyOne spirometer (ndd medizintechnick AG, Zürich, Switzerland) (SAP3), according to American Thoracic Society recommendations (32) (see Supplementary Material). The LF parameters considered for this study are the ratio FEV1/FVC, forced mid-expiratory flow FEF2575, and FEF2575/FVC (results in Supplementary Material), derived from pre-bronchodilation spirometry. FEV1 and FVC decline as airway narrows. A reduced FEV1/FVC defines AO, resulting if the decline in FEV1 is out of proportion to the decline in FVC, while reduced FVC indicates restriction. FEF2575 is an early indicator of AO and sensitive to small airway dysfunction. Reduced FEF2575/FVC is an indicator of dysanapsis where lung volume increases as a result of air trapping in the presence of AO. SAP3 measurements were re-calibrated to assure comparability with SAP1 and SAP2 measurements (33).

### BMI and Covariates

Height was measured. Weight was asked for at baseline, but measured at follow-up. BMI was calculated in kg/m^2^. Exact age was calculated based on birth and examination dates. Sex was self-reported. Smoking was self-reported and measured as pack-years smoked up to baseline and during the two follow-up periods. Non-asthmatics where defined as those who never reported a doctor diagnosis of asthma.

### Genotyping

DNA was extracted from EDTA blood. 570k SNPs were genotyped for 1,612 SAPALDIA samples by Human610-Quad BeadChip (Illumina, San Diego, CA, USA) (34) and ~1 million SNPs were genotyped for additional 3,015 SAPALDIA samples by Infinium Human OmniExpressExome-8 (Illumina, San Diego, CA, USA) (35). Samples with call rate <0.97 or population outliers were excluded. Markers with call rate <0.95, minor allele frequency <0.05, or out of Hardy-Weinberg equilibrium (*p* <10^−6^) were excluded. The genotype datasets were then phased using ShapeIT (v2.r790) (36) and imputed using MiniMac2 (version 2014) (37) to 1,000 Genome phase 1 reference panel comprising of 1,092 samples. The imputed datasets were merged to yield 38 million markers.

### BMI Allele Score

The genetic instruments for BMI were single-nucleotide polymorphisms (SNPs) independently [linkage disequilibrium (LD) *R*^2^ measure < 0.2] associated in Caucasians with the BMI at a genome-wide level (*P* < 5 × 10^−8^). Three scores were derived, i.e., “Speliotes Score” (adult BMI GWAS also associated with childhood BMI, used in the only previous BMI-LF MR study); “Felix Score” (childhood BMI GWAS); and “Yengo Score” (largest adult BMI GWAS, unknown association with childhood BMI). They were computed as weighted sum of 32, 12, and 862 BMI-increasing alleles reported by Speliotes et al. (26), Felix et al. (25), and Yengo et al. (24), respectively, using the reported coefficients for each SNP as weights, following the same approach as earlier MR studies of BMI (2, 38). We excluded SNPs with poor imputation quality (*r*^2^ < 0.3) or with known association with smoking phenotypes in PhenoScanner. rs13387838 for which visual inspection of MR Egger regression results clearly indicated pleiotropy was further excluded from Felix Score. Supplementary Table 1 describes the 32, 12, and 862 SNPs used to construct Speliotes et al. (26), Felix et al. (25), and Yengo et al. (24) scores, respectively.

As the weights' sum is bounded by the number of SNPs considered, the effect size of each score can be interpreted as average effect per one BMI-increasing allele. The three scores were only moderately correlated (0.28–0.55). The correlation was smallest between the Yengo and the Felix scores (0.28) (Supplementary Table 2).

### Statistical Analysis

Statistical analyses were performed in R, version 3.4.3 for Windows (http://www.r-project.org/) (see the “Statistical analysis” section in the Supplementary Material).

#### Analysis Scheme

We investigated the causal association in a *predictive model* (exposure: BMI averaged over SAP1 and SAP2, referred to as SAP1-SAP2; outcome: LF averaged over SAP2 and SAP3, referred to as SAP2-SAP3) and in a *long-term cross-sectional model* (BMI and LF both averaged over SAP1, SAP2, and SAP3; referred to as SAP1-SAP2-SAP3).

#### Descriptive Analysis

Characteristics of study participants were summarized for the combinations of surveys involved in the modeling phase: SAP1-SAP2, SAP2-SAP3, and SAP1-SAP2-SAP3. Partial correlation coefficients were computed: (i) between the same LF variables over the 3 occasions in time to assess temporal auto-correlation; and (ii) between the different LF variables (and their derived averages) at each occasion in time to assess their degree of (linear) relationship. Partial correlations were computed using residuals of each LF variable from models that regress them on Age, Age^2^, Height, Height^2^, Sex, and all their first-order interactions. Pairwise complete cases analysis was performed, to accommodate the differential presence of missing values in the variables involved. BMI distribution at each survey was visualized as histograms.

#### Checking MR Assumptions

In preparing for MR analysis, a set of assumptions as highlighted in VanderWeele et al. (39) were checked:

(1) The genetic score is associated with the exposure. In the context of our study, this requires testing the presence of an association of BMI genetic score with BMI;
(2) The genetic score is not associated with confounders of the exposure–outcome relationship. In the context of our study, this required various actions: (2.1) testing that the BMI genetic score is not associated with the observed confounders Packyears and Height; (2.2) SNPs identified in PhenoScanner (40) as associated with smoking phenotypes, were excluded from computing genetic scores; (2.3) MR-Egger regression (41) was conducted to check for pleiotropy; (2.4) We interrogated whether age or sex modify the influence of BMI genetic score on phenotypic BMI by regressing the BMI averages on a linear predictor including Age (averaged over SAP1-SAP2 and SAP1-SAP2-SAP3, respectively, and centered at 18 years), Sex, BMI genetic score and all their interactions;
(3) The genetic score is not associated with the outcome, conditional on the exposure and confounders of the exposure–outcome relationship. In the context of our study, this requires testing the absence of a BMI genetic score association with LF, conditional on BMI and (observed) confounders of the BMI-LF relationship.

In all these checks, the models used were chosen through a selection procedure carried out within the class of (extended) Generalized Linear Models, with the aim of making the choice more flexible and finding the model most appropriate in terms of both distribution of response and possible non-linearity of the relationship of the response with the predictors (see Supplementary Material for details).

#### Mendelian Randomization Analysis

As MR assumptions appeared to be satisfied in our data, instrumental variable (IV) analyses were carried out to test and estimate the causal effects of BMI on LF in the context of Linear Gaussian models (42). Estimation was carried out using the two-Stage Least Squares (2SLS) method. In the first-stage of 2SLS, the exposure is regressed on the genetic score to give fitted values of the exposure (“Exposure models”). In the second-stage, the outcome is regressed on the fitted values for the exposure from the first stage regression, along with other covariates (“Causal model”). The causal estimate is this second-stage regression coefficient for the change in outcome caused by a unit change in the exposure. Details can be found in Burgess and Thompson (43) (ch. 4.2). All MR analyses were carried out using the ivreg command of the R library AER.

The first- and second-stage analyses were based on identical data. The response variables were LF parameters averaged over either SAP2-SAP3 (predictive model) or SAP1-SAP2-SAP3 (long-term cross-sectional model). The causal variable (instrumented by the respective BMI genetic score) was the logarithm of the BMI averages over either SAP1-SAP2 (predictive model) or SAP1-SAP2-SAP3 (long-term cross-sectional model). The choice of log-transforming the BMI averages was made through an AIC-based selection procedure. This transformation appeared to be the best linear predictor for all LF outcomes and the best choice in checking MR Assumption 1 (see the Results for details).

Explanatory variables for each LF variable were chosen through a model selection procedure. The initial (maximal), and *a priori* sparse, model contained the following covariates: (instrumented) BMI, Age (centered at 18 years, the minimal admission age at SAP1), Sex, Height, and Packyears smoked, along with all their pairwise interactions. Physical activity was *a priori* not included in the model due to its potential role as mediator of the BMI-LF association. We decided not to include study center and educational level after we observed adding them to the final causal and observational models did not materially alter the effect estimates.

It is to be stressed that the inclusion of interactions implies that all the interaction parameters between BMI and all other variables must also be considered as causal, and must be themselves instrumented; this represents an innovative aspect of this paper, since models used in MR studies are usually assumed to be additive, and no attempt is made to check the appropriateness of this assumption. In a non-standard MR approach and following a suggestion by Bun and Harrison (44), the interrogation of causal interactions was instrumented by the product of the instrument (BMI genetic score) and the relevant concomitant variable. Given that age can be neither genetically determined nor confounded, BMI:Age interaction is a special case and our approach cannot be generalized into other interaction MR analyses.

Starting from the maximal model, a model selection procedure, based on AIC comparisons, provided the final model which retained (instrumented) BMI and its interaction with Age, as well as Age, Sex, Height, Packyears smoked, Age × Sex and Age × Height interactions. Standard errors for the causal parameter IV estimates were obtained by second order delta method. Wald confidence intervals were derived based on asymptotic Normality. In all models, the error distribution was assumed to be Normal, so that in all exposure models the response on the original scale (BMI_s1,s2_ and BMI_s1,s2,s3_) was assumed to be logNormal (see Supplementary Material for details).

The same final models were selected for the Mendelian Randomization analyses on the two lung function variables of main interest in this paper (FEV1/FVC and FEF2575). Their IV representation is as follows.

#### Predictive Model

(1) $$\begin{array}{l} \text{Causal model}:\text{E}[{\text{LF}}_{\text{s2},\text{s3}}]={\text{β}}_{0}\text{+ }{\text{β}}_{\text{c1}}\text{log}({\text{BMI}}_{\text{s1},\text{s2}})\\ \text{ +}{\text{β}}_{1}\text{Age}\_{\text{c}}_{\text{s1},\text{s2}}{\text{ + β}}_{2}{\text{Sex}}_{\text{s2}}\\ \text{ +}{\text{β}}_{3}{\text{Height}}_{\text{s1},\text{s2}}{\text{ + β}}_{4}{\text{PackYrs}}_{\text{s2}}\\ \text{ +}{\text{β}}_{\text{c2}}\text{log}({\text{BMI}}_{\text{s1},\text{s2}})\text{×Age}\_{\text{c}}_{\text{s1s2}}\\ \text{ +}{\text{β}}_{\text{5}}\text{Age}\_{\text{c}}_{\text{s1},\text{s2}}\text{×}{\text{Sex}}_{\text{s2}}\text{+}{\text{β}}_{6}\text{Age}\_{\text{c}}_{\text{s1},\text{s2}}\\ \text{ ×}{\text{Height}}_{\text{s1},\text{s2}} \end{array}$$

(2) $$\begin{array}{l} \text{ Exposure models}:\text{ E}[\text{log}({\text{BMI}}_{\text{s1},\text{s2}})]={\text{α}}_{0}+{\text{α}}_{1}{\text{BMI}}_{\text{gs}} \end{array}$$

(3) $$\begin{array}{l} \text{ E}[\text{log}({\text{BMI}}_{\text{s1},\text{s2}}):\text{Age}\_{\text{c}}_{\text{s1},\text{s2}}]\\ \;{\text{= γ}}_{0}\text{+ }{\text{γ}}_{1}{\text{BMI}}_{\text{gs}}\times \text{Age}\_{\text{c}}_{\text{s1},\text{s2}} \end{array}$$

#### Long-Term Cross-Sectional Model

(4) $$\begin{array}{l} \text{Causal model}:\text{E}[{\text{LF}}_{\text{s1},\text{s2},\text{s3}}]={\text{β}}_{0}\text{+ }{\text{β}}_{\text{c1}}\text{log}({\text{BMI}}_{\text{s1},\text{s2},\text{s3}})\\ \;{\text{+β}}_{1}\text{Age}\_{\text{c}}_{\text{s1},\text{s2},\text{s3}}\text{+ }{\text{β}}_{2}{\text{Sex}}_{\text{s2}}\\ \;{\text{+β}}_{3}{\text{Height}}_{\text{s1},\text{s2},\text{s3}}\text{+ }{\text{β}}_{4}{\text{PackYrs}}_{\text{s3}}\;\\ \;{\text{+β}}_{2}\text{log}({\text{BMI}}_{\text{s1},\text{s2},\text{s3}})\text{×Age}\_{\text{c}}_{\text{s1},\text{s2},\text{s3}}\\ \;{\text{+β}}_{5}\text{log}({\text{BMI}}_{\text{s1},\text{s2},\text{s3}})\text{×}{\text{Sex}}_{\text{s2}}\\ \;{\text{+β}}_{6}\text{Age}\_{\text{c}}_{\text{s1},\text{s2},\text{s3}}\text{×}{\text{Height}}_{\text{s1},\text{s2},\text{s3}} \end{array}$$

(5) $$\text{ Exposure models}:\text{ E}[\text{log}({\text{BMI}}_{\text{s1},\text{s2},\text{s3}})]\text{ = }{\text{α}}_{0}$$

(6) $$\begin{array}{l} {\text{ +α}}_{1}{\text{BMI}}_{\text{gs}}\\ \text{ E}[\text{log}({\text{BMI}}_{\text{s1},\text{s2},\text{s3}}):\text{Age}\_{\text{c}}_{\text{s1},\text{s2},\text{s3}}]\\ \text{ = }{\text{γ}}_{0}\text{+ }{\text{γ}}_{1}{\text{BMI}}_{\text{gs}}\times \text{Age}\_{\text{c}}_{\text{s1},\text{s2},\text{s3}} \end{array}$$

where: LF (Lung Function) is either FEV1/FVC or FEF2575;

β_c1_ and β_c2_ are the causal effect parameters;
all variables with multiple subscripts are averages over the relevant SAPALDIA surveys (e.g., BMI_s1,s2_ is the average of BMI_s1_ and BMI_s2_);
Age_c is Age averaged over either SAP1-SAP2 or SAP1-SAP2-SAP3 and centered at 18 years;
PackYrs_si_ = Pack-years smoked up to SAP_i_ (i = 2 or 3)
BMI_gs_ is the BMI genetic score (either Speliotes, Felix, or Yengo score).

#### Observational Association Analysis

The BMI-LF associations were analyzed using linear regression analyses adjusted for Sex, Age, Height, and Packyears smoked. For comparability with the MR results, the same final models [(1) and (4)] were re-fitted, using observed BMI (and observed Age × BMI interaction) instead of instrumenting them, and estimated by Ordinary Least Squares.

#### Sensitivity Analysis

The reliability of self-reported, instead of measured, weight at SAP1 was assessed by comparing the estimated regression coefficient and the estimated determination coefficient R^2^ of the BMI_s1_ vs. BMI_s2_ and BMI_s3_ relationships with BMI genetic scores. In order to check the possible effects due to the non-Normality of the LF variables we re-fitted the final models (1–6) employed in MR analysis using log-transformed (FEF2575) and logit-transformed (FEV1/FVC) parameters as outcomes. MR analysis was repeated using the ratio FEF2575/FVC as outcome (20) and for non-asthmatics, again re-fitting the final models (1–6).

In a preliminary analysis, we also investigated the association between *changes* in BMI and *changes* in LF, to check if this was a better way of exploiting the longitudinal nature of our data, compared to the use of medium- and long-term averages. Notice that we could perform this analysis only in observational association terms, since no genetic variants for BMI change are available.

The attrition bias due to potentially disproportionate lost to follow up over 20 years was interrogated by replicating the observational association analysis using Inverse Probability Weighted analysis, where the weights were either (1) the probability of participation in SAP2 and SAP3 given the variables used in the models (BMI, LF (either FEF2575 or FEV1/FVC), Age, Sex, Height, Packyears) measured at SAP1; or (2) the probability of participation in SAP3 given the variables used in the models (BMI, LF (either FEF2575 or FEV1/FVC), Age, Sex, Height, Packyears) averaged over SAP1-SAP2.

As a *post-hoc* analysis, we conducted stratified analysis by fitting the same final models, except for the Age × BMI interaction, in the strata defined based on tertiles of age at SAP1, using Speliotes score as instrument.

## Results

### Descriptive Analysis

**Figure 1:**
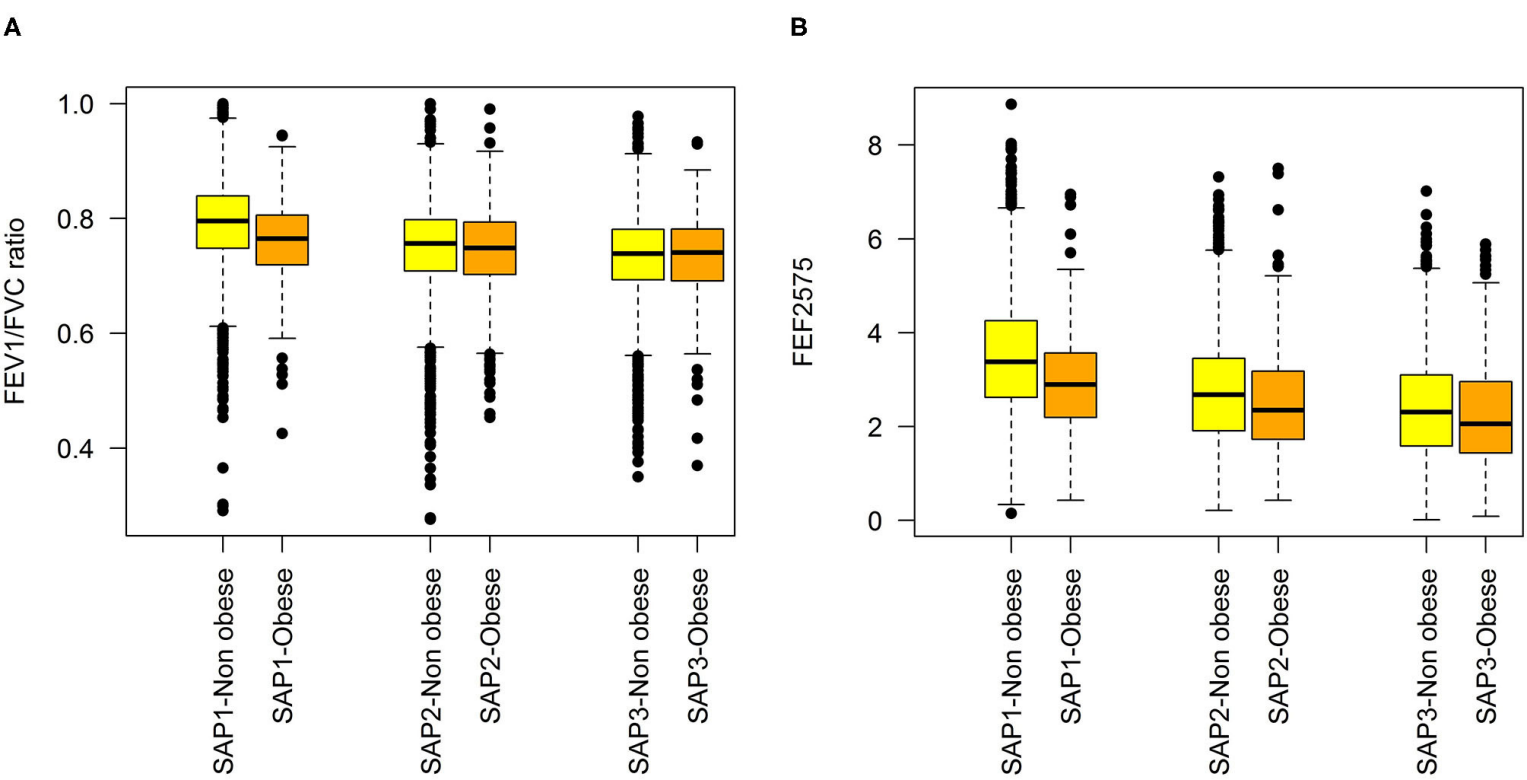
Distributions of lung function variables at each SAPALDIA survey, by obesity state (BMI < 30 kg/m^2^ vs. ≥30 kg/m^2^: **(A)** FEV1/FVC, **(B)** FEF2575.

Characteristics of the study samples used for fitting the predictive and the long-term cross-section model are presented in Table 1. Variability of the LF variables, both within and between SAPALDIA surveys, and stratified by obesity is graphically depicted in Figures 1A,B. LF was lower among obese persons, but the difference became weaker (FEF2575) or disappeared (FEV1/FVC), as participants aged. Inverse associations not dependent on age were observed for FEV1 and FVC (Supplementary Figure 2). Partial correlations between the LF parameters, and the derived means, are presented in Supplementary Table 3. Histograms of BMI at each survey are presented in Supplementary Figure 3.

**Table 1:** Characteristics of study participants included in the sample: **(A)** used to fit the predictive model; **(B)** used to fit the long-term cross-sectional model.

**(A) Sample of the predictive model**		
	**SAP1, SAP2**	**SAP2, SAP3**
	***N*** **=** **2,850**	
Sex at s2, % female	49.35	
Mean (s1, s2) Age, years (mean; SD)	44.71 (10.81)	
Mean (s1, s2) Height, cm (mean; SD)	170.11 (8.85)	
Mean (s1, s2) Weight, kg^a^ (mean; SD)	71.64 (13.25)	
Mean (s1, s2) BMI, kg/m^2^ (mean; SD)	24.44 (3.54)	
Packyears of cigarettes at s2 (mean; SD)	10.47 (17.13)	
Mean (s2, s3) FEF2575, ml^b^ (mean; SD)		2.58 (1.08) (*N* = 2,936)
Mean (s2, s3) FEV1/FVC^2^ (mean; SD)		0.74 (0.07) (*N* = 2,939)
Asthma up to s2 (% doctor diagnosed asthma)	10.30	
**(B) Sample of the long-term model**		
	**SAP1, SAP2, SAP3**	
	***N*** **=** **2,728**	
Sex at s2, % female	50.53	
Mean (s1, s2, s3) Age, years (mean; SD)	49.43 (10.78)	
Mean (s1, s2, s3) Height, cm (mean; SD)	169.63 (8.87)	
Mean (s1, s2, s3) Weight, kg^a^ (mean; SD)	72.02 (13.21)	
Mean (s1, s2, s3) BMI, kg/m^2^ (mean; SD)	24.95 (3.68)	
Packyears of cigarettes at s3 (mean; SD)	11.48 (18.74)	
Mean (s1, s2, s3) FEF2575, ml^b^ (mean; SD)	2.89 (1.07)	
Mean (s1, s2, s3) FEV1/FVC^2^ (mean; SD)	0.76 (0.06)	
Asthma (% doctor diagnosed asthma ever)	13.35	

a *Weight was self-reported at baseline, and measured at follow-up*.

b *Lung function at SAP3 was corrected for change in spirometry device (33)*.

### Checking the MR Assumptions

MR assumptions appeared to be satisfied (for details see Supplementary Material and Supplementary Tables 4A,B). The three BMI genetic scores derived from different life-course specific BMI variants were predictors of adult BMI, with the Yengo score being the strongest instrument (F-statistics 182 and 233 for long-term cross-sectional and predictive models, respectively). They were not associated with Packyears or Height. None of the SNPs included in the BMI genetic scores overlap with 154 smoking-related SNPs (45–47). One of the BMI SNPs (rs10767664) was in high LD with several smoking initiation associated SNPs in BDNF (brain derived neurotrophic factor) (*R*^2^ = 0.681 ~ 0.911), but was not associated with smoking initiation in SAPALDIA, irrespective of adjustment for Age, Sex, and BMI. None, two, and sixteen SNPs were excluded from Speliotes, Felix, and Yengo Score, respectively, due to known association with smoking phenotypes in PhenoScanner. MR Egger regression did not indicate potential pleiotropy for main BMI effects. Slight indication of pleiotropy for the Age × BMI interaction was observed in FEV1/FVC and FEF2575 prediction models for Speliotes Score and in FEF2575 cross-sectional model for Felix Scores (see Supplementary Table 5, Supplementary Figures 4–6). We did not observe Age, Sex, or their combination to modify the association of BMI genetic score with phenotypic BMI (data not shown).

### Mendelian Randomization Analysis

**Figure 2:**
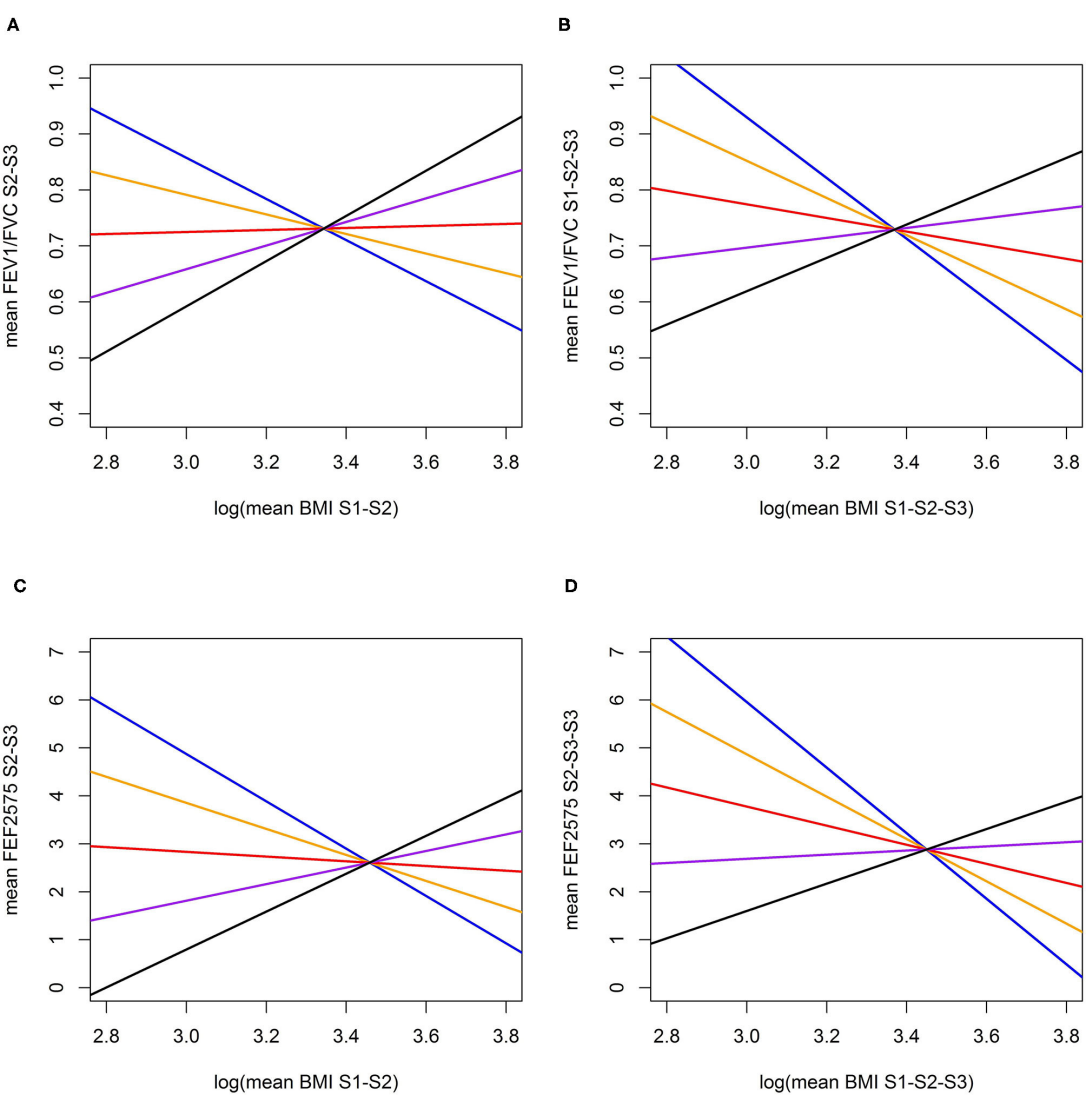
Predictive and long-term cross-sectional total causal (26) effect of BMI on FEV1/FVC **(A,B)** and FEF2575 **(C,D)** for a reference individual (Male, Height = 175 cm., Never Smoker) at specific ages (Blue: at age 28, Orange: at age 38, Red: at age 48; Purple: at age 58; Black: at age 68). **(A)** Total predictive effect of BMI (log mean over SAP1-SAP2) on FEV1/FVC ratio (mean over SAP2-SAP3); **(B)** Total long-term cross-sectional effect of BMI (log mean over SAP1-SAP2-SAP3) on FEV1/FVC ratio (mean over SAP1-SAP2-SAP3); **(C)** Total predictive effect of BMI (log mean over SAP1-SAP2) on FEF2575 (mean over SAP1-SAP2); **(D)** Total long-term cross-sectional effect of BMI (log mean over SAP1-SAP2-SAP3) on FEF2575 (mean over SAP1-SAP2-SAP3).

Causal effects of BMI on FEV1/FVC and FEF2575 were observed in the predictive and long-term cross-sectional models (Tables 2–4 for Speliotes, Felix and Yengo Scores, respectively). For the Speliotes Score the causal effect of BMI on these two LF parameters was negative, but attenuated with increasing Age. Figure 2 illustrates the age-BMI interaction with a 175 cm tall male never smoker as a reference individual. If he is 18 years old at SAP1, and hence his average age is 28 over the 20 years period between SAP1 and SAP3, and during this period his BMI changes from 25 to 30, employing the estimates in Table 2 we can predict he will experience on average a decrease in his FEV1/FVC ratio approximately equal to 0.10. On the other hand, if he is 48 years old at SAP1, and hence his average age is 58 over the 20 years period between SAP1 and SAP3, the same change of BMI from 25 to 30 will cause an increase in his FEV1/FVC ratio ≈0.016. Causal effects were in the same direction, but with confidence intervals covering no effect, for the Yengo and the Felix Scores. Effect estimates of the Felix Score were about as large as for the Speliotes Score, whereas effect estimates for the Yengo score were considerably smaller. Irrespective of the genetic score, no BMI interactions with covariates other than Age were present. No causal effect of BMI on FEV1 or FVC was observed (results not presented).

**Table 2:** Causal effects^a^ of BMI on FEV1/FVC and FEF2575 in predictive and in long-term cross-sectional models.

	***N***	****β_c1,_β_c2_****	**SE**	***p*-value**
**FEV1/FVC**				
**Predictive model**				
BMI main effect log(BMI_s1s2_) → FEV1/FVC_s2,s3_	2,853	−0.561	0.256	0.029
BMI^*^Age interaction effect log(BMI_s1s2_):Age_s1s2_ → FEV1/FVC_s2,s3_		0.019	0.010	0.065
**Long-term cross-sectional model**				
BMI main effect log(BMI_s1s2s3_) → FEV1/FVC_s1,s2,s3_	2,731	−0.752	0.314	0.017
BMI*Age interaction effect log(BMI_s1s2s3_):Age_s1,s2,s3_ → FEV1/FVC_s1,s2,s3_		0.021	0.010	0.040
**FEF2575**				
**Predictive model**				
BMI main effect log(BMI_s1s2_) → FEF2575_s2,s3_	2,850	−7.152	3.457	0.038
BMI*Age interaction effect log(BMI_s1s2_):Age_s1s2_ → FEF2575_s2,s3_		0.222	0.141	0.116
**Long-term cross-sectional model**				
BMI main effect log(BMI_s1,s2,s3_) → FEF2575_s1,s2,s3_	2,728	−9.251	4.433	0.037
BMI*Age interaction effect log(BMI_s1,s2,s3_):Age_s1,s2,s3_ → FEF2575_s1,s2,s3_		0.242	0.146	0.096

*BMI genetic score: (Speliotes; 32 SNPs)*.

a $\beta_{\text{c1}}$, causal BMI main effect per one BMI-increasing allele; β_c2_, causal BMI*Age interaction effect per one BMI-increasing allele.

*The negative sign of the causal main effect means that, keeping all other predictors fixed, at age 18 (which has been chosen as the origin in our analysis) BMI has a causal negative effect on LF. The positive sign of the Age × BMI causal interactive effect implies that, as age increases, the detrimental effect of BMI on LF decreases. As a consequence, the total effect of BMI becomes null at middle ages and protective at older ages; for a graphical representation of such BMI total effects by selected ages see Figure 2*.

**Table 3:** Causal effects^a^ of BMI on FEV1/FVC and FEF2575 in predictive and in long-term cross-sectional models.

	***N***	****β_c1,_β_c2_****	**SE**	***p*-value**
**FEV1/FVC**				
**Predictive model**				
BMI main effect log(BMI_s1s2_) → FEV1/FVC_s2,s3_	2,853	−0.468	0.455	0.300
BMI^*^Age interaction effect log(BMI_s1s2_):Age_s1s2_ → FEV1/FVC_s2,s3_		0.011	0.015	0.490
**Long-term cross-sectional model**				
BMI main effect log(BMI_s1s2s3_) → FEV1/FVC_s1,s2,s3_	2,731	−0.479	0.565	0.400
BMI*Age interaction effect log(BMI_s1s2s3_):Age_s1,s2,s3_ → FEV1/FVC_s1,s2,s3_		0.006	0.016	0.730
**FEF2575**				
**Predictive model**				
BMI main effect log(BMI_s1s2_) → FEF2575_s2,s3_	2,850	−9.932	6.622	0.134
BMI^*^Age interaction effect log(BMI_s1s2_):Age_s1s2_ → FEF2575_s2,s3_		0.269	0.225	0.231
**Long-term cross-sectional model**				
BMI main effect log(BMI_s,1s2,s3_) → FEF2575_s1,s2,s3_	2,728	−9.111	8.117	0.262
BMI^*^Age interaction effect log(BMI_s1,s2,s3_):Age_s1,s2,s3_ → FEF2575_s1,s2,s3_		0.163	0.234	0.486

*Childhood BMI genetic score: (Felix; 12 SNPs)*.

a $\beta_{c1}$, causal BMI main effect per one BMI-increasing allele; β_c2_, causal BMI^*^Age interaction effect per one BMI-increasing allele.

*The negative sign of the causal main effect means that, keeping all other predictors fixed, at age 18 (which has been chosen as the origin in our analysis) BMI has a causal negative effect on LF. The positive sign of the Age × BMI causal interactive effect implies that, as age increases, the detrimental effect of BMI on LF decreases. As a consequence, the total effect of BMI becomes null at middle ages and protective at older ages*.

**Table 4:** Causal effects^a^ of BMI on FEV1/FVC and FEF2575 in predictive and in long-term cross-sectional models.

	***N***	****β_c1,_β_c2_****	**SE**	***p*-value**
**FEV1/FVC**				
**Predictive model**				
BMI main effect log(BMI_s1s2_) → FEV1/FVC_s2,s3_	2,853	−0.140	0.117	0.233
BMI^*^Age interaction effect log(BMI_s1s2_):Age_s1s2_ → FEV1/FVC_s2,s3_		0.005	0.004	0.255
**Long-term cross-sectional model**				
BMI main effect log(BMI_s1s2s3_) → FEV1/FVC_s1,s2,s3_	2,731	−0.226	0.132	0.088
BMI*Age interaction effect log(BMI_s1s2s3_):Age_s1,s2,s3_ → FEV1/FVC_s1, s2, s3_		0.005	0.003	0.173
**FEF2575**				
**Predictive model**				
BMI main effect log(BMI_s1s2_) → FEF2575_s2,s3_	2,850	−2.715	1.594	0.089
BMI*Age interaction effect log(BMI_s1s2_):Age_s1s2_ → FEF2575_s2,s3_		0.087	0.056	0.118
**Long-term cross-sectional model**				
BMI main effect log(BMI_s1,s2,s3_) → FEF2575_s1,s2,s3_	2,728	−3.073	1.885	0.103
BMI^*^Age interaction effect log(BMI_s1,s2,s3_):Age_s1,s2,s3_ → FEF2575_s1,s2,s3_		0.076	0.056	0.175

*BMI genetic score: (Yengo; 862 SNPs)*.

a $\beta_{\text{c1}}$, causal BMI main effect per one BMI-increasing allele; β_c2_, causal BMI^*^Age interaction effect per one BMI-increasing allele.

*The negative sign of the causal main effect means that, keeping all other predictors fixed, at age 18 (which has been chosen as the origin in our analysis) BMI has a causal negative effect on LF. The positive sign of the Age × BMI causal interactive effect implies that, as age increases, the detrimental effect of BMI on LF decreases. As a consequence, the total effect of BMI becomes null at middle ages and protective at older ages*.

### Observational Association Analysis

The Ordinary Least Squares estimates of the observational associations of BMI with FEF2575 and FEV1/FVC, in terms of both main effects and interactions with Age, are presented in Table 5. Associations of BMI with FEV1/FVC and FEF2575 were in opposite directions: negative main effects and positive interactions with Age for FEV1/FVC, positive main effects and negative interactions with Age for FEF2575.

**Table 5:** Observational associations of BMI with FEV1/FVC and FEF2575 in predictive and in long-term cross-sectional models.

	***N***	****β_1,_**$\beta_{\text{c1}},\;\beta_{\text{c2}}$**	**SE**	***p*-value**
**FEV1/FVC**				
**Predictive model**				
BMI main effect log(BMI_s1s2_) → FEV1/FVC_s2,s3_	2,853	−0.006	0.025	0.803
BMI^*^Age interaction effect log(BMI_s1s2_):Age_s1s2_ → FEV1/FVC_s2,s3_		0.001	0.001	0.378
**Long-term cross-sectional model**				
BMI main effect log(BMI_s1s2s3_) → FEV1/FVC_s1,s2,s3_	2,731	−0.047	0.026	0.076
BMI*Age interaction effect log(BMI_s1s2s3_):Age_s1, s2,s3_ → FEV1/FVC_s1, s2, s3_		0.001	0.001	0.179
**FEF2575**				
**Predictive model**				
BMI main effect log(BMI_s1s2_) → FEF2575_s2,s3_		0.746	0.330	0.024
BMI*Age interaction effect log(BMI_s1s2_):Age_s1s2_ → FEF2575_s2,s3_	2,850	−0.019	0.011	0.085
**Long-term cross-sectional model**				
BMI main effect log(BMI_s1,s2,s3_) → FEF2575_s1,s2,s3_		0.525	0.375	0.162
BMI^*^Age interaction effect log(BMI_s1,s2,s3_):Age_s1,s2,s3_ → FEF2575_s1, s2, s3_	2,728	−0.017	0.011	0.114

a $\beta_{\text{c1}}$, associational BMI main effect; β_2_, associational BMI^*^Age interaction effect.

**Figure 3:**
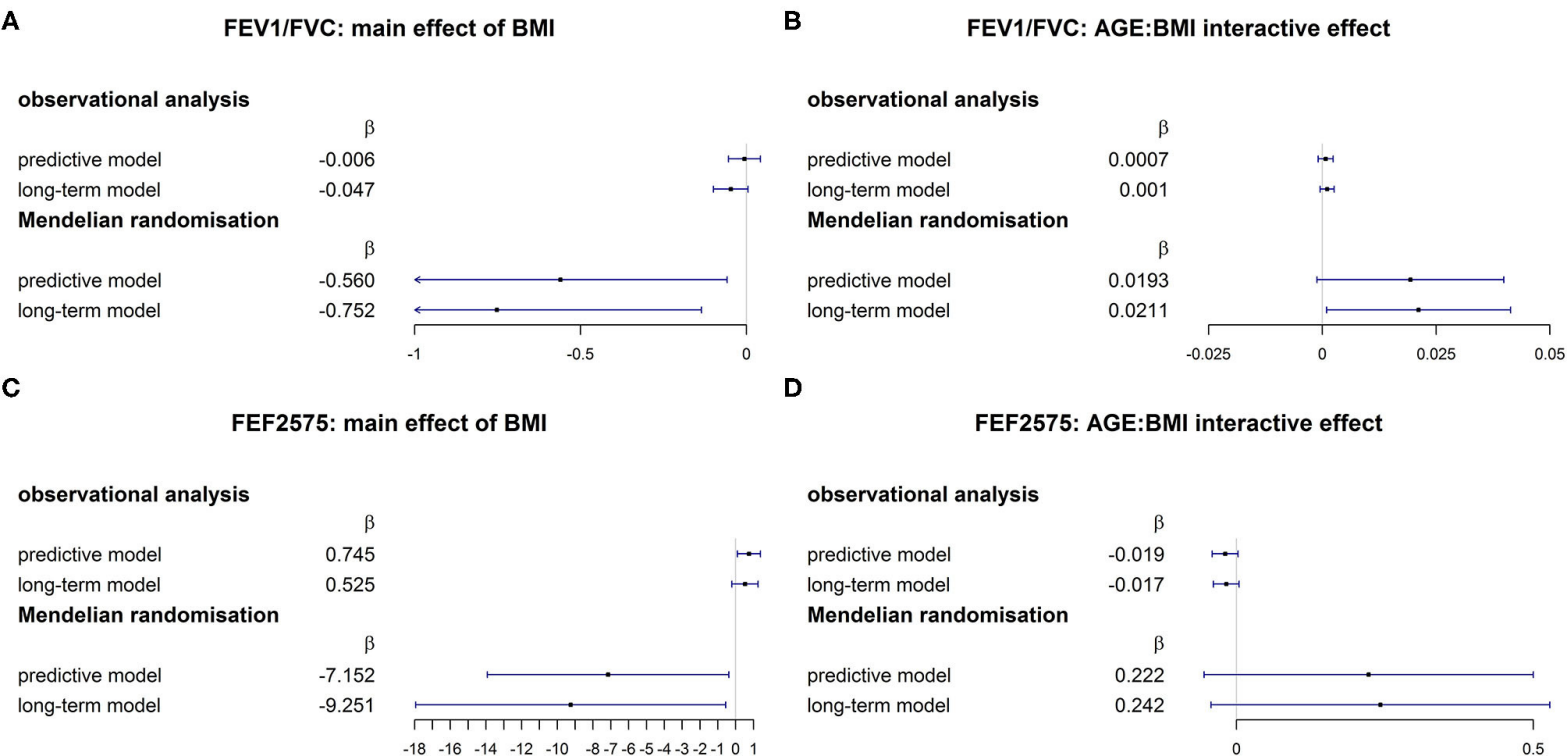
Comparison of associational and MR causal (26) effects for FEV1/FVC [**(A)**: main effect of BMI; **(B)** AGE × BMI interaction] and FEF2575 [**(C)** main effect of BMI; **(D)** AGE × BMI interaction].

The comparison of MR causal effects and observational associations is visually helped by the forest plots in Figures 3A–D. While directions of MR causal effects and observational associations were consistent for FEV1/FVC, they were opposite for FEF2575. Confidence intervals were considerably wider for causal effects compared to observational associations.

To further investigate possible sources of the considerable discrepancy between causal and observational BMI effects, we tried to check whether this could be due to the “composite” nature of BMI as a measure of obesity [for a related discussion of the difficulty of conducting causal inference with composite exposures, see (48)]. To this goal (see more detailed explanation in the Supplementary Material), we refitted the second stage IV models that contained both BMI_instrumented_ and BMI_residual_ = BMI-BMI_instrumented_, derived from the IV first stage. BMI_residual_, which reflects the non-genetically determined BMI variability, explained over 90% of observed BMI variability. We confirmed the positive association of BMI_residual_ on FEF2575 and the lack of its association with FEV1/FVC, consistent with our observational analysis (see results in Supplementary Table 6).

### Sensitivity Analysis

The comparison of regression estimates for BMI_s1_, BMI_s2_, and BMI_s3_ on the three BMI genetic scores confirmed the reliability of BMI_s1_ derived from self-reported weight (Supplementary Table 7). Irrespective of genetic score, the regression results for log-transformed (for FEF2575) and logit-transformed (for FEV1/FVC) outcomes were not materially different from those obtained using non-transformed parameters (Supplementary Table 8). No material changes in causal BMI effects were observed in models using FEF2575/FVC as outcome (Supplementary Table 9), in models restricted to non-asthmatics (Supplementary Table 10), or in models adjusting for study area and education (Supplementary Table 11). No association between change in BMI and change in lung function was observed. The Inverse Probability Weighted analyses did not show material changes in the associations of BMI with FEV1/FVC and FEF2575, although slightly attenuated associations were observed (Supplementary Table 12). Stratified analysis showed negative causal effects in younger age tertiles [(18.2, 35.2) and (35.2, 46.6)] but not in the oldest tertile [(46.6, 61.7)], confirming the MR results found for the Age × BMI interaction (Supplementary Table 13).

## Discussion

The results of this long-term study are consistent with a causal effect of BMI on AO and possibly small airway dysfunction. Higher levels of BMI cause lower levels of FEV1/FVC and FEF2575 up to middle-age, but the effect lessens with aging. The observed Age × BMI interaction, together with the stronger effects observed when instrumenting BMI with SNPs associated with childhood BMI, reflect the complexity of the BMI phenotype in adults. Adult BMI is the result of tracking of BMI over the life course and of genetic influences as well as non-genetic influences on weight change in both childhood and adulthood. Our results suggest that the genetically rooted part of BMI patterns in childhood may be of particular relevance for the level of small airway function and AO later in life, but that this effect diminishes with aging, when exogenous influences on BMI become more relevant.

The observational association between BMI and AO or COPD has not been well-studied. Results from the two largest, post-bronchodilation spirometry based studies are contradictory. In the world-wide BOLD study obesity was less common in persons with AO (12). The opposite was observed in PLATINO study, conducted in Latin American cities (49). The two studies differ in terms of environment, lifestyle and adiposity patterns, but their modifying effect on the BMI-AO association was not reported. SAPALDIA and comparable cohorts previously pointed to important interactions between BMI, physical activity and air pollution with regard to FEV1/FVC and FEF2575 (50–53). In contrast to the BOLD and PLATINO studies, this MR study was based on pre-bronchodilation spirometry. But the observed causal effects of BMI are possibly valid for post-bronchodilation LF, because results did not change after excluding asthmatics (54, 55).

### Composite Nature of BMI Explains the Discrepancy Between Causal and Observational Effects

The current novel results are consistent with confounding in observational obesity- airflow obstruction links. MR and observational regression coefficients were consistent in direction for FEV1/FVC, but not for FEF2575. These parameter-specific differences between observational and causal effects could reflect differences in unmeasured positive confounders. FEV1/FVC and FEF2575, with potentially different etiology, may have different confounders with regard to the association with BMI. The sparsity of model, which included a minimal set of covariates, may be responsible in part for the large difference between observed and causal BMI effects. We cannot exclude entirely that the observed causal interaction with Age may be the result of confounding.

But residual confounding unlikely explains most of the observed difference between causal and observational associations. Another possible explanation of this discrepancy in our data is the composite nature of BMI, which is well-known to be an imprecise measure of different adiposity phenotypes (56), each with distinct genetic and non-genetic components, the contribution of which may vary over the life course. This is a form of measurement error with regard to the true adiposity measure and susceptible time window of interest. In MR studies it is usually assumed an exposure has the same impact on health outcomes, regardless of whether it is due to genetics, or to other sources. This may only hold true for well-defined biological traits, but not for composite exposures like BMI. By refitting the second stage IV model including terms for both, BMI_instrumented_ (genetically determined BMI) and BMI_residual_ (non-genetically determined BMI), we assumed a measurement error model that considers misclassification of the true adiposity measure of interest (see Supplementary Material for a more formalized illustration). The fact that BMI_residual_ showed association with FEF2575 but not with FEV1/FVC, consistent with our observational analysis, supports our measurement error model and points to different effects of non-genetically determined BMI on FEV1/FVC and FEF2575. It is conceivable that genetically determined BMI has negative causal effect, while non-genetically determined BMI has positive effect, and the measurement error due to the metric “BMI” as a mixture of the two components can result in such a discrepancy. A recent metabolomics study reported that genetic score of BMI predicted actual BMI but not the metabolic signature of obesity, indicating that the genetic score captures anthropometric phenotype rather than obesity as a disease trait (57).

Given: (a) the observed negative effect of the genetic, but not of the non-genetic, component of BMI on lung function, (b) that the causal BMI effects were strongest for long-term cross-sectional models, (c) that genetic scores derived from SNPs associated with BMI in childhood led to stronger causal BMI effects, and (d) the observed BMI gene score-age interaction with inverse associations in the younger age groups, our results are consistent with the hypothesis that:

1. BMI in childhood impacts on lung function growth and affects the level of lung function in the first half of life (17), thereby leading to lower levels of attained lung function later in life and increasing the risk of chronic respiratory diseases.
2. BMI in adulthood is increasingly (with age) likely to reflect lifestyle rather than genetic background, which may lead to a different phenotype not well-captured by genetic instruments. This phenotype may have no, or even a positive, effect on lung function, following current discussions about what should be considered a healthy BMI cutoff for older persons.

### Age-Dependent Causal Effects of BMI: Life Course Perspective of Lung Function

As some SNPs were reported to have specific effects on BMI in childhood or divergent BMI effects across the life course (25), the current results may point to specific BMI-related pathways affecting lung function early in life. Besides age-specific genetic effects on BMI (25), age-related differences in the distribution of fat and muscle mass and also in their association with the course of lung function have been reported (56, 58). These age-related differences may reflect changes in gene-environment interactions and the relative contribution of heritability and lifestyle to BMI over the life course (59, 60). The relative contribution of genetically determined BMI to lung function may decrease with aging and the accumulation of molecular damage due to BMI, determined by lifestyle and environmental risks may become more relevant.

Besides the above argued potential effect of BMI in early childhood on lung function growth and its trajectories into adulthood, additional, not mutually exclusive explanations for the observed evidence of causal Age × BMI interactive effects on LF outcomes apply. *First*, it may be a chance finding. *Second*, the results may reflect age-related differences in prevalence and severity of AO. According to the obesity paradox in COPD, excess weight has an adverse effect on the disease course in the early stages. But at more advanced stages for the same degree of AO, obese COPD patients fare better on average than non-obese patients with regard to mortality and hospital admission (4). BMI was positively associated with FEF2575/FVC in heavy smokers with AO (20). *Third*, age-related changes in inflammation, immunologic responses and mechanical lung properties could alter the susceptibility of the airways to obesity (61). Challenges in interpreting low FEV1/FVC in the elderly have been discussed (62). *Fourth*, the observed age-interaction could in part be explained by survivor bias, if survivors with high BMI are those most resistant to the adverse LF effects of obesity. *Finally*, this study does not allow differentiating between causal biological BMI effects on LF and causal BMI effects on phenotypes that are comorbid with LF. The increasing number, but with decreasing effect size, of BMI associated SNPs arising from ever larger GWAS is likely to increase the number of comorbidity signals (24). Although the genetic scores we used in this study did not show association with height, we cannot rule out that the causal BMI effects are in part due to height.

### BMI Effects on FEF2575, a Potential Early Indicator of Small Airway Dysfunction

A causal effect on FEF2575 is of interest, as small airways are frequently involved at an early stage in COPD and asthma (18) and they have been shown to be adversely affected by weight and growth patterns in early childhood (17). Adverse peripheral airway effects of excess weight were demonstrated by impulse oscillometry (15, 63). The insensitivity of spirometry to peripheral airway abnormalities may in part explain the contradictory findings on the BMI- LF association (63). The value of FEF2575 for early detection of small airway dysfunction has been questioned (64, 65), and attributed to the parameter's wide variability in healthy subjects (66). But several aspects of this study justify the consideration of FEF2575 as an independent phenotype. The partial correlations with FEV1, FVC, and FEV1/FVC were between 0.182 (FEF2575: FVC at SAP1) and 0.868 (FEF2575_s1, s2, s3_: FEV1/FVC _s1, s2, s3_) across phenotypes and time points. The intra-individual variability of FEF2575 was smaller than that of FEV1/FVC. FEF2575 was previously correlated with functional imaging assessment of small airway function (67). In obliterative bronchiolitis, the paradigm of small airway disease, FEF2575 is considered a sensitive diagnostic marker (68). FEF2575 was correlated with smooth muscle α-actin in the small airways, a marker of airway remodeling (69), and predicted mortality from COPD after 20 years of follow-up (70).

### Strengths and Limitations

As in any study, results have to be evaluated in the light of strengths and limitations. The assumptions of MR appeared to be satisfied, strengthening the choice of carrying out an MR study. The MR assumptions could still be violated by unobserved confounders, though. Statistical power of this study was limited, but the choice of considering medium- and long-term averages, for both exposure and outcome, alleviated this problem and allowed studying the stability and age dependency of causal effects. This study did not confirm previously reported causal effects of BMI on FEV1 and FVC (2) that were strictly cross-sectional and based on data from a single time point. The sample size did not allow studying the causality of BMI effects in respiratory health subgroups, e.g., COPD patients. But the detailed participant characterization in SAPALDIA, an internationally renowned respiratory cohort (33, 71–73) allowed excluding persons with a self-report of doctor-diagnosed asthma at any point during 20 years. Additional limitations include the restriction of LF to pre-bronchodilation, whereas post-bronchodilation FEV1/FVC forms the basis for diagnosing COPD (74), and of obesity assessment to BMI in the absence of visceral adiposity indicators (68). No measurements of BMI in childhood of SAPALDIA participants were available, which would have allowed to instrument childhood BMI. We were limited in assessing longitudinal effects of BMI or its change on LF decline in adults. Genetic variants to instrument BMI change do not exist. Many more than three time points would be needed to truly assess causal BMI effects on LF change over time. But biological pathways underlying level of LF and LF decline may differ. BMI and lung function averaged over a certain time period as in this study may be better measures for assessing chronic long-term associations between the two, given the intra-individual volatility of these parameters over time. This is supported by the fact that we found stronger associations by using medium- and long-term averages, compared to single time point associations, and a higher predictive ability when compared with that of BMI change with lung function change. We acknowledge that by taking averages of BMI and averages of lung function we are faced with the problem that persons with higher BMI at baseline and lower BMI at follow-up may have the same long-term BMI average as persons with lower BMI at baseline and higher BMI at follow-up. The same caveat may apply for two people with the same average of lung function. Because this adds to the problem of reverse causation (and would most likely bias the associations toward the null), we were also taking a predictive approach of investigating the associations of BMI averaged over SAP1 and SAP2 with lung function averaged over SAP2 and SAP3. As another limitation, we acknowledge that our study did not investigate non-linearity of the causal effects. Finally, we cannot exclude that the complete case analysis led to some bias due to other sources of missingness, although attrition seems to be by far the most important mechanism generating missingness in our data. Although the Inverse Probability Weighted analysis considered bias due to the most important attrition factor, and for that matter a major mortality determinant, namely smoking, not all factors influencing non-participation could be considered. However, the attrition bias would likely bias the associations toward the null, given that the dropouts would more likely have experienced increase in BMI and decline in LF.

## Conclusion

The results of this study suggest that AO and possibly small airways disease may, in part, be the result of excess weight in young and middle-aged adults, or even in children. The results need to be confirmed in the context of a larger MR study involving tests reflecting small airway dysfunction and more specific parameters for adiposity at different stages in life. In addition, the study points to important methodological needs in future studies on the causal effects of obesity and lung health, namely to consider adiposity- and lung phenotype-specific associations from a life course perspective and to derive and apply genetic instruments reflecting more specific obesity phenotypes.

## Data Availability Statement

Data included in this manuscript is available from the corresponding author upon a justified request.

## Ethics Statement

The studies involving human participants were reviewed and approved by the Swiss Academy of Medical Sciences and the Cantonal Ethics Committees. The participants provided their written informed consent to participate in this study.

## Author Contributions

NP-H designed the SAPALDIA cohort. NP-H, AJ, CS, and GL developed the analysis plan for this paper. AJ and GL conducted the statistical analysis. NP-H and GL drafted the manuscript. NP-H, MI, DS, MP, PS, and RB conducted the SAPALDIA study. All authors read and corrected the manuscript and approved of the final version.

## Conflict of Interest

DS obtained funding for grants from the following companies: Astra-Zeneca, PanGas, Wenmann, Curetis, Boston Scientific, Cucassia, and also from Swiss Lung Leagues. DS was paid for lectures including service on speaker's bureaus by the following companies: Astra-Zeneca, Novartis, GSK, Roche, Zambon, Pfizer, Schwabe Pharma and Vifor. The remaining authors declare that the research was conducted in the absence of any commercial or financial relationships that could be construed as a potential conflict of interest.
