# Supplementary material for: Causal Effects of Body Mass Index on Airflow Obstruction and Forced Mid-Expiratory Flow: A Mendelian Randomization Study Taking Interactions and Age-Specific Instruments Into Consideration Toward a Life Course Perspective
Source: Front Public Health. 2021 May 11;9:584955. doi: 10.3389/fpubh.2021.584955 (PMC8144328; doi:10.3389/fpubh.2021.584955)
Supplement: Supplementary file 1 [file Data_Sheet_1.pdf]

Supplemental Material

Authors: Probst-Hensch N[1,2,*], Jeong A[1,2], Stolz D[3], Pons M[4], Soccal PM[5], Bettschart R[6], Jarvis D[7,8], Holloway JW[9], Kronenberg F[10], Imboden M[1,2], Schindler C[1,2], Lovison GF[1,2,11]

Affiliations:

[1] Department of Epidemiology and Public Health, Swiss Tropical and Public Health Institute, Basel, Switzerland

[2] Department of Public Health, University of Basel, Switzerland

[3] Clinic of Pulmonary Medicine and Respiratory Cell Research, University Hospital Basel, Basel,

[4] Division of Pulmonary Medicine, Regional Hospital of Lugano, Lugano, Switzerland

[5] Division of Pulmonary Medicine, Geneva University Hospitals, Geneva, Switzerland

[6] Lungenpraxis Aarau, Hirslanden Klinik, Aarau, Switzerland

[7] Medical Research Council-Public Health England Centre for Environment and Health, Imperial College London, London, UK

[8] Population Health and Occupational Disease, National Heart and Lung Institute, Imperial College London, London, UK

[9] Human Development and Health, Faculty of Medicine, University of Southampton, UK

[10] Division of Genetic Epidemiology, Department of Medical Genetics, Molecular and Clinical Pharmacology, Medical University of Innsbruck, Innsbruck, Austria

[11] Department of Economics, Business and Statistics, University of Palermo, Italy

Corresponding author:

N. Probst-Hensch, Head Department of Epidemiology and Public Health, Socinstrasse 57, 4002 Basel, Nicole.probst@swisstph.ch

Methods

Study sample.

Where information was available (SAP2 only), participants with CRP ≥ 10 were excluded to minimize the influence of acute infections.

Lung function testing.

Spirometry was conducted according to American Thoracic Society recommendations (1). Spirometers were calibrated daily with a 3-L syringe at each participating center. Accuracy was verified and recorded daily and was systematically within the mandatory range of 3%.

Statistical analysis.

Statistical analyses were performed within the programming environment R, version 3.4.3 for Windows (http://www.r-project.org/). In particular, IV-analyses were performed using the package "AER", and the model selection procedures carried out to check the MR assumptions were performed using, besides the "stats" and "MASS" standard packages, the R packages "gamlss" (for Zero-Adjusted Gamma distributed responses) and "betareg" (for Beta distributed responses). "MendelianRandomization" package was used for MR Egger regression. The p-values are two-tailed; effect estimates are presented as β-coefficients and 95% confidence intervals.

Evaluating the difference between Causal Effects and Observational Associations

We would like to show that the large discrepancy found in our study between causal effects and observational effects of BMI on LF variables may be due to the "composite" nature of BMI as a measure of obesity, being a mixture of components with different effects on specific lung function parameters.

Suppose, for the sake of simplicity, that BMI measures the mixture of two biological traits (say childhood obesity CO and non-childhood obesity NCO); moreover, suppose that these two traits are uncorrelated and there is no erratic component (like, for example, strict instrumental measurement error, or any other component different from CO and NCO):

$$BMI = \alpha 1\ CO + \alpha 2\ NCO \qquad (1)$$

Suppose also that two simple linear models capture the causal effects (β_{CO} and β_{NCO}, respectively) of the CO and NCO components on LF:

$$LF = \beta_{0CO} + \beta_{CO}\ CO + \eta$$

$$LF = \beta_{0NCO} + \beta_{NCO}\ NCO + \delta$$

We would like to analyse only the causal effect of the CO component, but we have only available data on LF and the “composite” BMI, so we have to analyse the "composite exposure" model:

$$LF = \gamma_0 + \gamma\ BMI + \varepsilon$$

Notice that this is an example of “measurement error model”, using the econometric and psychometric terminology. It is not a “traditional” type of measurement error (the error due to an imprecise measurement instrument), but it is considered anyway a form of measurement error, because we would like to measure CO, and instead we have to use BMI, which is an imperfect measure of CO (being a “mixture” of CO and NCO). In that sense it addresses a type of measurement error that is similar to the measurement error problem addressed by our long-term average approach: measurement error in the sense of misclassification of the true exposure of interest.

It can be shown that the OLS estimator of γ (the associational parameter estimated in our “Observational association analysis" Section) can be written as:

$$\hat{\gamma} = \hat{\beta}_{CO}\ \alpha 1\ SS_{CO} + \hat{\beta}_{NCO}\ \alpha 2\ SS_{NCO} \qquad (2)$$

where SS_{CO} is the proportion of BMI variance explained by the CO component and SS_{NCO} is

the proportion of BMI variance explained by the NCO component. Notice that, by the combination of different α1, α2 values (the weights of the CO and NCO component in the "mixture" BMI) and different SS_{CO}, SS_{NCO} values (the proportion of total variability of BMI attributable to the two components), it may very well happen that, although the estimated causal effect of CO ($\hat{\beta}_{CO}$) is negative, the estimated associational effect ($\hat{\gamma}$) is either positive (if the positive NCO effect dominates the negative CO effect), or close to zero (if the two effects counterbalance each other).

Finally, suppose the genetic score BMI_{gs} predicts only the CO component of BMI, and that the effect of the CO component on LF is negative. Then, of course, the IV estimate $\hat{\beta}_{CO;IV}$ will be negative, because it estimates β_{CO}, which is negative, while the estimated associational effect ($\hat{\gamma}$) can be significantly positive (this is what happens in our analysis of FEF2575) or not significantly different from 0 (this is what happens in our analysis of the FEV1/FVC ratio), owing to the combination of values in the $\hat{\gamma}$ formula (2).

It is worth stressing that this simple "composite exposure" model is also consistent with our finding that the Age:BMI interaction must be included in the final model used in MR. In fact, it is reasonable to assume that the two weights α1, α2, and the two proportions SS_{CO}, SS_{NCO}, in formula (2) depend on Age, reflecting the fact that the variance in BMI explained by genetics may vary (typically decreasing) by age and that BMI phenotypes may also differ across age. If this age-dependence is present, it would result in a significant Age×BMI interaction in the final MR model, which is exactly what we have found in our data.

Obviously, we cannot disentangle the distinct contributions of CO and NCO in (1), and hence in (2). But, if our assumption that the genetic score BMI_{gs} predicts only, or mainly, the CO component of BMI holds at least approximately, then we have an indirect way of assessing these distinct contributions. Let $BMI_{instrumented}$ be the part of BMI predicted by BMI genetic

score and $BMI_{residual}$ be the remaining part, not predicted by BMI genetic score. Then the decomposition BMI = $BMI_{instrumented}$ + $BMI_{residual}$ under the abovementioned assumption provides an estimated version of (1). We can assess the relative contributions of these two components by including both of them in the IV second stage model for LF, unlike what we do in MR analysis, where we include only $BMI_{instrumented}$.

The plausibility of the "composite exposure" explanation of the discrepancy between MR causal effects and observational associations is confirmed by the supplementary analyses that we have carried out based on this indirect approach (see Supplement Table 6):

- when we include both $BMI_{instrumented}$ and $BMI_{residual}$ in the second stage model of the 2SLS method of IV estimation for FEF2575, $BMI_{instrumented}$ has a significant negative effect on LF, while $BMI_{residual}$ has a highly significant positive effect on LF; since $BMI_{residual}$ represents more than 90% of observed BMI, the aggregate result is the positive observational association we have reported (see Table 3);
- when we include both BMI_{instr} and BMI_{res} in the second stage model of the 2SLS method of IV estimation for FEV1/FVC, BMI_{instr} has again a significant negative effect on LF, while BMI_{res} has a very small, non-significant effect on LF; the aggregate result is in this case the non significant observational association we have reported (see Table 5 in main text).

Checking MR assumptions.

In preparing for the MR analysis, a set of MR assumptions as highlighted by Van der Weele et al.(12) were checked. In all these checks, the models used were chosen through a selection procedure carried out within the class of (extended) Generalised Linear Models, with the aim of making the choice more flexible and finding the model most appropriate in terms of both

distribution of response and possible non-linearity of the relationship of the response with the predictors.

This approach may lead to select models which are not linear and Gaussian, unlike those used in the MR analysis described earlier. The justification for the choice of models that may differ from the Linear Gaussian models used in the final MR analysis is that while in such final analysis the emphasis is in using Instrumental Variables methods, which are well developed only in the Linear Gaussian context, in checking the MR assumption the emphasis is on employing the best models to obtain the soundest possible statistical support needed for justifying the use of MR, especially in view of the limited sample sizes available in our study.

(i) Checking MR assumption 1: the genetic score is associated with the exposure

In the context of our study, MR Assumption 1 states that, to be a valid instrument, the BMI genetic score must be associated with the two exposures used in the final models: i.e. the average of BMI over SAP1-SAP2 (prediction model) and the average of BMI over SAP1-SAP2-SAP3 (long-term cross-sectional model). The best models for these checks turned out to be LogNormal/Identity (e.g. linear Gaussian models for the log-transformed BMI averages), which agrees with the choice of log-transforming the BMI averages in the outcome models (1) and (4).

(ii) Checking MR assumption 2: the genetic score is not associated with confounders of the exposure–outcome relationship

In our study, MR Assumption 2 states that BMI genetic score should be independent of factors (measured and unmeasured) that may confound the relationship between BMI and lung function: in our case Packyears and Height. SNPs that have been reported as associated

with smoking related phenotypes were excluded from computing BMI genetic scores. A GLM-type model with Zero-adjusted Gamma response was chosen to test for BMI genetic score association with Packyears, due to the peculiar shape of the distribution of the variables $Packyears_{s1}$, $Packyears_{s2}$, $Packyears_{s3}$ (Supplement Figure 7), which is characterized by strong positive skewness and an excess of zeroes, corresponding to never smoking participants. The Zero-adjusted Gamma distribution has three parameters: the mean μ and the dispersion σ of the Gamma component, plus Pr(0), the probability of taking up the value 0. Therefore, three link functions and three linear predictors had to be selected for models using this as response distribution (see Supplement Table 5). Linear Gaussian model was chosen to test for BMI genetic score association with Height.

To further investigate potential pleiotropy, we conducted MR Egger analysis, using the same final models. MR Egger intercept from zero with $p<0.05$ indicates potential pleiotropy.

We also interrogated whether Age and Sex modify the influence of BMI genetic score on phenotypic BMI by regressing the BMI averages on a linear predictor including Age (averaged over SAP1-SAP2 and SAP1-SAP2-SAP3, respectively and centered at 18 years), Sex, BMI genetic score and all their interactions.

(iii) Checking MR assumption 3: the genetic score is not associated with the outcome, conditional on the exposure and confounders of the exposure–outcome relationship.

Finally, we tested MR Assumption 3, which in our study requires conditional independence of BMI genetic score and lung function outcomes, given observed BMI and (observed) confounders. FEF2575 and FEV1/FVC averages were regressed on a linear predictor containing BMI genetic score, Sex, Packyears, observed BMI, Age and Height (the latter three variables included as averages over the appropriate period).

Results

Checking the MR assumptions

(i) Checking MR assumption 1

When assessing the association between BMI genetic score and BMI_{s1s2} and BMI_{s1s2s3}, the best model selected was LogNormal/Identity (i.e a linear Gaussian model for the log-transformed BMI averages) (Supplement Table 47(a)). BMI genetic score was found to be a predictor of BMI exposures. R^2 values for the predictive (0.0193) and the long-term cross-sectional (0.0137) models were in line with those published in previous MR studies (8)

(ii) Checking MR assumption 2

There was no evidence of an association between BMI genetic scores and smoking history (Supplement Table 4(b)) and therefore relevant pleiotropic effects of the BMI genetic score on this potential confounder can be excluded. The lacking BMI genetic score-smoking association agrees with our investigation into the overlap of the BMI-related SNPs with the SNPs known to be associated with smoking behaviour. We identified 154 SNPs associated either with smoking intensity (cigarettes smoked/day) or smoking initiation (ever versus never smokers) from the GWAS combining three consortia, i.e. ENGAGE (European Network for Genetic and Genomic Epidemiology), TAG (Tobacco and Genetics Consortium), and Ox-GSK (Oxford-GlaxoSmithKline study) (14-16). While none of the 32 SNPs included in the Speliotes score overlap with the 154 smoking-related SNPs, one of them (rs10767664) was in high LD with several SNPs in BDNF (brain derived neurotrophic factor) (R^2=0.681~ 0.911), that were associated with smoking initiation. In SAPALDIA subjects, however this SNP showed no association with smoking initiation with or without adjustment for age, sex, and BMI. None of the 12 SNPs included in the Felix Score overlapped with the

154 smoking-related SNPs, while one SNP (rs6265) included in the Yengo score overlapped and hence excluded. 2 and 16 further SNPs were excluded from computing Felix and Yengo score, respectively, due to known association with smoking phenotypes in PhenoScanner. MR Egger regression indicated no pleiotropy for SNPs included in Yengo score. For Speliotes and Felix scores, however, slight indication of pleiotropy was observed for Age:BMI interaction effect for FEV1/FVC and FEF2575 predictive models for Speliotes and FEF2575 long-term cross-sectional model for Felix (Supplement Table 5 and Figures 4-6).

We did not observe age, sex or their combination to modify the association of BMI genetic score with phenotypic BMI (data not shown).

(iii) Checking MR assumption 3

The hypothesis of conditional independence of BMI genetic score and both FEF2575 and FEV1/FVC, given phenotypic BMI, age, sex, height and packyears, was not rejected (results not shown). This conclusion was independent of the distribution/link function used and slightly more strongly supported for FEF2575, in line with the observation that BMI genetic score is a stronger instrument for FEF2575.

In summary, the assumptions of MR appeared to be satisfied. As well known, this cannot be taken as conclusive evidence of the validity of BMI genetic score as an instrument. All our tests can only be performed on observed confounders and therefore cannot rule out the possibility of violations of the MR assumptions due to unobserved confounding. It nevertheless strengthens our choice of carrying out a Mendelian Randomization study based on the given genetic score.

Sensitivity analysis.

The comparison of regression estimates for BMI_{s1}, BMI_{s2}, and BMI_{s3} on the BMI genetic score confirmed that BMI_{s1} derived from self-reported weight can be reliably used. The regression coefficients (β =0.0335 (s.d.0.0041)) and the R^2 measure (R^2 = 0.01578) were not materially different from those for SAP2 (β =0.0402 (s.d.0.0045); R^2 = 0.01869) and for SAP3 (β =0.0322 (s.d.0.0050); R^2 = 0.01125). The intermediate level of the estimated regression coefficient for SAP1, which contradicts a decreasing association of BMI with BMI gene score with age, suggests that the use of self-reported weight might have slightly biased downward the relationship (Supplement Table 7).

Supplement Table 1. Single nucleotide polymorphisms and weights considered for deriving genetic BMI score

Supplement Table_1.xlsx, which contains lists of SNPs used to compute the three BMI genetic scores can be found at journal webpage.

Supplement Table 2. Correlation matrices for BMI genetic scores

a) Long-term cross-sectional model (N = 2728)

	Speliotes (32 SNPs)	Yengo (862 SNPs)	Felix (12 SNPs)
Speliotes	1.000	0.415	0.551
Yengo		1.000	0.284
Felix			1.000

b) Prediction model (N = 2850)

	Speliotes (32 SNPs)	Yengo (862 SNPs)	Felix (12 SNPs)
Speliotes	1.000	0.414	0.543
Yengo		1.000	0.282
Felix			1.000

Supplement Table 3. Partial correlation matrices for FEV1, FVC, FEV1/FVC, FEF2575, and FEF2575/FVC (adjusted for Age, Sex and Height) at different time points and averaged over SAP2-SAP3 and SAP1-SAP2-SAP3

a) Partial auto-correlations over time of each lung function variable

	$FEV1_{s1}$	$FEV1_{s2}$	$FEV1_{s3}$
$FEV1_{s1}$	1.000	0.759	0.717
$FEV1_{s2}$		1.000	0.831
$FEV1_{s3}$			1.000

	FVC_{s1}	FVC_{s2}	FVC_{s3}
FVC_{s1}	1.000	0.713	0.617
FVC_{s2}		1.000	0.730
FVC_{s3}			1.000

	$FEV1/FVC_{s1}$	$FEV1/FVC_{s2}$	$FEV1/FVC_{s3}$
$FEV1/FVC_{s1}$	1.000	0.682	0.563
$FEV1/FVC_{s2}$		1.000	0.725
$FEV1/FVC_{s3}$			1.000

	$FEF2575_{s1}$	$FEF2575_{s2}$	$FEF2575_{s3}$
$FEF2575_{s1}$	1.000	0.711	0.647
$FEF2575_{s2}$		1.000	0.766
$FEF2575_{s3}$			1.000

	$FEF2575/FVC_{s1}$	$FEF2575/FVC_{s2}$	$FEF2575/FVC_{s3}$
$FEF2575/FVC_{s1}$	1.000	0.665	0.533
$FEF2575/FVC_{s2}$		1.000	0.671
$FEF2575/FVC_{s3}$			1.000

b) Same-time partial correlations between pairs of lung-function variables (or averages over SAP2-SAP3 and SAP1-SAP2-SAP3)

	FVC_{s1}	FVC_{s2}	FVC_{s3}	FVC_{s2s3}	FVC_{s1s2s3}
$FEV1_{s1}$	0.781				
$FEV1_{s2}$		0.792			
$FEV1_{s3}$			0.841		
$FEV1_{s2s3}$				0.813	
$FEV1_{s1s2s3}$					0.807

	$FEV1/FVC_{s1}$	$FEV1/FVC_{s2}$	$FEV1/FVC_{s3}$	$FEV1/FVC_{s2s3}$	$FEV1/FVC_{s1s2s3}$
$FEV1_{s1}$	0.432				
$FEV1_{s2}$		0.519			
$FEV1_{s3}$			0.548		
$FEV1_{s2s3}$				0.548	
$FEV1_{s1s2s3}$					0.510

	$FEF2575_{s1}$	$FEF2575_{s2}$	$FEF2575_{s3}$	$FEF2575_{s2s3}$	$FEF2575_{s1s2s3}$
$FEV1_{s1}$	0.716				
$FEV1_{s2}$		0.726			
$FEV1_{s3}$			0.762		
$FEV1_{s2s3}$				0.769	
$FEV1_{s1s2s3}$					0.766

	$FEF2575/FVC_{s1}$	$FEF2575/FVC_{s2}$	$FEF2575/FVC_{s3}$	$FEF2575/FVC_{s2s3}$	$FEF2575/FVC_{s1s2s3}$
$FEV1_{s1}$	0.352				
$FEV1_{s2}$		0.423			
$FEV1_{s3}$			0.519		
$FEV1_{s2s3}$				0.511	
$FEV1_{s1s2s3}$					0.475

	$FEV1/FVC_{s1}$	$FEV1/FVC_{s2}$	$FEV1/FVC_{s3}$	$FEV1/FVC_{s2s3}$	$FEV1/FVC_{s1s2s3}$
FVC_{s1}	-0.201				
FVC_{s2}		-0.082			
FVC_{s3}			0.054		
FVC_{s2s3}				-0.010	
FVC_{s1s2s3}					-0.070

	$FEF2575_{s1}$	$FEF2575_{s2}$	$FEF2575_{s3}$	$FEF2575_{s2s3}$	$FEF2575_{s1s2s3}$
FVC_{s1}	0.182				
FVC_{s2}		0.215			
FVC_{s3}			0.362		
FVC_{s2s3}				0.309	
FVC_{s1s2s3}					0.286

	$FEF2575/FVC_{s1}$	$FEF2575/FVC_{s2}$	$FEF2575/FVC_{s3}$	$FEF2575/FVC_{s2s3}$	$FEF2575/FVC_{s1s2s3}$
FVC_{s1}	-0.227				
FVC_{s2}		-0.144			
FVC_{s3}			0.063		
FVC_{s2s3}				-0.011	
FVC_{s1s2s3}					-0.071

	$FEF2575_{s1}$	$FEF2575_{s2}$	$FEF2575_{s3}$	$FEF2575_{s2s3}$	$FEF2575_{s1s2s3}$
$FEV1/FVC_{s1}$	0.846				
$FEV1/FVC_{s2}$		0.867			
$FEV1/FVC_{s3}$			0.856		
$FEV1/FVC_{s2s3}$				0.868	
$FEV1/FVC_{s1s2s3}$					0.868

	$FEF2575/FVC_{s1}$	$FEF2575/FVC_{s2}$	$FEF2575/FVC_{s3}$	$FEF2575/FVC_{s2s3}$	$FEF2575/FVC_{s1s2s3}$
$FEV1/FVC_{s1}$	0.933				
$FEV1/FVC_{s2}$		0.928			
$FEV1/FVC_{s3}$			0.925		
$FEV1/FVC_{s2s3}$				0.937	
$FEV1/FVC_{s1s2s3}$					0.946

	$FEF2575/FVC_{s1}$	$FEF2575/FVC_{s2}$	$FEF2575/FVC_{s3}$	$FEF2575/FVC_{s2s3}$	$FEF2575/FVC_{s1s2s3}$
$FEF2575_{s1}$	0.885				
$FEF2575_{s2}$		0.902			
$FEF2575_{s3}$			0.916		
$FEF2575_{s2s3}$				0.916	
$FEF2575_{s1s2s3}$					0.906

Supplement Table 4. Tests for checking the MR assumptions

(a) Assumption 1: Associations of BMI genetic score with observed BMI averages

	**Speliotes (2010) BMI genetic score**					
	N	Coefficient	SE	F-statistics	p-value	Best model
$BMI_{s1,s2}$	4147	0.0050	0.0006	73.89	0.000	logNormal/identity (Linear Gaussian model for log-transformed response)
$BMI_{s1,s2,s3}$	3499	0.0041	0.0006	41.50	0.000	logNormal/identity (Linear Gaussian model for log-transformed response)
	**Yengo (2018) BMI genetic score**					
	N	Coefficient	SE	F-statistics	p-value	Best model
$BMI_{s1,s2}$	4147	0.0018	0.0001	232.86	0.000	logNormal/identity (Linear Gaussian model for log-transformed response)
$BMI_{s1,s2,s3}$	3499	0.0017	0.0001	181.98	0.000	logNormal/identity (Linear Gaussian model for log-transformed response)
	**Felix (2016) BMI genetic score**					
	N	Coefficient	SE	F-statistics	p-value	Best model
$BMI_{s1,s2}$	4147	0.0066	0.0011	34.77	0.000	logNormal/identity (Linear Gaussian model for log-transformed response)
$BMI_{s1,s2,s3}$	3499	0.0055	0.0012	20.37	0.000	logNormal/identity (Linear Gaussian model for log-transformed response)

(b) Assumption 2: Associations of BMI genetic score with Packyears smoked and Height

**Speliotes (2010) BMI genetic score**					
	N	Coefficient	SE	p-value	Best GLM model
$Packyears_{s2}$ [Log(μ)]	4039	-0.0021	0.0059	0.714	Zero-adjusted Gamma with log/log/logit links, constant dispersion and constant Pr(0)
$Packyears_{s3}$ [Log(μ)]	3539	-0.0048	0.0064	0.451	Zero-adjusted Gamma with log/log/logit links, constant dispersion and constant Pr(0)
$Height_{s1,s2}$	4149	0.0051	0.037	0.889	Classical Gaussian linear model
$Height_{s1,s2,s3}$	3500	0.011	0.040	0.779	Classical Gaussian linear model
**Yengo (2018) BMI genetic score**					
	N	Coefficient	SE	p-value	Best GLM model
$Packyears_{s2}$ [Log(μ)]	4039	0.0019	0.0011	0.086	Zero-adjusted Gamma with log/log/logit links, constant dispersion and constant Pr(0)
$Packyears_{s3}$ [Log(μ)]	3539	0.0017	0.0013	0.191	Zero-adjusted Gamma with log/log/logit links, constant dispersion and constant Pr(0)
$Height_{s1,s2}$	4149	-0.0092	0.0074	0.213	Classical Gaussian linear model
$Height_{s1,s2,s3}$	3500	-0.0053	0.0081	0.514	Classical Gaussian linear model
**Felix (2016) BMI genetic score**					
	N	Coefficient	SE	p-value	Best GLM model
$Packyears_{s2}$ [Log(μ)]	4039	0.0067	0.0109	0.541	Zero-adjusted Gamma with log/log/logit links, constant dispersion and constant Pr(0)
$Packyears_{s3}$ [Log(μ)]	3539	0.0045	0.0118	0.704	Zero-adjusted Gamma with log/log/logit links, constant dispersion and constant Pr(0)
$Height_{s1,s2}$	4149	0.028	0.071	0.695	Classical Gaussian linear model
$Height_{s1,s2,s3}$	3500	0.030	0.077	0.695	Classical Gaussian linear model

Supplement Table 5. MR Egger regression results for intercept

Speliotes (2010) BMI genetic score				
		Estimate	SE	p-value
**FEV1/FVC**	**Predictive model**			
	BMI main effect	-0.002	0.001	0.095
	BMI*Age interaction effect	-0.003	0.001	0.014
	**Long-term cross-sectional model**			
	BMI main effect	-0.001	0.002	0.479
	BMI*Age interaction effect	-0.003	0.002	0.142
**FEF2575**	**Predictive model**			
	BMI main effect	-0.020	0.021	0.351
	BMI*Age interaction effect	-0.041	0.020	0.047
	**Long-term cross-sectional model**			
	BMI main effect	-0.013	0.028	0.646
	BMI*Age interaction effect	-0.028	0.027	0.305
**FEF2575/FVC**	**Predictive model**			
	BMI main effect	-0.009	0.019	0.642
	BMI*Age interaction effect	-0.010	0.018	0.590
	**Long-term cross-sectional model**			
	BMI main effect	-0.005	0.024	0.835
	BMI*Age interaction effect	-0.006	0.022	0.767
**Yengo (2018) BMI genetic score**				
		Estimate	SE	p-value
**FEV1/FVC**	**Predictive model**			
	BMI main effect	0.000	0.000	0.526
	BMI*Age interaction effect	0.000	0.000	0.934
	**Long-term cross-sectional model**			
	BMI main effect	0.000	0.000	0.240
	BMI*Age interaction effect	0.000	0.000	0.447
**FEF2575**	**Predictive model**			
	BMI main effect	0.002	0.004	0.669
	BMI*Age interaction effect	0.000	0.004	0.949
	**Long-term cross-sectional model**			
	BMI main effect	-0.007	0.005	0.111
	BMI*Age interaction effect	-0.002	0.004	0.730
**FEF2575/FVC**	**Predictive model**			
	BMI main effect	0.001	0.004	0.828
	BMI*Age interaction effect	0.000	0.003	0.972
	**Long-term cross-sectional model**			
	BMI main effect	-0.001	0.004	0.783
	BMI*Age interaction effect	0.000	0.004	0.945

Felix (2016) BMI genetic score				
		Estimate	SE	p-value
**FEV1/FVC**	**Predictive model**			
	BMI main effect	-0.002	0.002	0.473
	BMI*Age interaction effect	-0.004	0.003	0.184
	**Long-term cross-sectional model**			
	BMI main effect	-0.001	0.003	0.707
	BMI*Age interaction effect	-0.007	0.003	0.052
**FEF2575**	**Predictive model**			
	BMI main effect	-0.024	0.035	0.504
	BMI*Age interaction effect	-0.056	0.047	0.234
	**Long-term cross-sectional model**			
	BMI main effect	0.032	0.035	0.365
	BMI*Age interaction effect	-0.098	0.048	0.039
**FEF2575/FVC**	**Predictive model**			
	BMI main effect	-0.006	0.032	0.855
	BMI*Age interaction effect	-0.015	0.040	0.704
	**Long-term cross-sectional model**			
	BMI main effect	0.001	0.035	0.977
	BMI*Age interaction effect	-0.024	0.048	0.613

Supplement Table 6. Causal effect[1] of BMI instrumented and BMI residual on FEV1/FVC and FEF2575 in predictive and in long-term cross-sectional models. BMI genetic score: Speliotes (5); 32 SNPs)

	N	β_c	SE	p-value
**FEV1/FVC**				
**Predictive model**				
BMI instrumented main effect[2]	2853	-0.561	0.235	0.017
BMI residual main effect[3]		0.001	0.024	0.953
**Long-term cross-sectional model**				
BMI instrumented main effect	2731	-0.752	0.280	0.007
BMI residual main effect		0.040	0.026	0.135
**FEF2575**				
**Predictive model**				
BMI instrumented main effect	2850	-7.152	3.148	0.023
BMI residual main effect		0.865	0.332	0.009
**Long-term cross-sectional model**				
BMI instrumented main effect	2728	-9.251	3.952	0.019
BMI residual main effect		0.635	0.376	0.092

[1] β_{c1}: causal BMI main effect per one BMI-increasing allele; β_{c2}: causal BMI*Age interaction effect per one BMI-increasing allele.

The negative sign of the causal main effect means that, keeping all other predictors fixed, at age 18 (which has been chosen as the origin in our analysis) BMI has a causal negative effect on LF. The positive sign of the Age:BMI causal interactive effect implies that, as age increases, the detrimental effect of BMI on LF decreases. As a consequence, the total effect of BMI becomes null at middle ages and protective at older ages.

Supplement Table 7. BMI at SAP1, SAP2 and SAP3 regressed on BMI genetic score

**Speliotes (2010) BMI genetic score**					
	N	BMI genetic score coefficient	SE	p-value	R^2
BMI_{s1}	4153	0.0045	0.0006	0.000	0.015
BMI_{s2}	4160	0.0054	0.0006	0.000	0.017
BMI_{s3}	3511	0.0041	0.0007	0.000	0.009
**Yengo (2018) BMI genetic score**					
	N	BMI genetic score coefficient	SE	p-value	R^2
BMI_{s1}	4153	0.0016	0.0001	0.000	0.045
BMI_{s2}	4160	0.0019	0.0001	0.000	0.053
BMI_{s3}	3511	0.0018	0.0001	0.000	0.045
**Felix (2016) BMI genetic score**					
	N	BMI genetic score coefficient	SE	p-value	R^2
BMI_{s1}	4153	0.0062	0.0011	0.000	0.007
BMI_{s2}	4160	0.0070	0.0012	0.000	0.008
BMI_{s3}	3511	0.0054	0.0014	0.000	0.004

Supplement Table 8. Causal effects[1] of BMI on FEF2575 and FEV1/FVC in predictive and in long-term cross-sectional models: sensitivity analysis with log-transformed (for FEF2575) and logit-transformed (for FEV1/FVC) outcomes.

**Speliotes (2010) BMI genetic score**						
	β_{c1}	SE	p-value	β_{c2}	SE	p-value
**FEV1/FVC**						
$\log(BMI_{s1s2}) \rightarrow \text{logit}(FEV1/FVC_{s2,s3})$	-2.366	1.316	0.072	0.080	0.054	0.138
$\log(BMI_{s1s2s3}) \rightarrow \text{logit}(FEV1/FVC_{s1,s2,s3})$	-3.603	1.693	0.033	0.099	0.056	0.076
**FEF2575**						
$\log(BMI_{s1s2}) \rightarrow \log(FEF2575_{s2,s3})$	-3.080	1.536	0.045	0.108	0.063	0.086
$\log(BMI_{s1s2,s3}) \rightarrow \log(FEF2575_{s1,s2,s3})$	-3.489	1.690	0.039	0.100	0.056	0.073
**Yengo (2018) BMI genetic score**						
	β_{c1}	SE	p-value	β_{c2}	SE	p-value
**FEV1/FVC**						
$\log(BMI_{s1s2}) \rightarrow \text{logit}(FEV1/FVC_{s2,s3})$	-0.716	0.624	0.252	0.022	0.022	0.312
$\log(BMI_{s1s2s3}) \rightarrow \text{logit}(FEV1/FVC_{s1,s2,s3})$	-1.221	0.739	0.100	0.027	0.022	0.217
**FEF2575**						
$\log(BMI_{s1s2}) \rightarrow \log(FEF2575_{s2,s3})$	-1.083	0.706	0.125	0.039	0.025	0.119
$\log(BMI_{s1s2,s3}) \rightarrow \log(FEF2575_{s1,s2,s3})$	-1.161	0.718	0.106	0.031	0.021	0.148
**Felix (2016) BMI genetic score**						
	β_{c1}	SE	p-value	β_{c2}	SE	p-value
**FEV1/FVC**						
$\log(BMI_{s1s2}) \rightarrow \text{logit}(FEV1/FVC_{s2,s3})$	-2.472	2.402	0.300	0.058	0.082	0.480
$\log(BMI_{s1s2s3}) \rightarrow \text{logit}(FEV1/FVC_{s1,s2,s3})$	-2.793	3.115	0.370	0.037	0.090	0.680
**FEF2575**						
$\log(BMI_{s1s2}) \rightarrow \log(FEF2575_{s2,s3})$	-2.770	2.721	0.310	0.066	0.092	0.470
$\log(BMI_{s1s2,s3}) \rightarrow \log(FEF2575_{s1,s2,s3})$	-2.345	2.992	0.430	0.029	0.086	0.740

[1] β_{c1}: causal BMI main effect per one BMI-increasing allele; β_{c2}: causal BMI*Age interaction effect per one BMI-increasing allele.

The negative sign of the causal main effect means that, keeping all other predictors fixed, at age 18 (which has been chosen as the origin in our analysis) BMI has a causal negative effect on LF. The positive sign of the Age:BMI causal interactive effect implies that, as age increases, the detrimental effect of BMI on LF decreases. As a consequence, the total effect of BMI becomes null at middle ages and protective at older ages.

Supplement Table 9. Causal effects[1] of BMI on FEF2575/FVC in predictive and in long-term cross-sectional models.

**Speliotes (2010) BMI genetic score**				
	N	β_{c1}, β_{c2}	SE	p-value
**FEF2575/FVC**				
$\log(BMI_{s1s2}) \rightarrow FEF2575/FVC_{s2,s3}$	2468	-1.501	0.762	0.049
$\log(BMI_{s1s2}):AGE \rightarrow FEF2575/FVC_{s2,s3}$		0.049	0.031	0.118
$\log(BMI_{s1s2,s3}) \rightarrow FEF2575/FVC_{s1,s2,s3}$	2365	-2.038	0.966	0.035
$\log(BMI_{s1s2,s3}):AGE \rightarrow FEF2575/FVC_{s1,s2,s3}$		0.053	0.032	0.095
**Yengo (2018) BMI genetic score**				
	N	β_{c1}, β_{c2}	SE	p-value
**FEF2575/FVC**				
$\log(BMI_{s1s2}) \rightarrow FEF2575/FVC_{s2,s3}$	2468	-0.358	0.354	0.312
$\log(BMI_{s1s2}):AGE \rightarrow FEF2575/FVC_{s2,s3}$		0.011	0.012	0.355
$\log(BMI_{s1s2,s3}) \rightarrow FEF2575/FVC_{s1,s2,s3}$	2365	-0.475	0.414	0.251
$\log(BMI_{s1s2,s3}):AGE \rightarrow FEF2575/FVC_{s1,s2,s3}$		0.010	0.012	0.439
**Felix (2016) BMI genetic score**				
	N	β_{c1}, β_{c2}	SE	p-value
**FEF2575/FVC**				
$\log(BMI_{s1s2}) \rightarrow FEF2575/FVC_{s2,s3}$	2468	-2.378	1.362	0.081
$\log(BMI_{s1s2}):AGE \rightarrow FEF2575/FVC_{s2,s3}$		0.062	0.048	0.189
$\log(BMI_{s1s2,s3}) \rightarrow FEF2575/FVC_{s1,s2,s3}$	2365	-2.173	1.721	0.207
$\log(BMI_{s1s2,s3}):AGE \rightarrow FEF2575/FVC_{s1,s2,s3}$		0.035	0.051	0.494

[1] β_{c1}: causal BMI main effect per one BMI-increasing allele; β_{c2}: causal BMI*Age interaction effect per one BMI-increasing allele.

The negative sign of the causal main effect means that, keeping all other predictors fixed, at age 18 (which has been chosen as the origin in our analysis) BMI has a causal negative effect on LF. The positive sign of the Age:BMI causal interactive effect implies that, as age increases, the detrimental effect of BMI on LF decreases. As a consequence, the total effect of BMI becomes null at middle ages and protective at older ages.

Supplement Table 10. Causal effects[1] of BMI on FEV1/FVC and FEF2575 in predictive and in long-term cross-sectional models: sensitivity analysis restricted to non-asthmatics.

**Speliotes (2010) BMI genetic score**				
	N	β_{c1}, β_{c2}	SE	p-value
**FEV1/FVC**				
$\log(BMI_{s1s2}) \rightarrow FEV1/FVC_{s2,s3}$	2470	-0.519	0.271	0.056
$\log(BMI_{s1s2}):AGE \rightarrow FEV1/FVC_{s2,s3}$		0.017	0.011	0.131
$\log(BMI_{s1s2s3}) \rightarrow FEV1/FVC_{s1,s2,s3}$	2367	-0.799	0.365	0.028
$\log(BMI_{s1s2s3}):Age \rightarrow FEV1/FVC_{s1,s2,s3}$		0.021	0.012	0.077
**FEF2575**				
$\log(BMI_{s1s2}) \rightarrow FEF2575_{s2,s3}$	2468	-5.833	3.714	0.116
$\log(BMI_{s1s2}):AGE \rightarrow FEF2575_{s2,s3}$		0.169	0.156	0.278
$\log(BMI_{s1s2,s3}) \rightarrow FEF2575_{s1,s2,s3}$	2365	-9.431	5.275	0.074
$\log(BMI_{s1s2,s3}):AGE \rightarrow FEF2575_{s1,s2,s3}$		0.220	0.170	0.194
**Yengo (2018) BMI genetic score**				
	N	β_{c1}, β_{c2}	SE	p-value
**FEV1/FVC**				
$\log(BMI_{s1s2}) \rightarrow FEV1/FVC_{s2,s3}$	2470	-0.181	0.132	0.171
$\log(BMI_{s1s2}):AGE \rightarrow FEV1/FVC_{s2,s3}$		0.006	0.005	0.200
$\log(BMI_{s1s2s3}) \rightarrow FEV1/FVC_{s1,s2,s3}$	2367	-0.342	0.151	0.024
$\log(BMI_{s1s2s3}):Age \rightarrow FEV1/FVC_{s1,s2,s3}$		0.008	0.004	0.055
**FEF2575**				
$\log(BMI_{s1s2}) \rightarrow FEF2575_{s2,s3}$	2468	-2.949	1.837	0.109
$\log(BMI_{s1s2}):AGE \rightarrow FEF2575_{s2,s3}$		0.094	0.064	0.141
$\log(BMI_{s1s2,s3}) \rightarrow FEF2575_{s1,s2,s3}$	2365	-4.616	2.224	0.038
$\log(BMI_{s1s2,s3}):AGE \rightarrow FEF2575_{s1,s2,s3}$		0.117	0.066	0.075

Felix (2016) BMI genetic score				
	N	β_{c1}, β_{c2}	SE	p-value
**FEV1/FVC**				
$\log(BMI_{s1s2}) \rightarrow FEV1/FVC_{s2,s3}$	2470	-0.620	0.400	0.121
$\log(BMI_{s1s2}):AGE \rightarrow FEV1/FVC_{s2,s3}$		0.017	0.014	0.213
$\log(BMI_{s1s2s3}) \rightarrow FEV1/FVC_{s1,s2,s3}$	2367	-0.605	0.478	0.206
$\log(BMI_{s1s2s3}):Age \rightarrow FEV1/FVC_{s1,s2,s3}$		0.009	0.015	0.516
**FEF2575**				
$\log(BMI_{s1s2}) \rightarrow FEF2575_{s2,s3}$	2468	-10.132	5.836	0.083
$\log(BMI_{s1s2}):AGE \rightarrow FEF2575_{s2,s3}$		0.288	0.204	0.160
$\log(BMI_{s1s2,s3}) \rightarrow FEF2575_{s1,s2,s3}$	2365	-9.023	7.072	0.202
$\log(BMI_{s1s2,s3}):AGE \rightarrow FEF2575_{s1,s2,s3}$		0.147	0.216	0.495

[1] β_{c1}: causal BMI main effect per one BMI-increasing allele; β_{c2}: causal BMI*Age interaction effect per one BMI-increasing allele.

The negative sign of the causal main effect means that, keeping all other predictors fixed, at age 18 (which has been chosen as the origin in our analysis) BMI has a causal negative effect on LF. The positive sign of the Age:BMI causal interactive effect implies that, as age increases, the detrimental effect of BMI on LF decreases. As a consequence, the total effect of BMI becomes null at middle ages and protective at older ages.

Supplement Table 11. Causal effects[1] of BMI on FEV1/FVC and FEF2575 in predictive and in long-term cross-sectional models. Childhood BMI genetic score: (5)32 SNPs), sensitivity analysis with additional adjustment for study area and educational level

	N	β_{c1}, β_{c2}	SE	p-value
**FEV1/FVC**				
**Predictive model**				
BMI main effect<br>log(BMI_{s1s2})→$FEV1/FVC_{s2,s3}$	2853	-0.557	0.254	0.028
BMI*Age interaction effect<br>log(BMI_{s1s2}): Age_{s1s2}→$FEV1/FVC_{s2,s3}$		0.019	0.010	0.064
**Long-term cross-sectional model**				
BMI main effect<br>log(BMI_{s1s2s3})→$FEV1/FVC_{s1,s2,s3}$	2731	-0.750	0.312	0.016
BMI*Age interaction effect<br>log(BMI_{s1s2s3}): $Age_{s1,s2,s3}$→$FEV1/FVC_{s1,s2,s3}$		0.021	0.010	0.039
**FEF2575**				
**Predictive model**				
BMI main effect<br>log(BMI_{s1s2})→$FEF2575_{s2,s3}$	2850	-7.125	3.440	0.038
BMI*Age interaction effect<br>log(BMI_{s1s2}):Age_{s1s2} →$FEF2575_{s2,s3}$		0.221	0.140	0.114
**Long-term cross-sectional model**				
BMI main effect<br>log($BMI_{s1s2,s3}$) →$FEF2575_{s1,s2,s3}$	2728	-9.262	4.412	0.035
BMI*Age interaction effect<br>log($BMI_{s1,s2,s3}$):$Age_{s1,s2,s3}$ →$FEF2575_{s1,s2,s3}$		0.243	0.144	0.092

[1] β_{c1}: causal BMI main effect per one BMI-increasing allele; β_{c2}: causal BMI*Age interaction effect per one BMI-increasing allele.

The negative sign of the causal main effect means that, keeping all other predictors fixed, at age 18 (which has been chosen as the origin in our analysis) BMI has a causal negative effect on LF. The positive sign of the Age:BMI causal interactive effect implies that, as age increases, the detrimental effect of BMI on LF decreases. As a consequence, the total effect of BMI becomes null at middle ages and protective at older ages.

Supplement Table 12: Observational associations of BMI with FEV1/FVC and FEF2575 in predictive and in long-term cross-sectional models, sensitivity analysis using Inverse Probability Weighting

(a) with weights computed predicting participation in SAP2-SAP3 based on SAP1

	N	β_1, β_2[1]	SE	p-value
**FEV1/FVC**				
**Predictive model**				
BMI main effect<br>$\log(BMI_{s1s2}) \rightarrow FEV1/FVC_{s2,s3}$	2853	-0.012	0.025	0.638
BMI*Age interaction effect<br>$\log(BMI_{s1s2})$: $Age_{s1s2} \rightarrow FEV1/FVC_{s2,s3}$		0.0007	0.0008	0.396
**Long-term cross-sectional model**				
BMI main effect<br>$\log(BMI_{s1s2s3}) \rightarrow FEV1/FVC_{s1,s2,s3}$	2731	-0.004	0.0026	0.096
BMI*Age interaction effect<br>$\log(BMI_{s1s2s3})$: $Age_{s1,s2,s3} \rightarrow FEV1/FVC_{s1,s2,s3}$		0.0009	0.0007	0.213
**FEF2575**				
**Predictive model**				
BMI main effect<br>$\log(BMI_{s1s2}) \rightarrow FEF2575_{s2,s3}$	2850	0.634	0.336	0.059
BMI*Age interaction effect<br>$\log(BMI_{s1s2})$:$Age_{s1s2} \rightarrow FEF2575_{s2,s3}$		-0.018	0.011	0.100
**Long-term cross-sectional model**				
BMI main effect<br>$\log(BMI_{s1s2,s3}) \rightarrow FEF2575_{s1,s2,s3}$	2728	0.525	0.374	0.184
BMI*Age interaction effect<br>$\log(BMI_{s1,s2,s3})$:$Age_{s1,s2,s3} \rightarrow FEF2575_{s1,s2,s3}$		-0.016	0.011	0.122

[1] β_1: associational BMI main effect; β_2: associational BMI*Age interaction effect

(b) with weights computed predicting participation in SAP3 based on SAP1-SAP2

	N	β_1, β_2[1]	SE	p-value
**FEV1/FVC**				
**Predictive model**				
<u>BMI main effect</u><br>$\log(BMI_{s1s2}) \rightarrow FEV1/FVC_{s2,s3}$	2853	-0.012	0.025	0.626
<u>BMI*Age interaction effect</u><br>$\log(BMI_{s1s2})$: $Age_{s1s2} \rightarrow FEV1/FVC_{s2,s3}$		0.0007	0.0008	0.385
**Long-term cross-sectional model**				
<u>BMI main effect</u><br>$\log(BMI_{s1s2s3}) \rightarrow FEV1/FVC_{s1,s2,s3}$	2731	-0.004	0.0027	0.085
<u>BMI*Age interaction effect</u><br>$\log(BMI_{s1s2s3})$: $Age_{s1,s2,s3} \rightarrow FEV1/FVC_{s1,s2,s3}$		0.0010	0.0007	0.189
**FEF2575**				
**Predictive model**				
<u>BMI main effect</u><br>$\log(BMI_{s1s2}) \rightarrow FEF2575_{s2,s3}$	2850	0.628	0.336	0.061
<u>BMI*Age interaction effect</u><br>$\log(BMI_{s1s2})$:$Age_{s1s2} \rightarrow FEF2575_{s2,s3}$		-0.018	0.011	0.100
**Long-term cross-sectional model**				
<u>BMI main effect</u><br>$\log(BMI_{s1s2,s3}) \rightarrow FEF2575_{s1,s2,s3}$	2728	0.476	0.375	0.204
<u>BMI*Age interaction effect</u><br>$\log(BMI_{s1,s2,s3})$:$Age_{s1,s2,s3} \rightarrow FEF2575_{s1,s2,s3}$		-0.016	0.010	0.132

[1] β_1: associational BMI main effect; β_2: associational BMI*Age interaction effect

Supplement Table 13. Causal effects[1] of BMI on FEV1/FVC and FEF2575 in predictive and in long-term cross-sectional models, stratified by age at SAP1. Childhood BMI genetic score: (32 SNPS; Speliotes Score)

	Age stratum (at SAP1)	N	β_{c1}	SE	p-value
**FEV1/FVC**					
**Predictive model**					
BMI main effect $\log(BMI_{s1s2}) \rightarrow FEV1/FVC_{s2,s3}$	1st tertile	1094	-0.161	0.093	0.084
	2nd tertile	980	-0.227	0.163	0.163
	3rd tertile	778	0.361	0.272	0.184
**Long-term cross-sectional model**					
BMI main effect $\log(BMI_{s1s2s3}) \rightarrow FEV1/FVC_{s1,s2,s3}$	1st tertile	1035	-0.262	0.113	0.021
	2nd tertile	940	-0.220	0.154	0.154
	3rd tertile	755	0.342	0.232	0.140
**FEF2575**					
**Predictive model**					
BMI main effect $\log(BMI_{s1s2}) \rightarrow FEF2575_{s2,s3}$	1st tertile	1094	-2.696	1.480	0.069
	2nd tertile	978	-2.071	2.047	0.312
	3rd tertile	777	1.698	2.532	0.503
**Long-term cross-sectional model**					
BMI main effect $\log(BMI_{s1s2,s3}) \rightarrow FEF2575_{s1,s2,s3}$	1st tertile	1035	-4.068	1.850	0.028
	2nd tertile	938	-1.553	2.031	0.445
	3rd tertile	754	1.894	2.294	0.409

[1] β_{c1}: causal BMI main effect per one BMI-increasing allele
1st tertile: (18.2, 35.2]; 2nd tertile: (35.2, 46.6]; 3rd tertile: (46.6, 61.7]

Supplement Figure 1. Flowchart of SAPALDIA Baseline and Follow-up Participants: (a) for the prediction model; (b) for the long-term cross-sectional model.

(a)

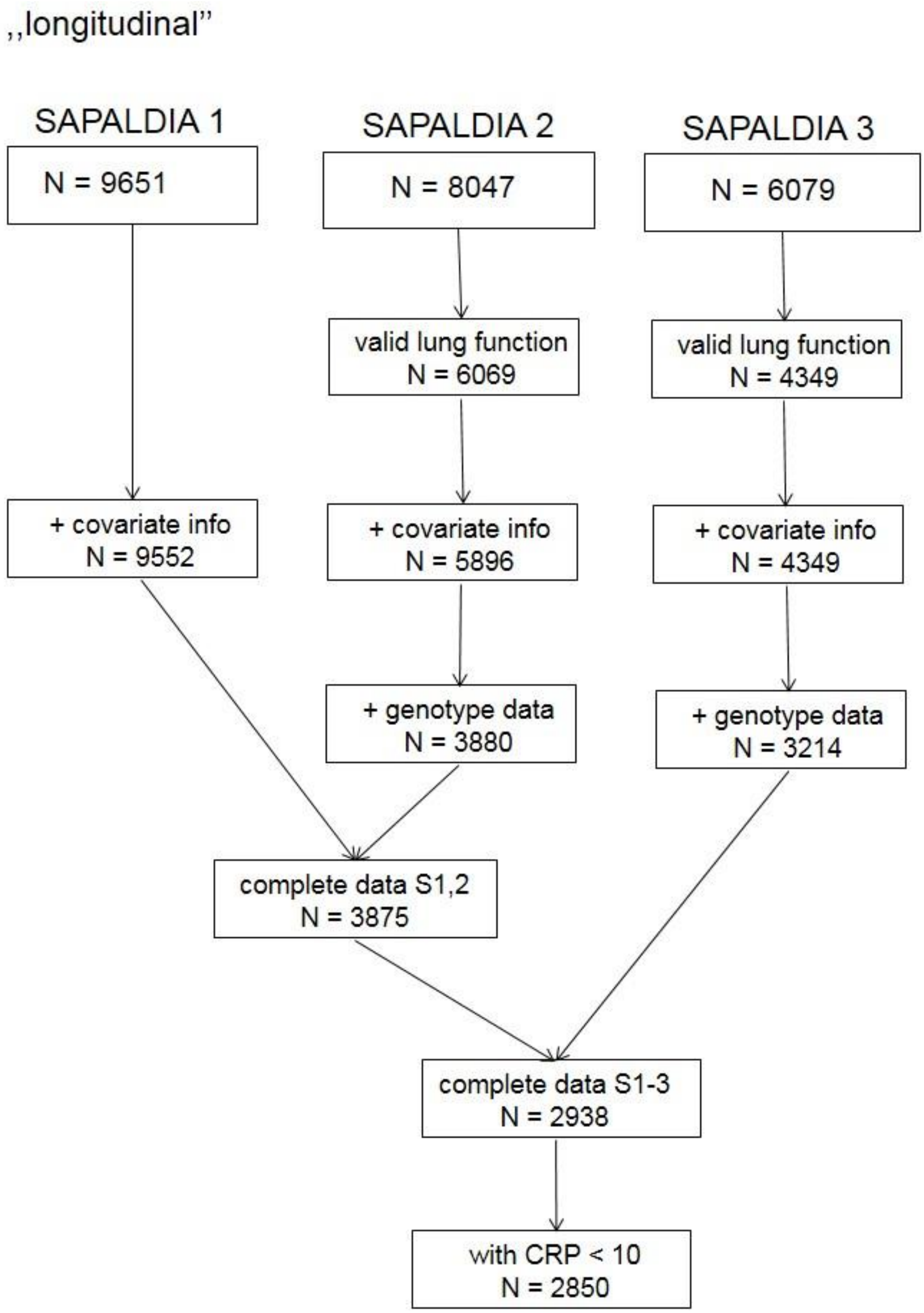

(b)

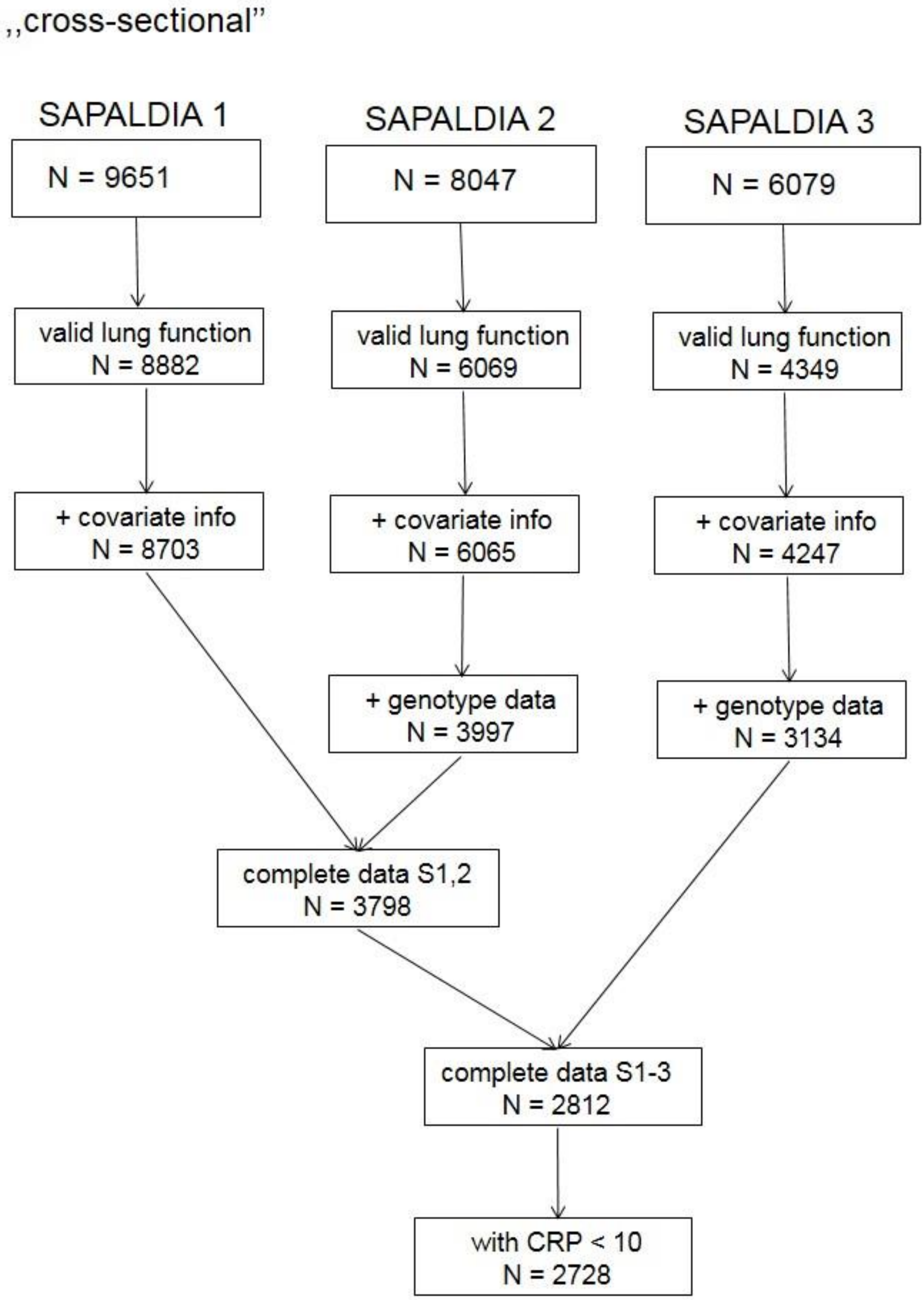
,,cross-sectional"
SAPALDIA 1
N = 9651
SAPALDIA 2
N = 8047
SAPALDIA 3
N = 6079
valid lung function
N = 8882
valid lung function
N = 6069
valid lung function
N = 4349
+ covariate info
N = 8703
+ covariate info
N = 6065
+ covariate info
N = 4247
+ genotype data
N = 3997
+ genotype data
N = 3134
complete data S1,2
N = 3798
complete data S1-3
N = 2812
with CRP < 10
N = 2728

Supplement Figure 2. Distributions of lung function variables at each SAPALDIA survey, by obesity condition (BMI<30 kg/m^2 vs. BMI≥30 kg/m^2). FEV1, FVC

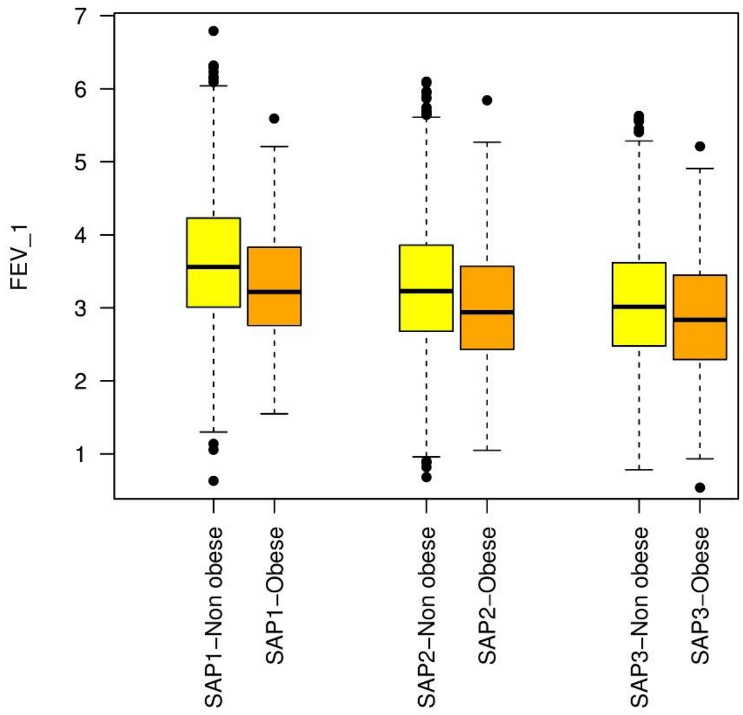

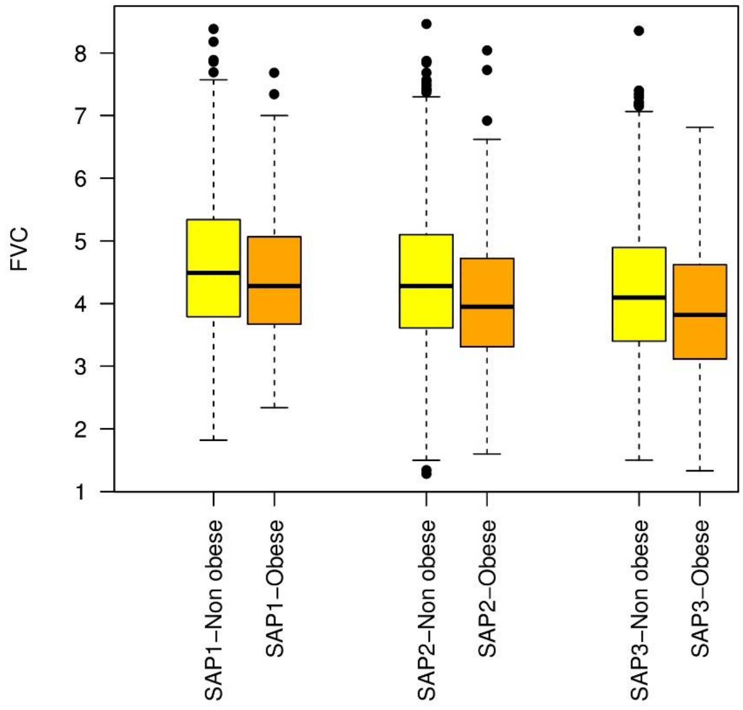

Supplement Figure 3. Histograms of BMI at SAP1, SAP2 and SAP3

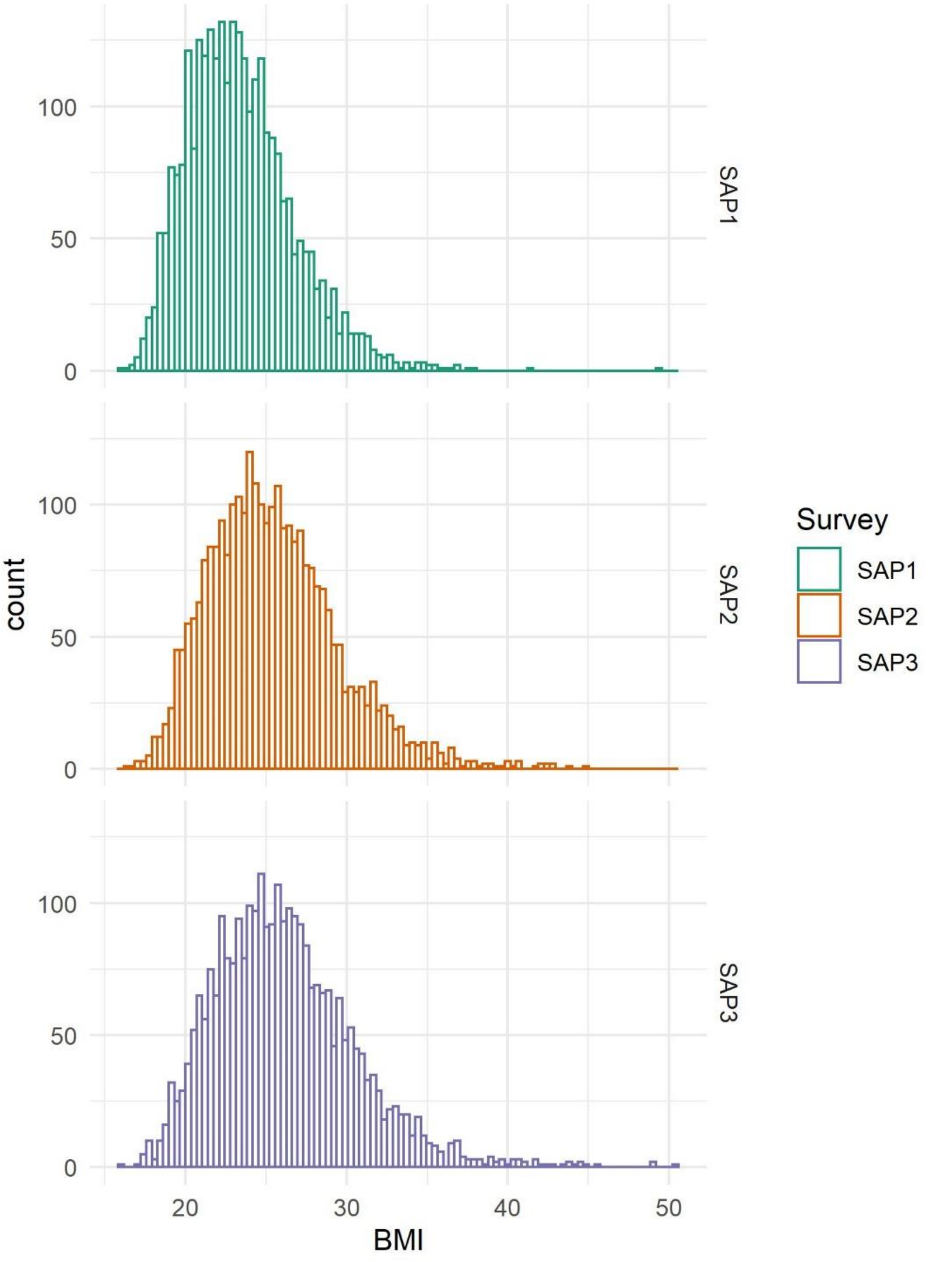

Supplement Figure 4. MR Egger regression results for Speliotes Score

a) FEV1/FVC predictive model, BMI main effect

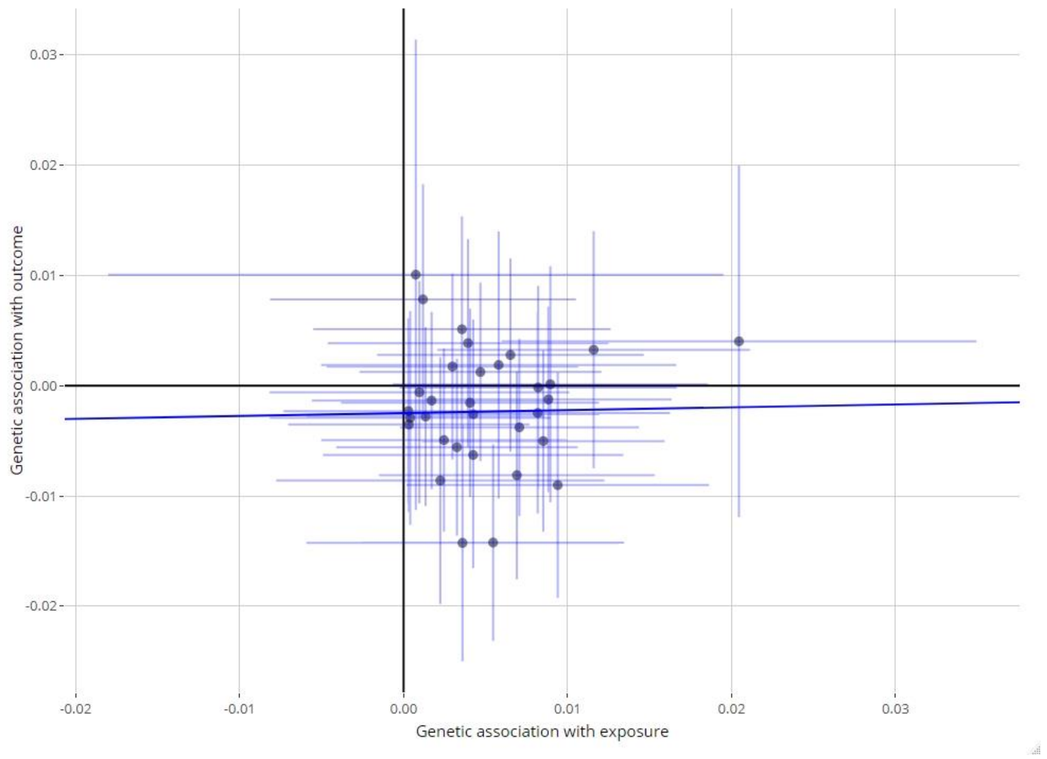

b) FEV/FVC predictive model, BMI:Age interaction effect

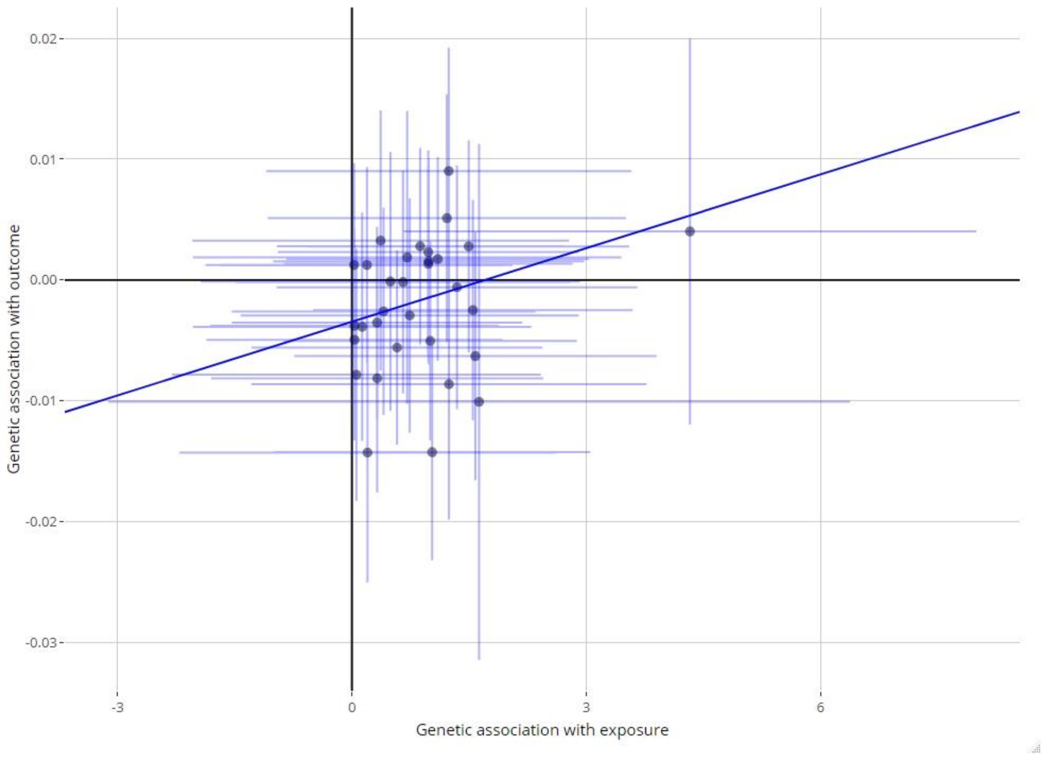

c) FEV1/FVC long-term cross-sectional model, BMI main effect

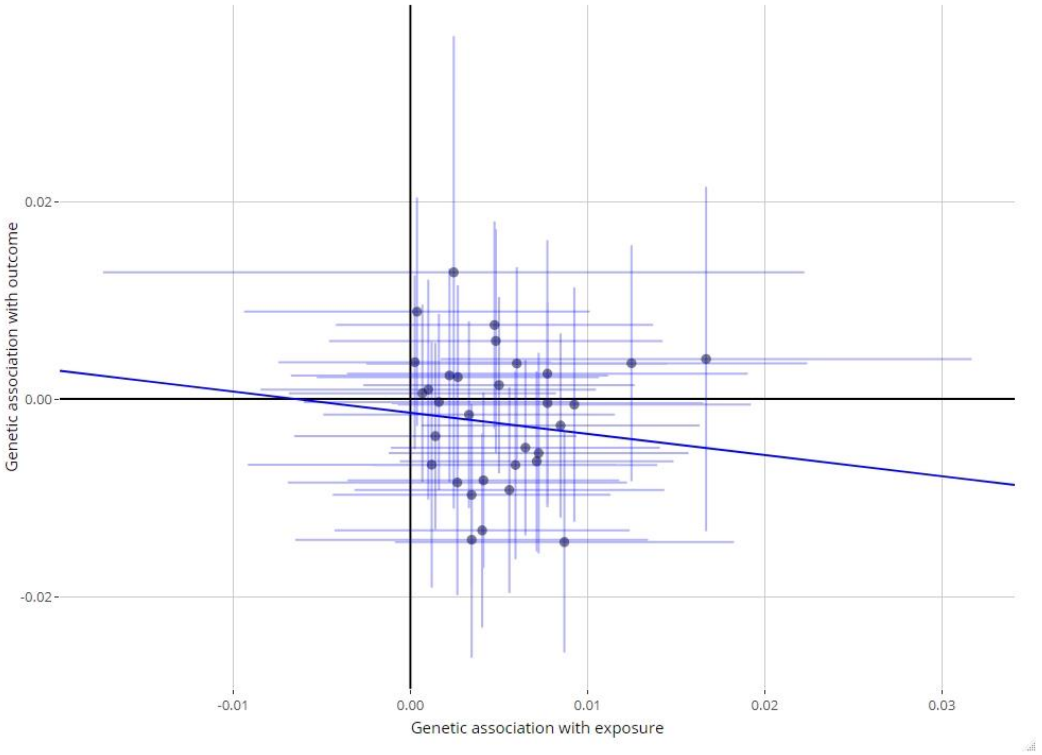

d) FEV/FVC long-term cross-sectional model, BMI:Age interaction effect

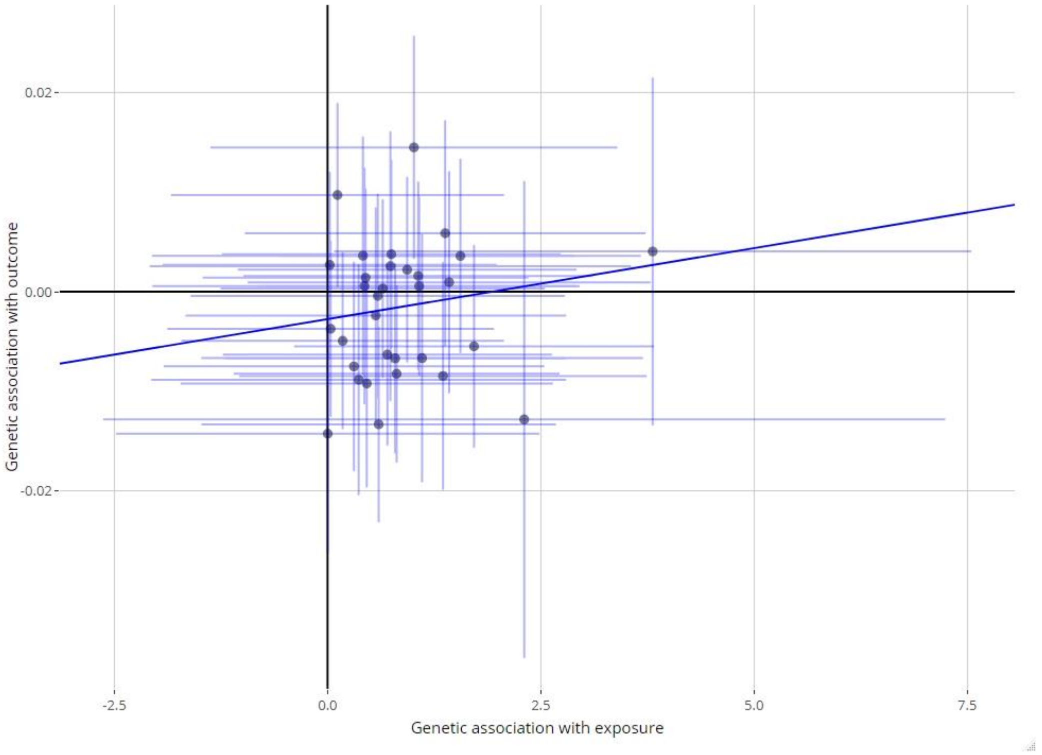

e) FEF2575 predictive model, BMI main effect

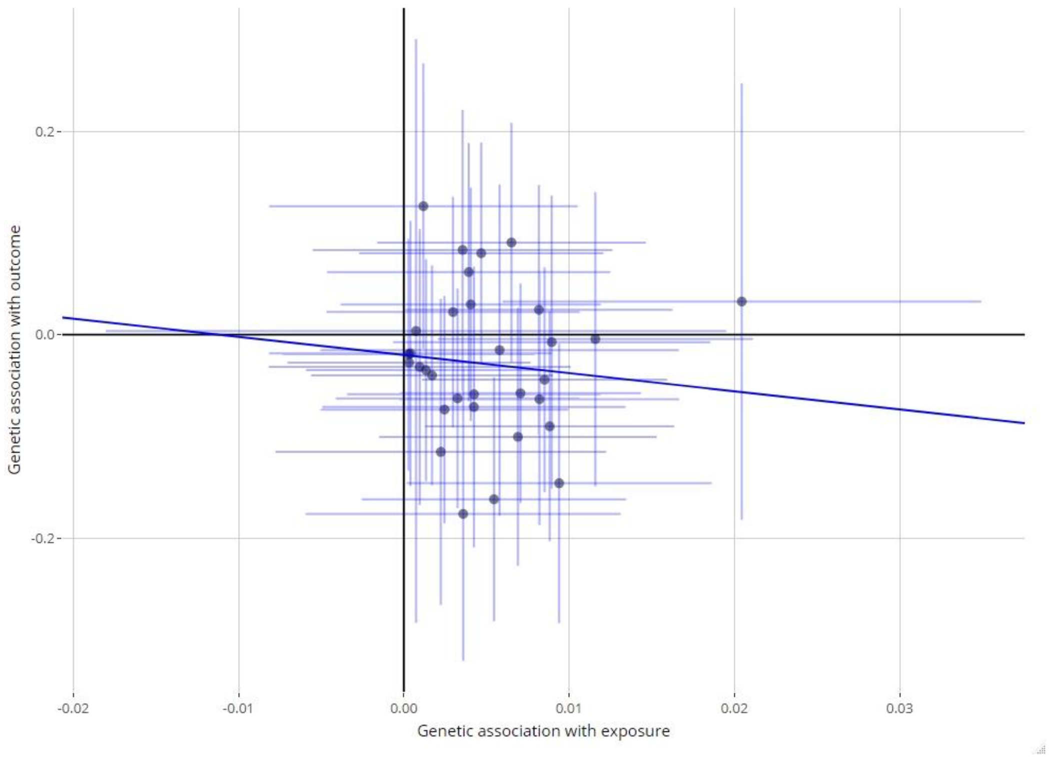

f) FEF2575 predictive model, BMI:Age interaction effect

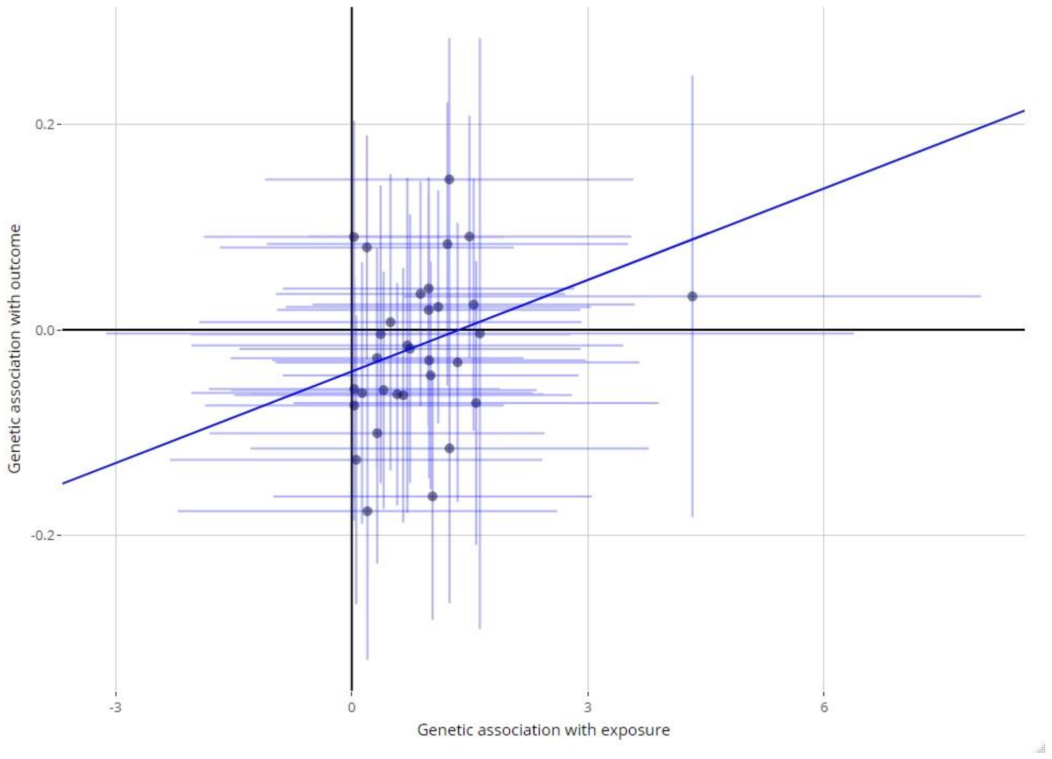

g) FEF2575 long-term cross-sectional model, BMI main effect

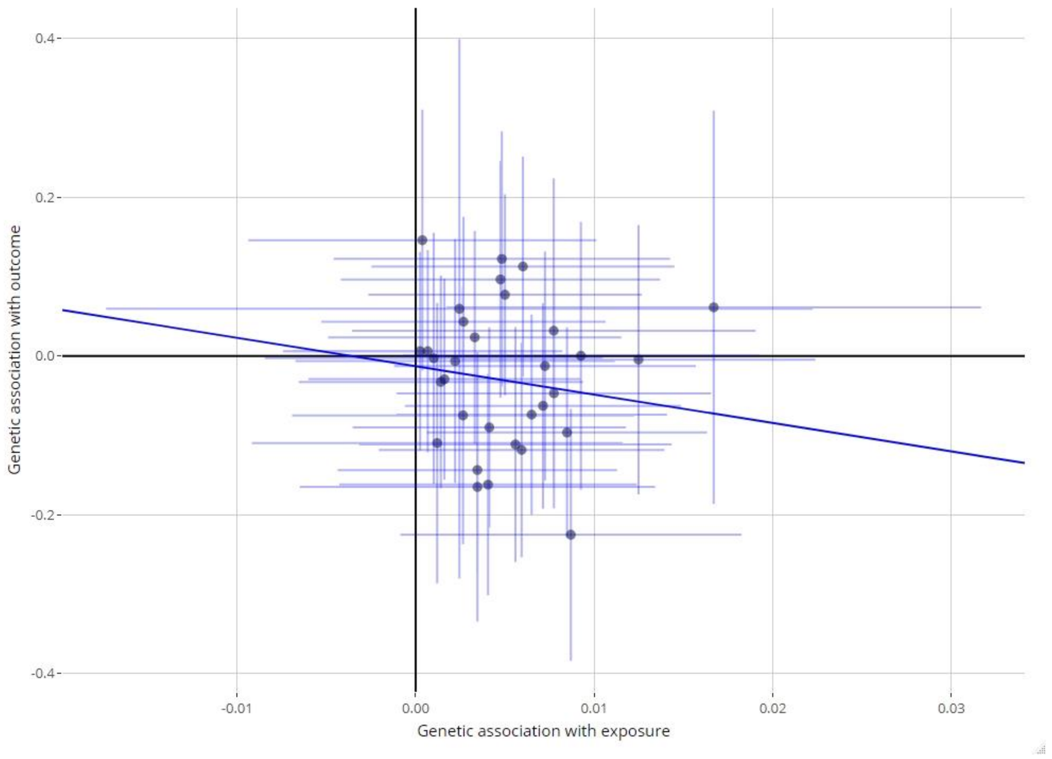

h) FEF2575 long-term cross-sectional model, BMI:Age interaction effect

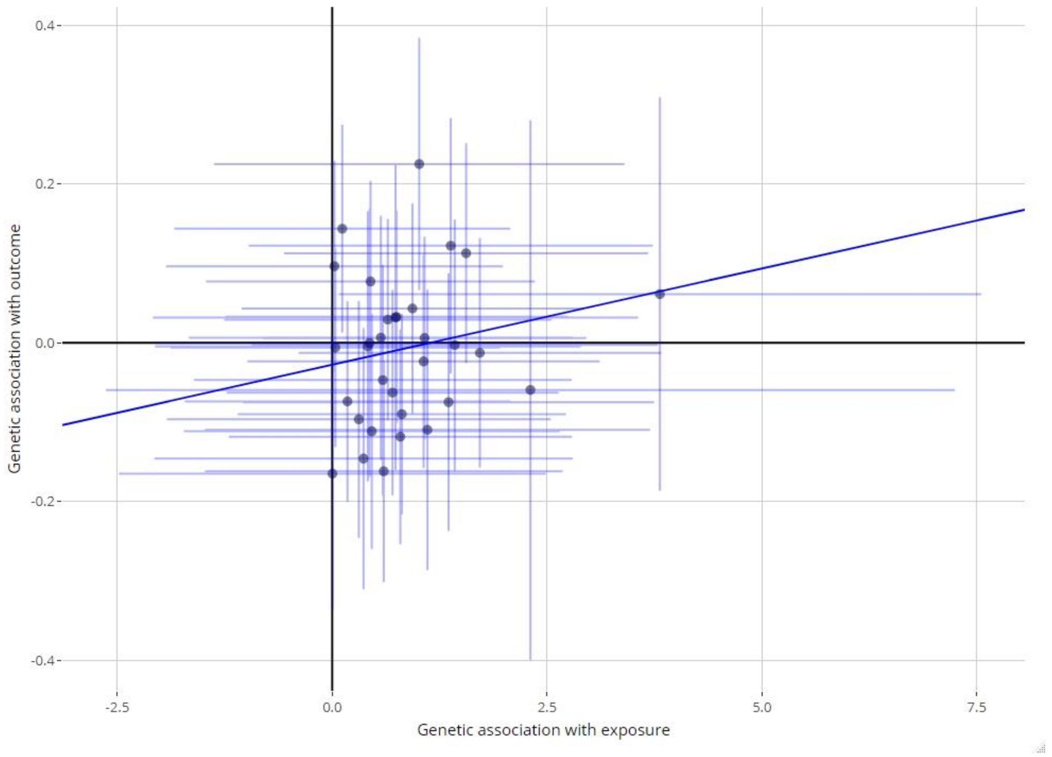

i) FEF2575/FVC predictive model, BMI main effect

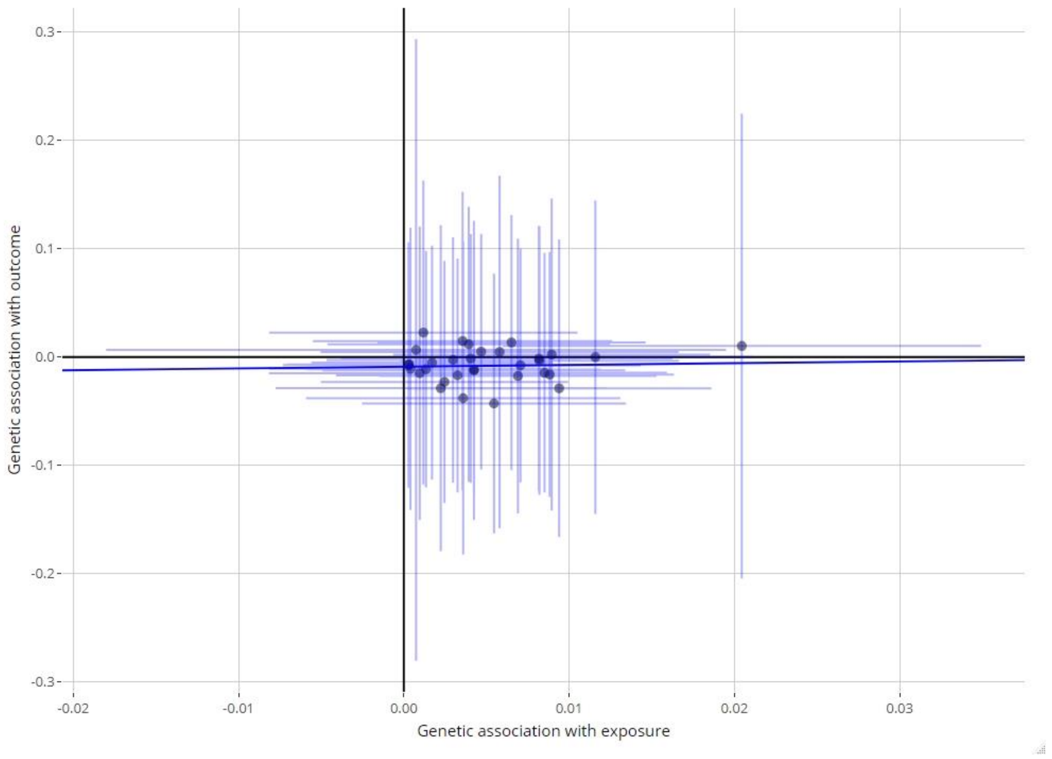

j) FEF2575/FVC predictive model, BMI:Age interaction effect

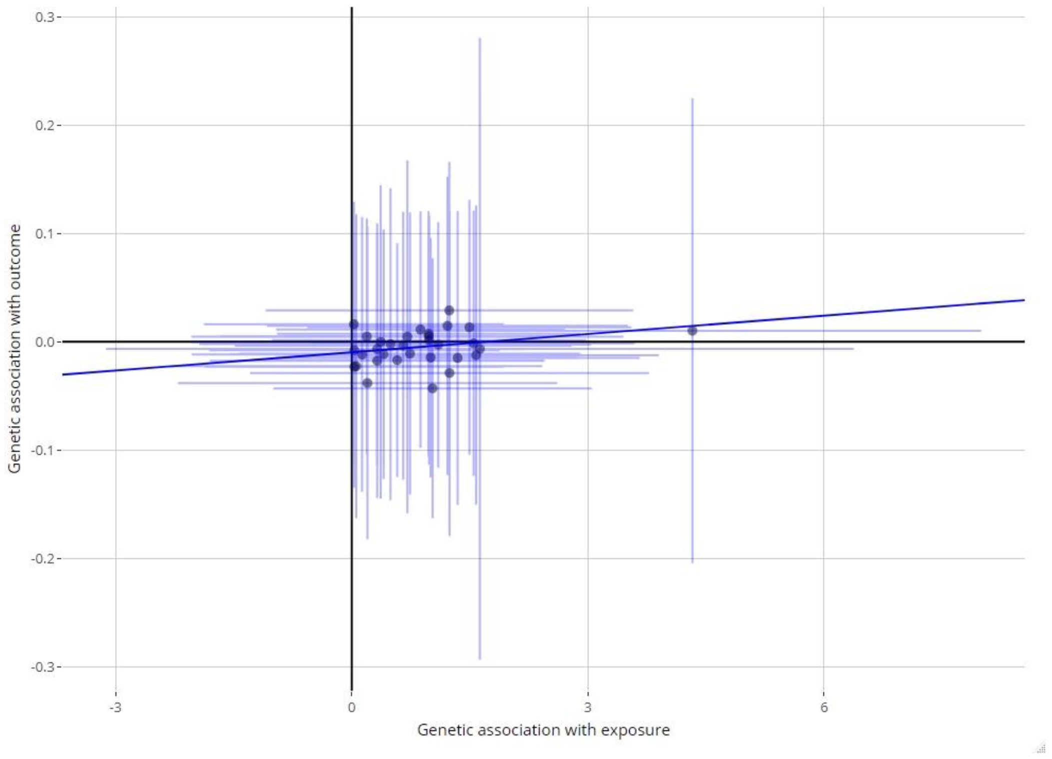

k) FEF2575/FVC long-term cross-sectional model, BMI main effect

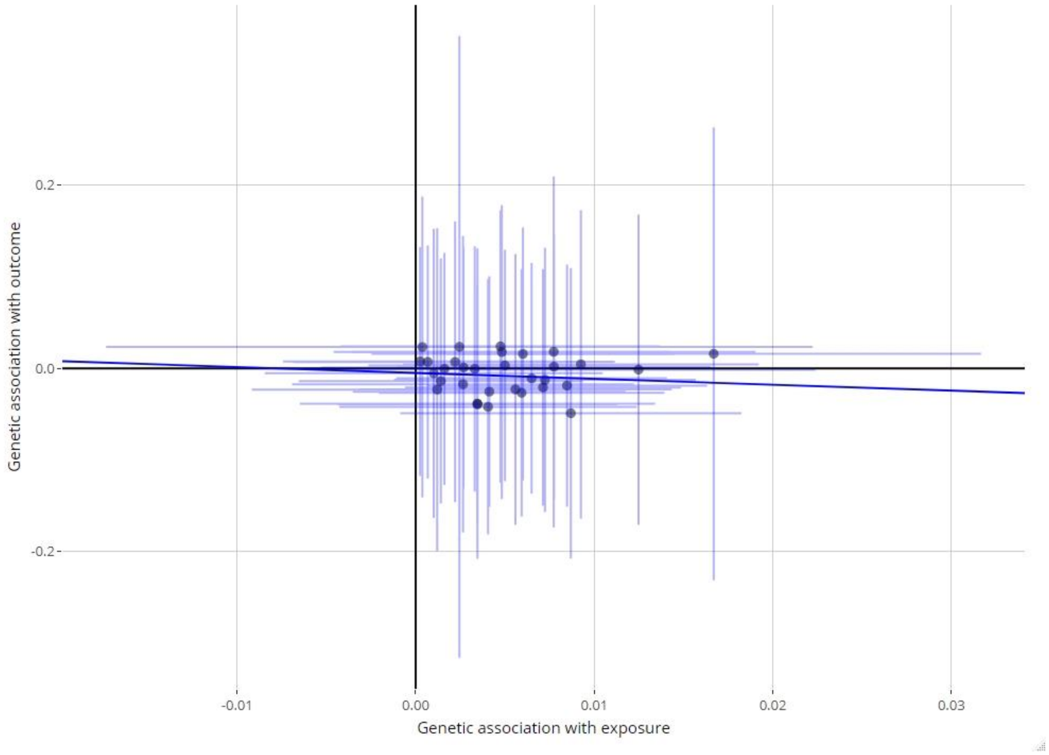

l) FEF2575/FVC long-term cross-sectional model, BMI:Age interaction effect

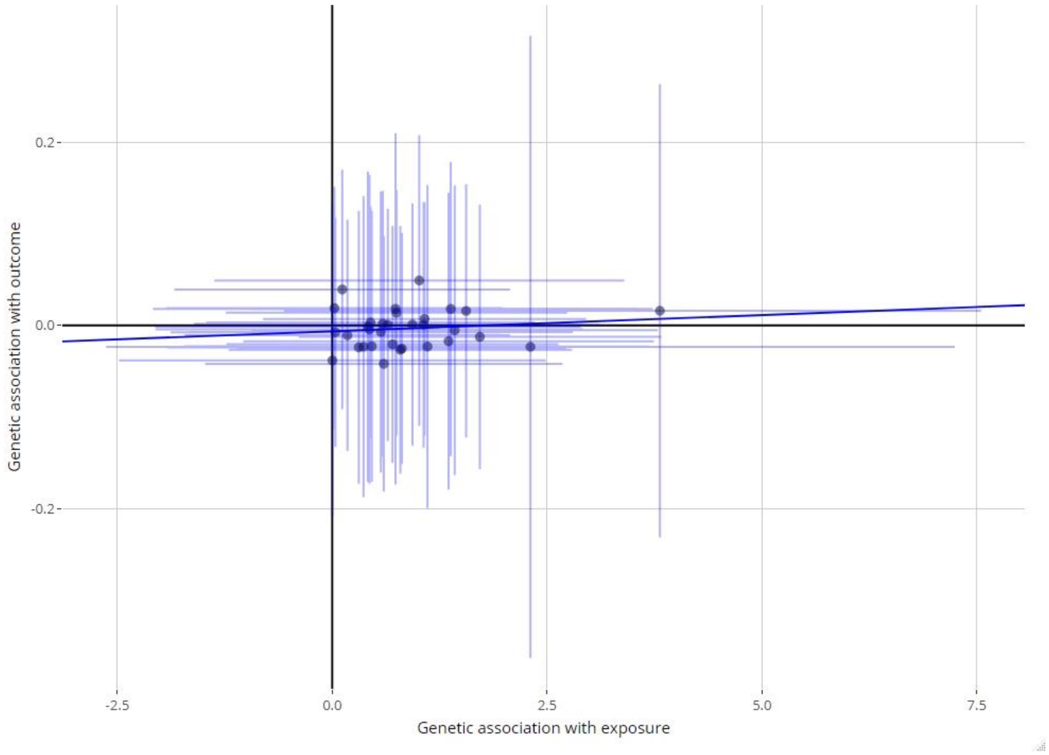

Supplement Figure 5. MR Egger regression results for Yengo Score

a) FEV1/FVC predictive model, BMI main effect

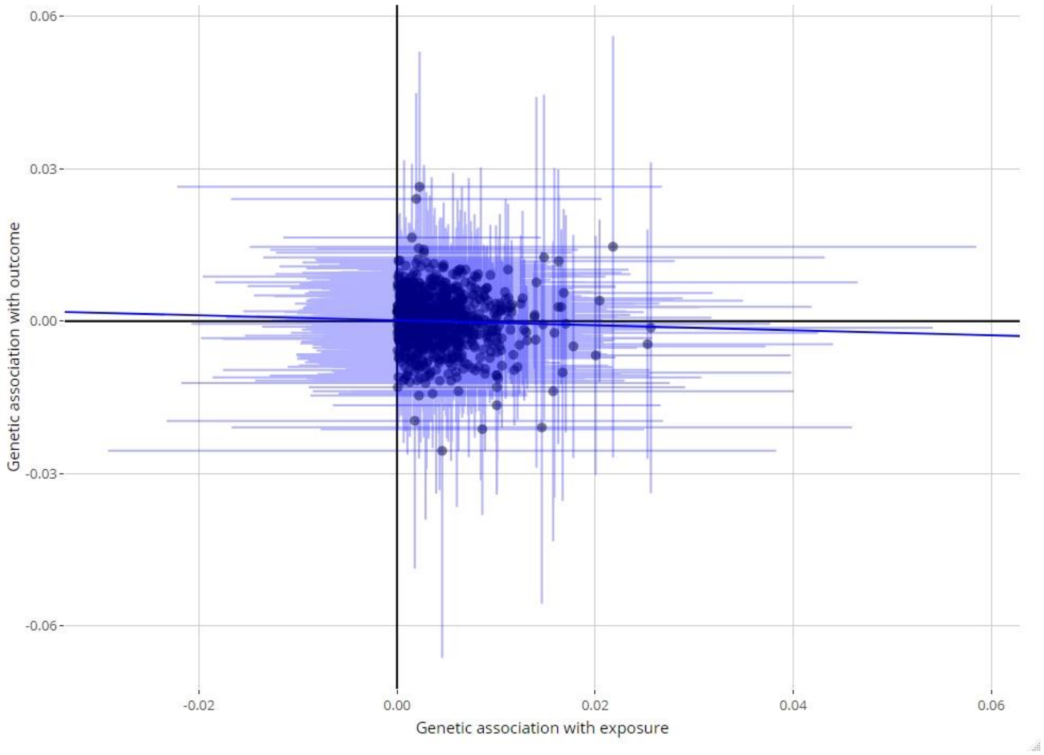

b) FEV/FVC predictive model, BMI:Age interaction effect

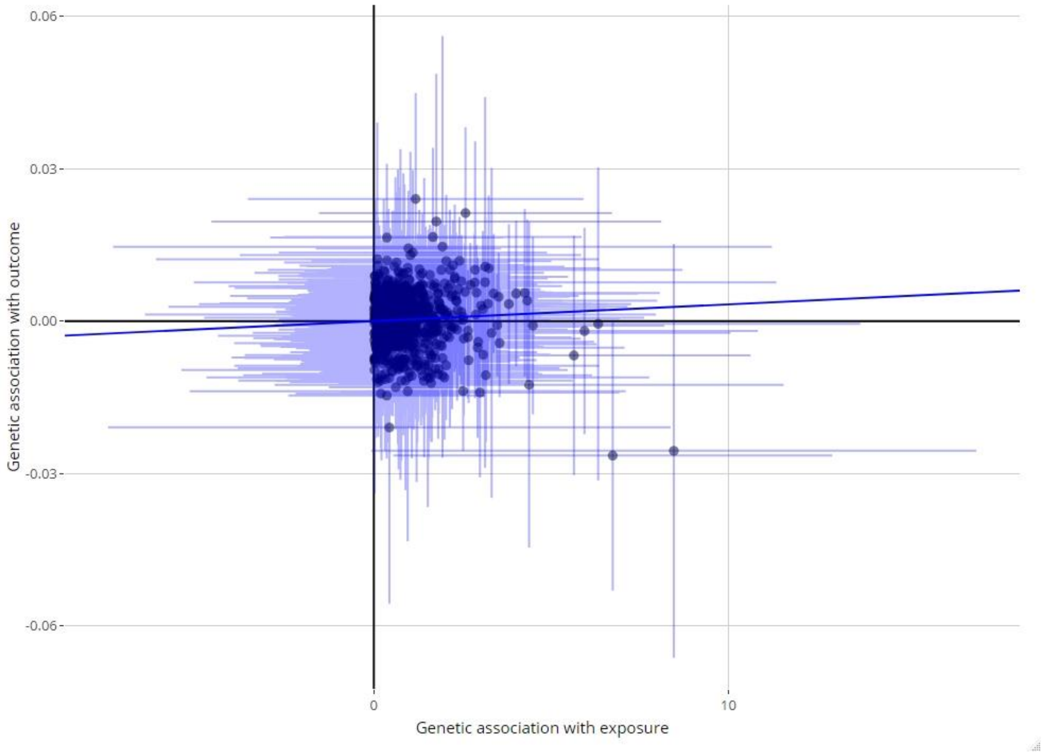

c) FEV1/FVC long-term cross-sectional model, BMI main effect

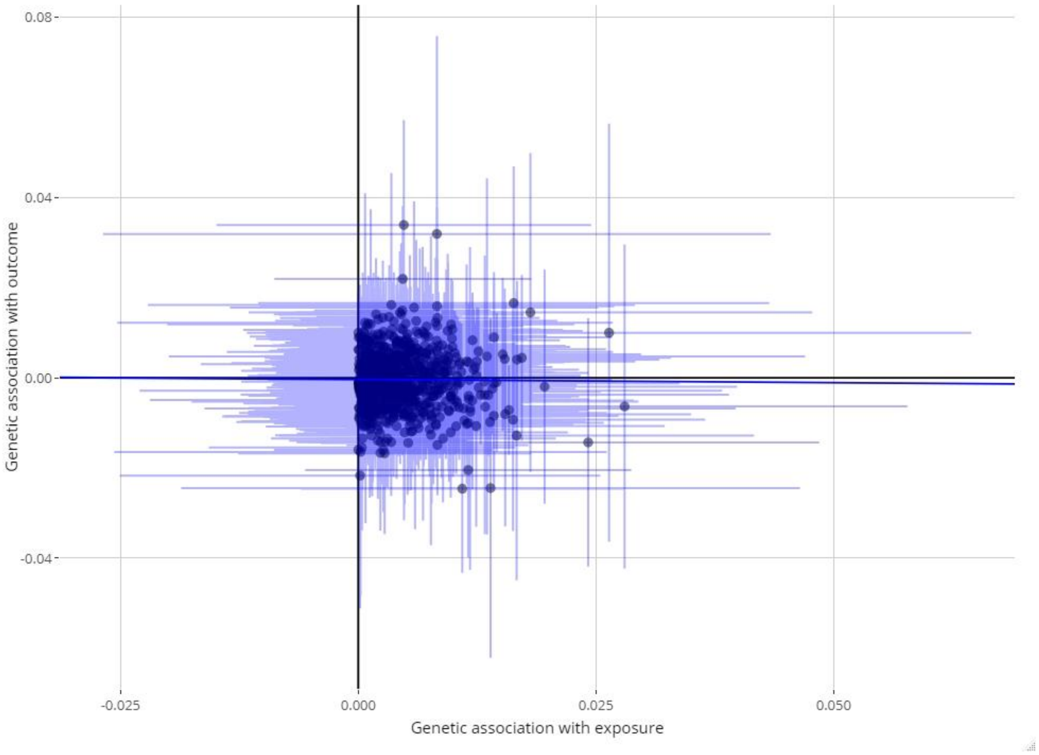

d) FEV/FVC long-term cross-sectional model, BMI:Age interaction effect

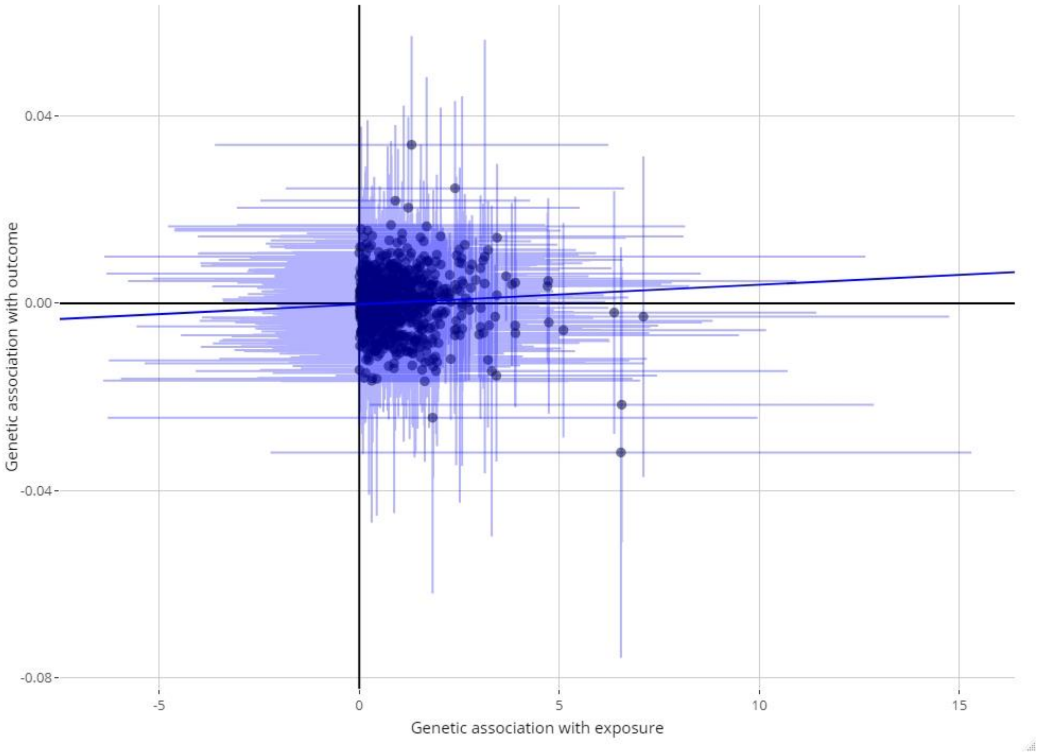

e) FEF2575 predictive model, BMI main effect

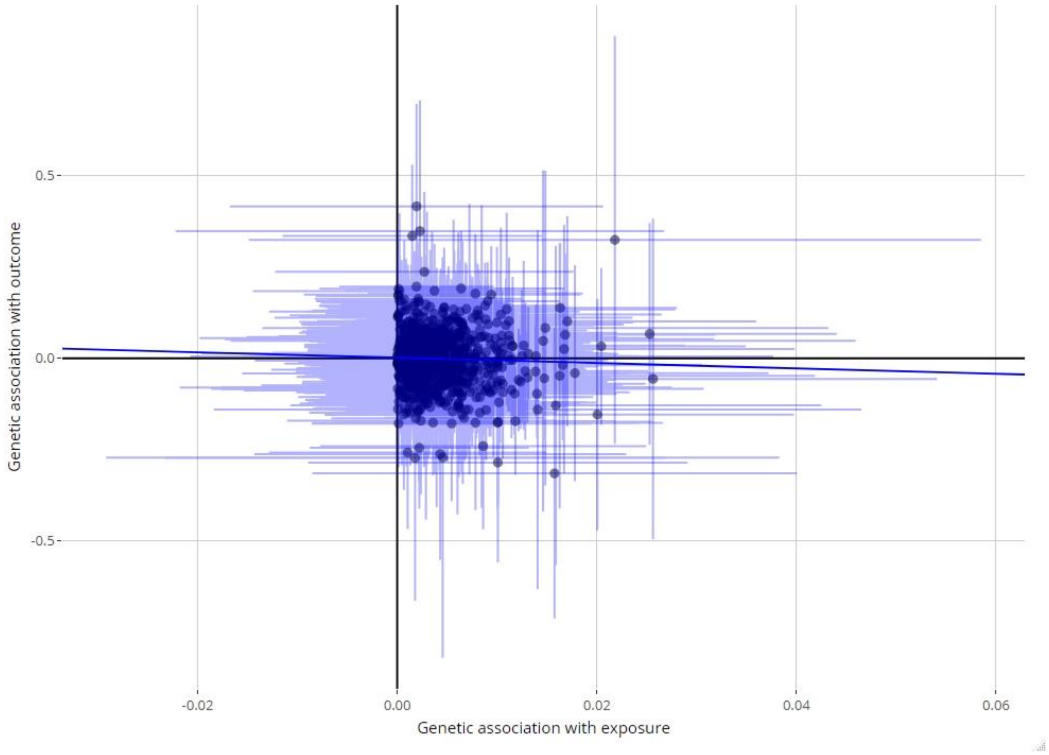

f) FEF2575 predictive model, BMI:Age interaction effect

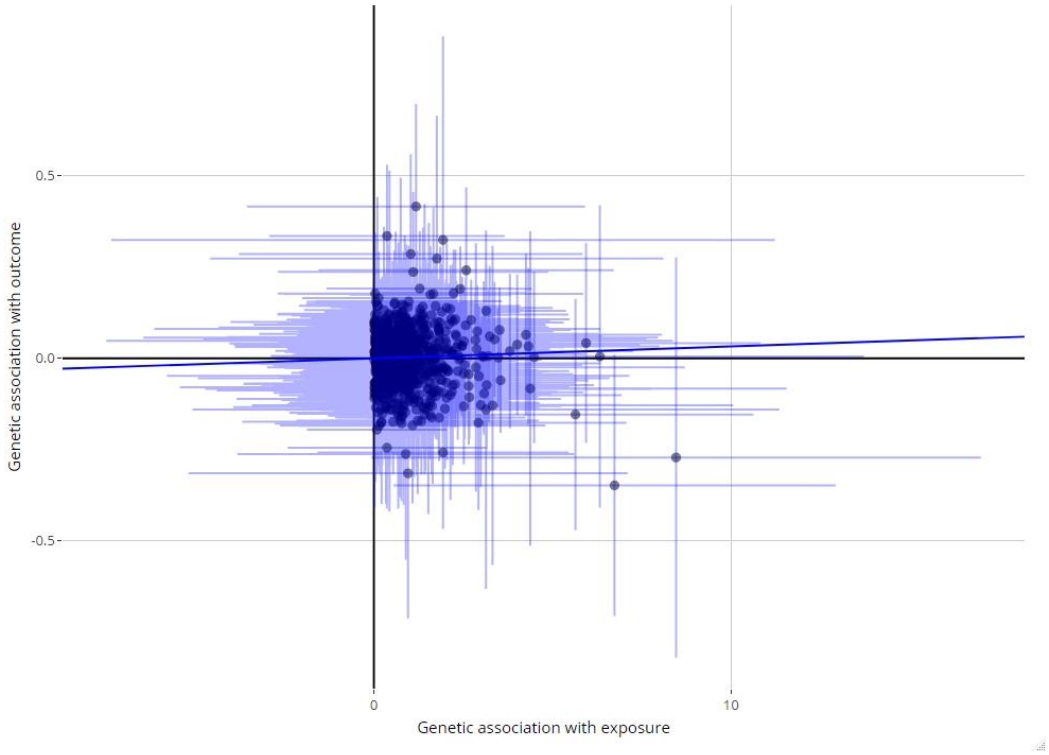

g) FEF2575 long-term cross-sectional model, BMI main effect

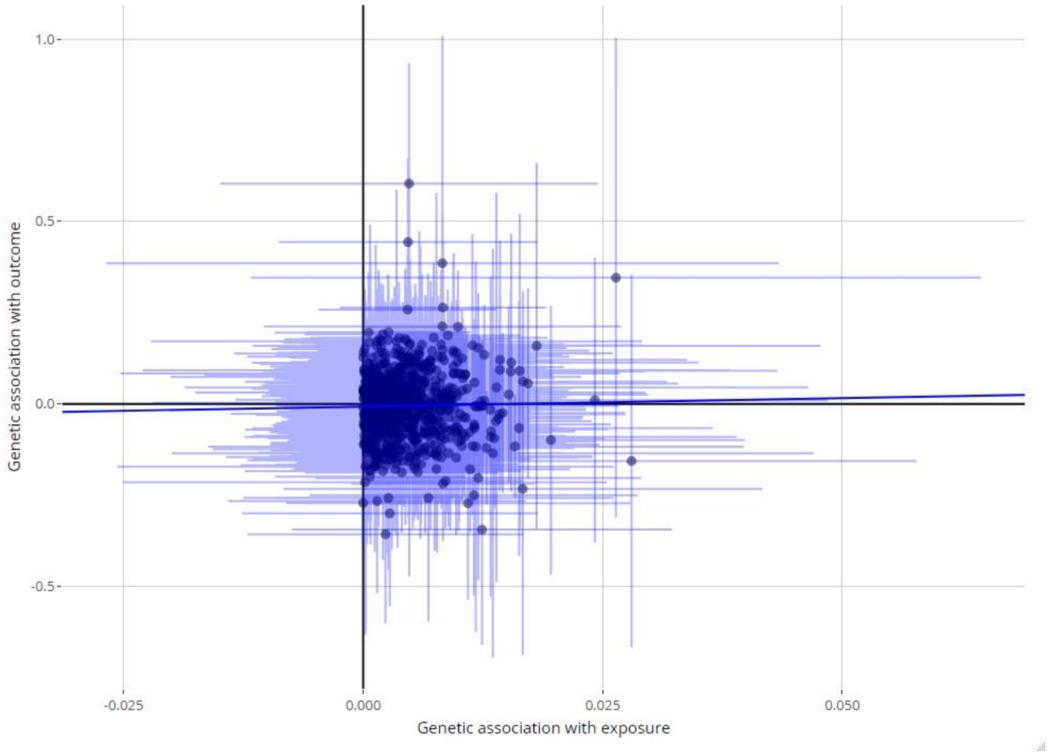

h) FEF2575 long-term cross-sectional model, BMI:Age interaction effect

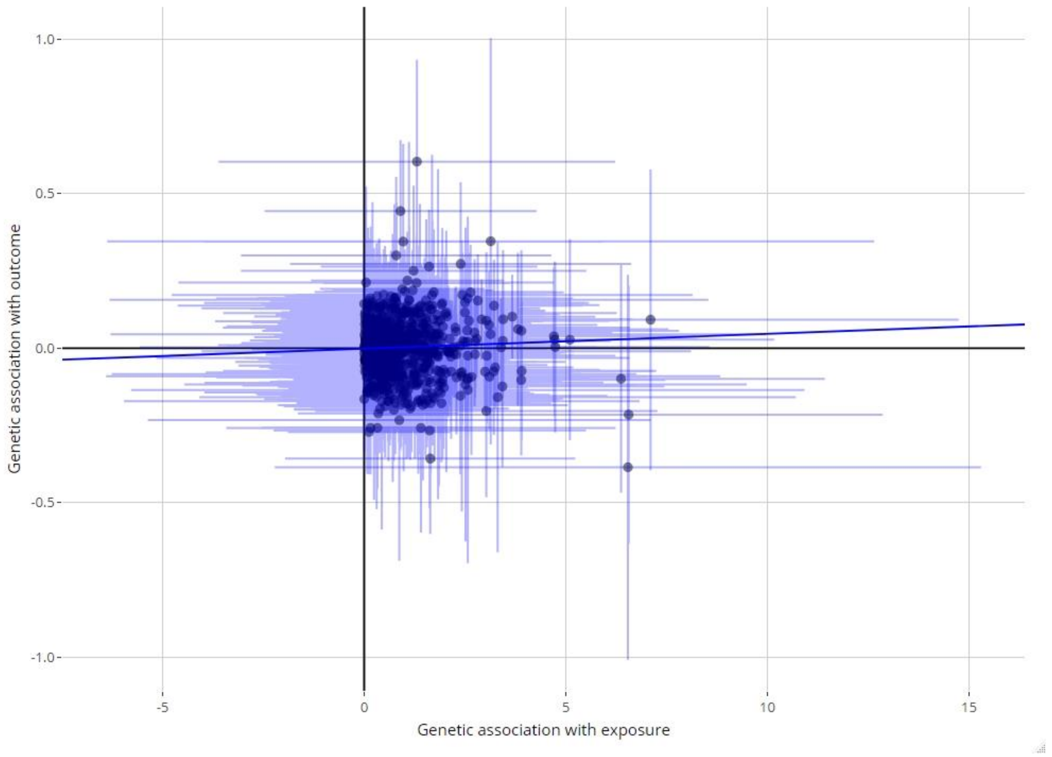

i) FEF2575/FVC predictive model, BMI main effect

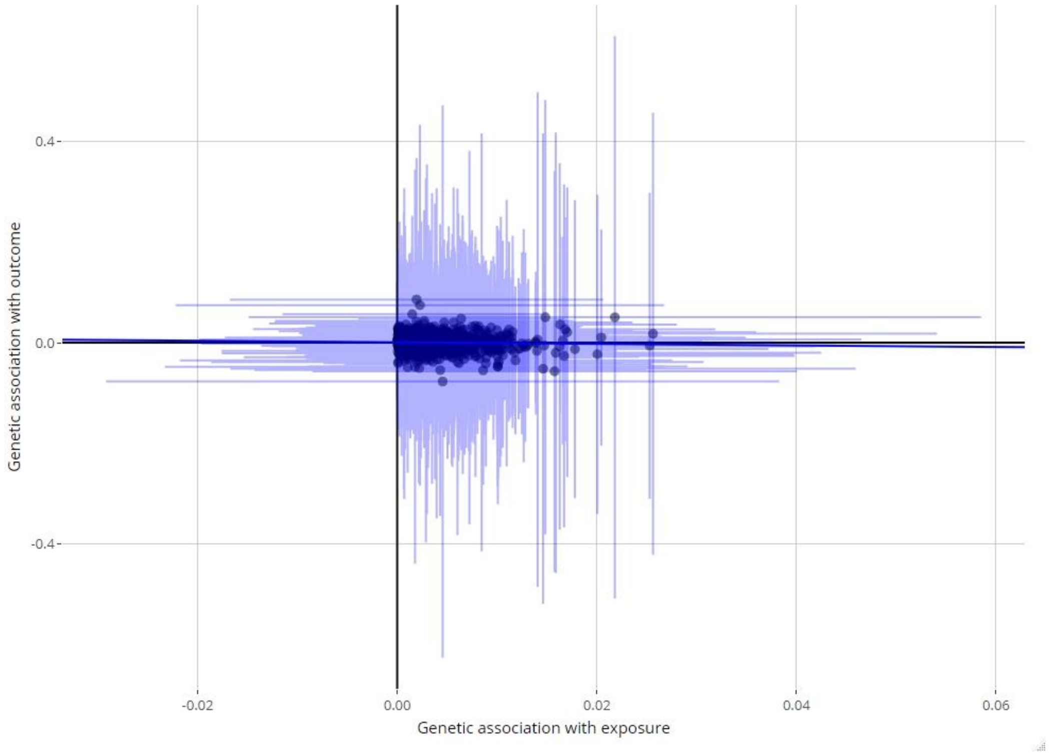

j) FEF2575/FVC predictive model, BMI:Age interaction effect

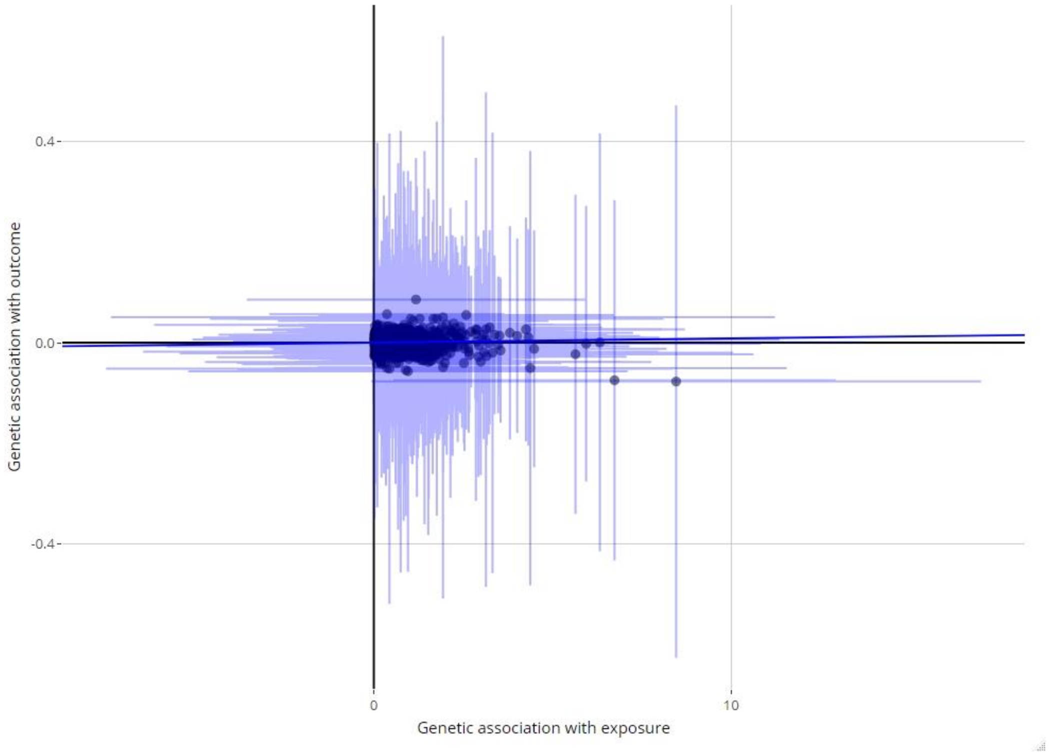

k) FEF2575/FVC long-term cross-sectional model, BMI main effect

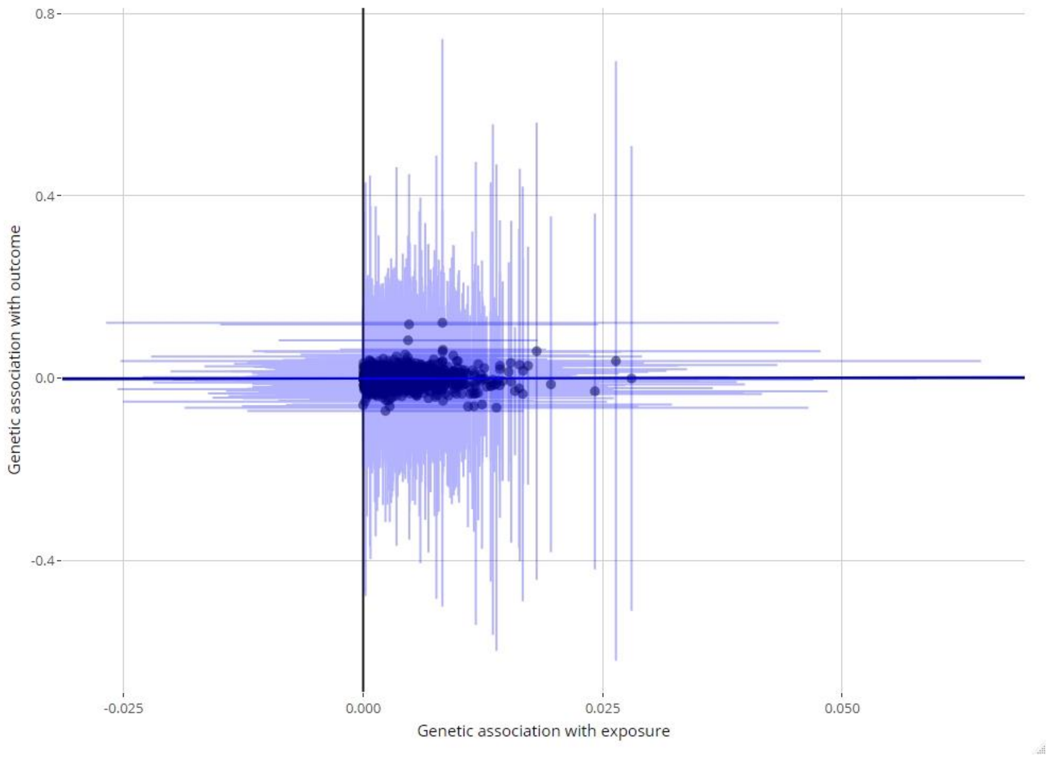

l) FEF2575/FVC long-term cross-sectional model, BMI:Age interaction effect

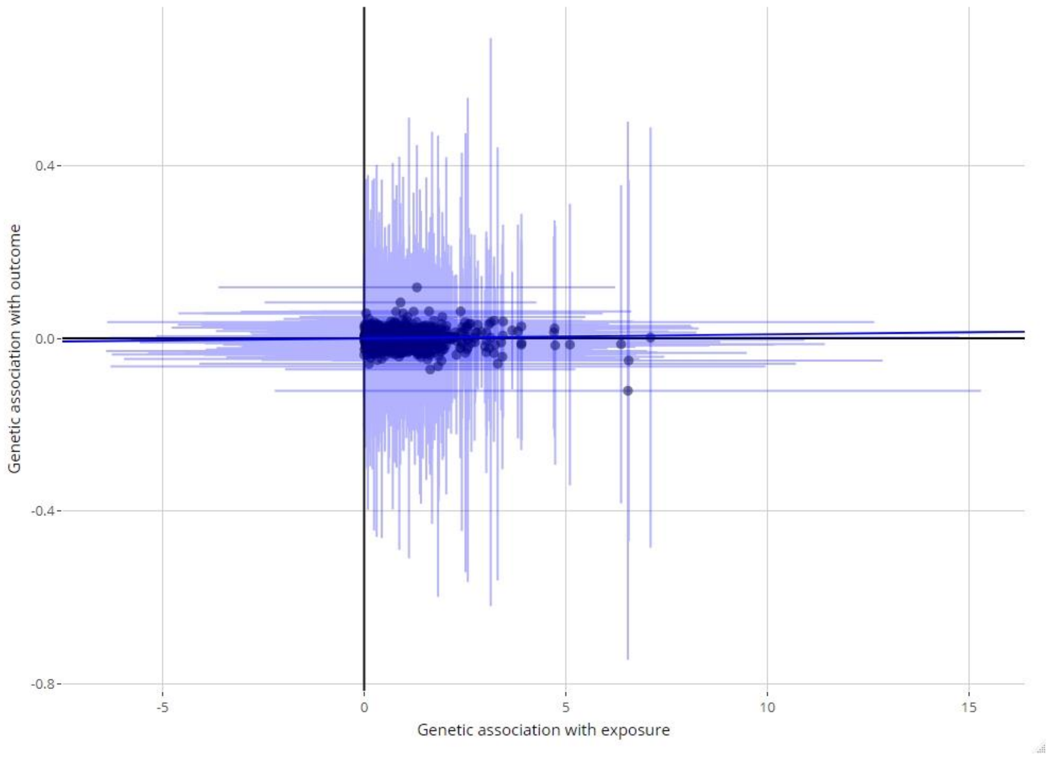

Supplement Figure 6. MR Egger regression results for Felix Score

a) FEV1/FVC predictive model, BMI main effect

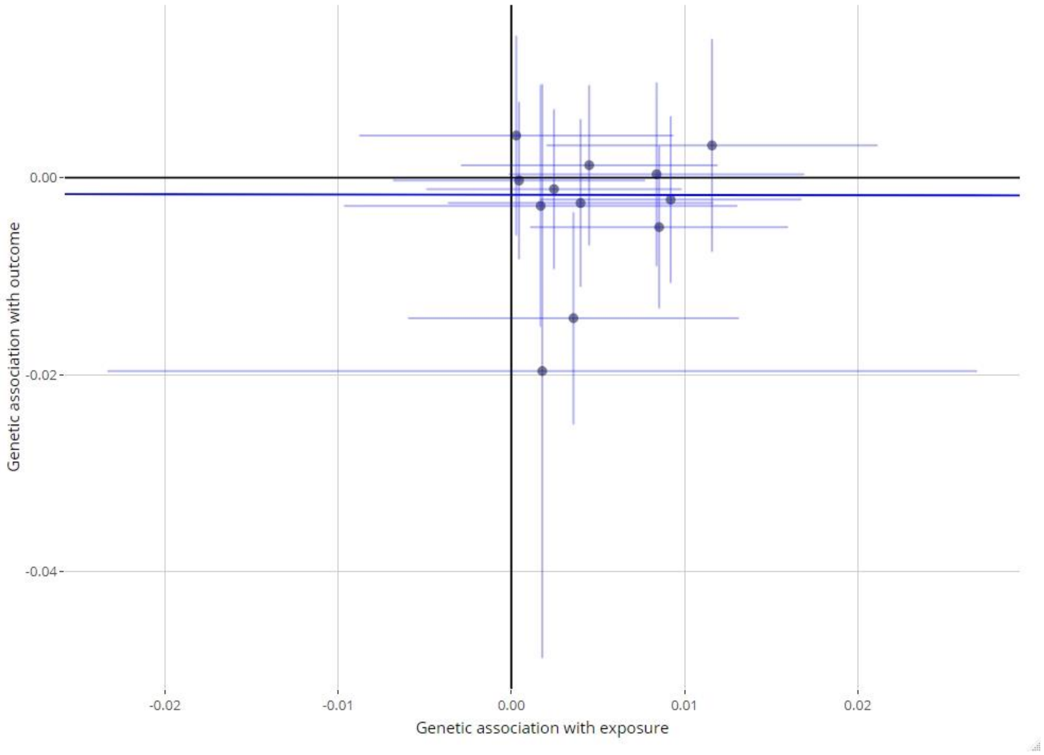

b) FEV/FVC predictive model, BMI:Age interaction effect

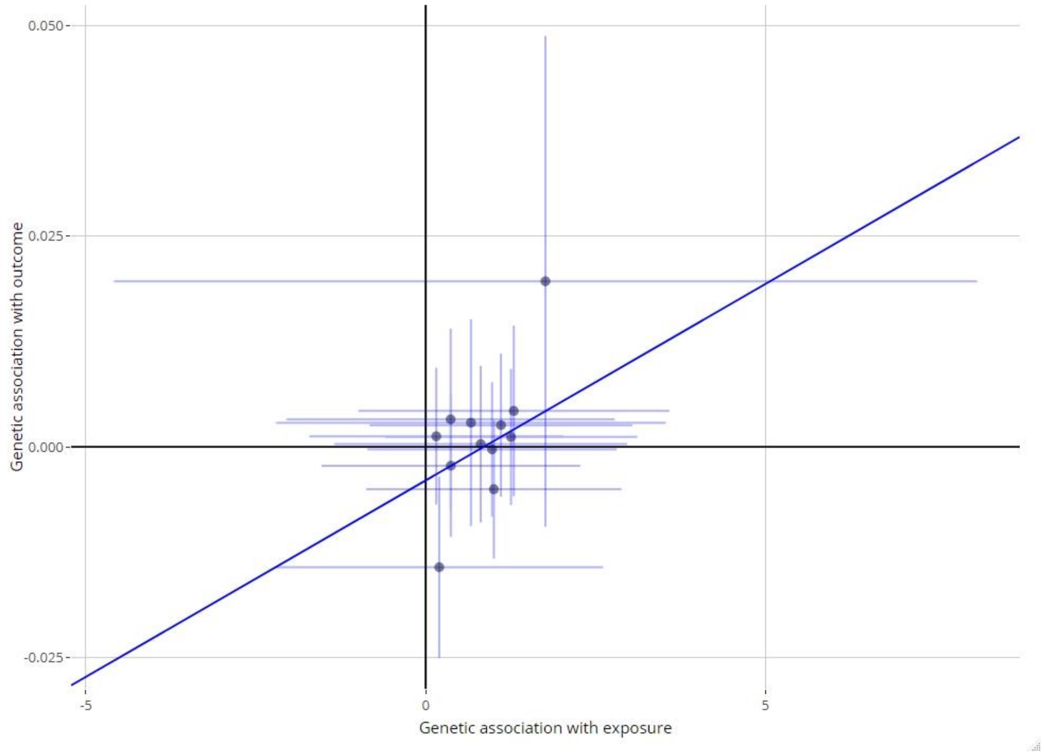

c) FEV1/FVC long-term cross-sectional model, BMI main effect

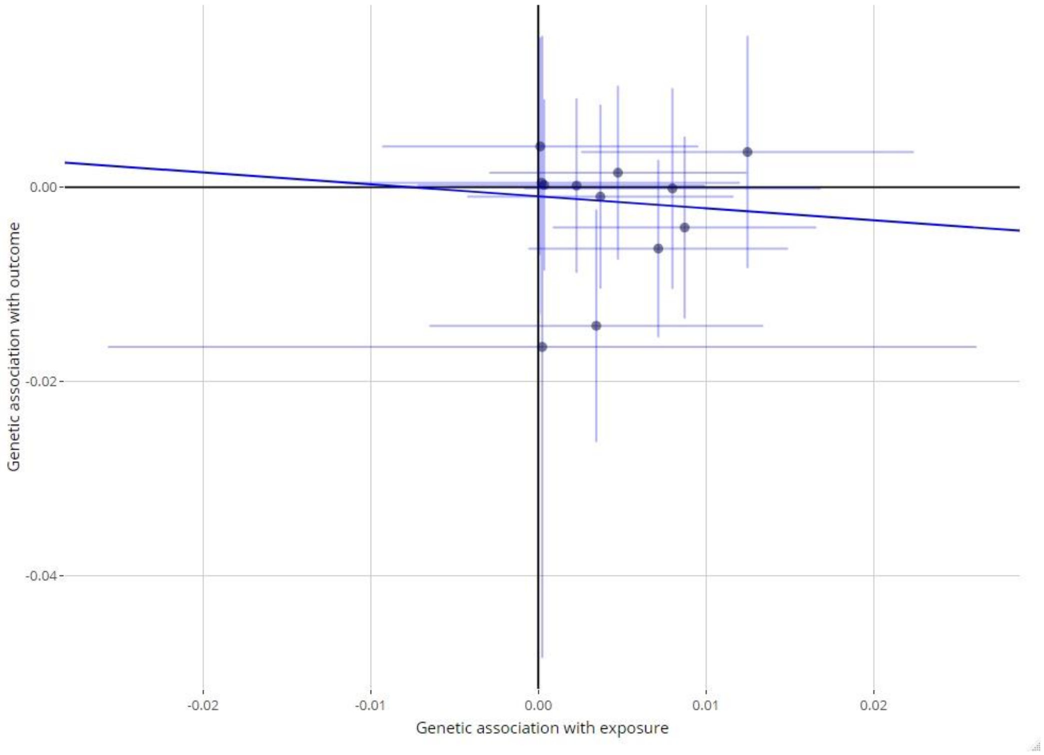

d) FEV/FVC long-term cross-sectional model, BMI:Age interaction effect

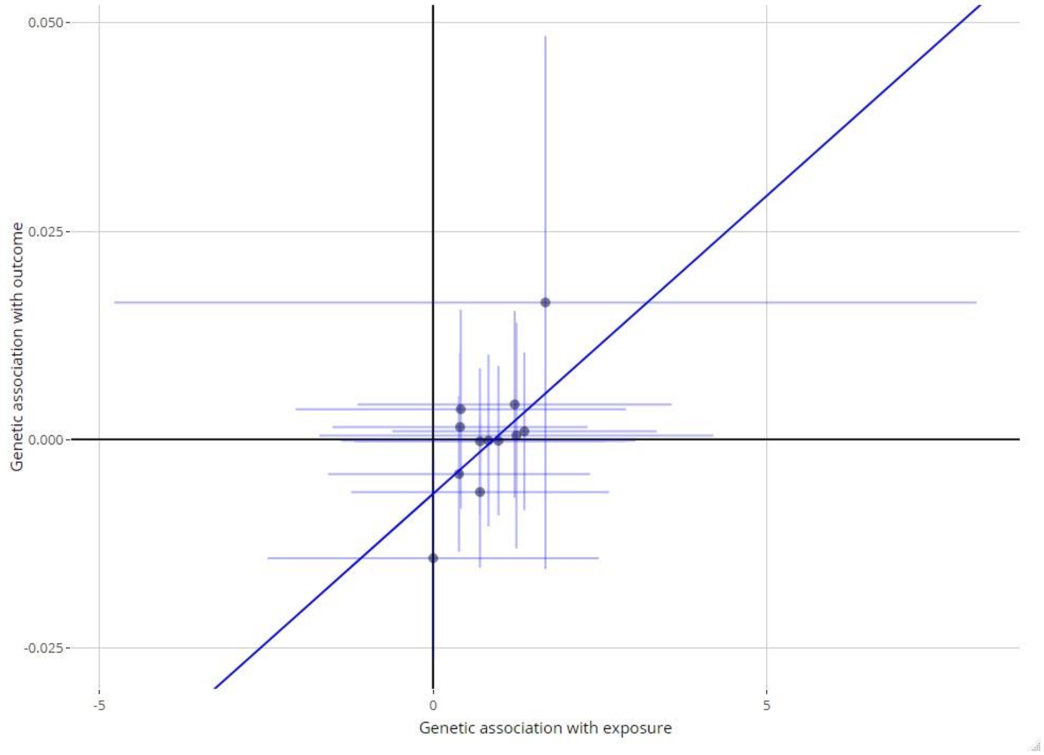

e) FEF2575 predictive model, BMI main effect

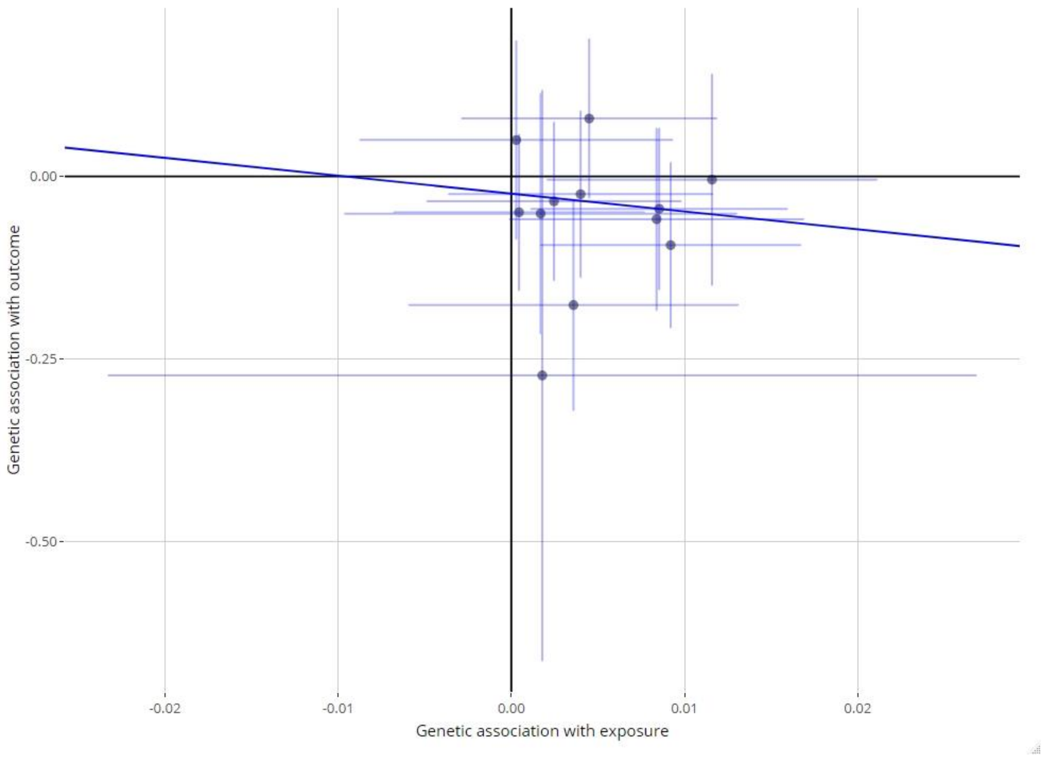

f) FEF2575 predictive model, BMI:Age interaction effect

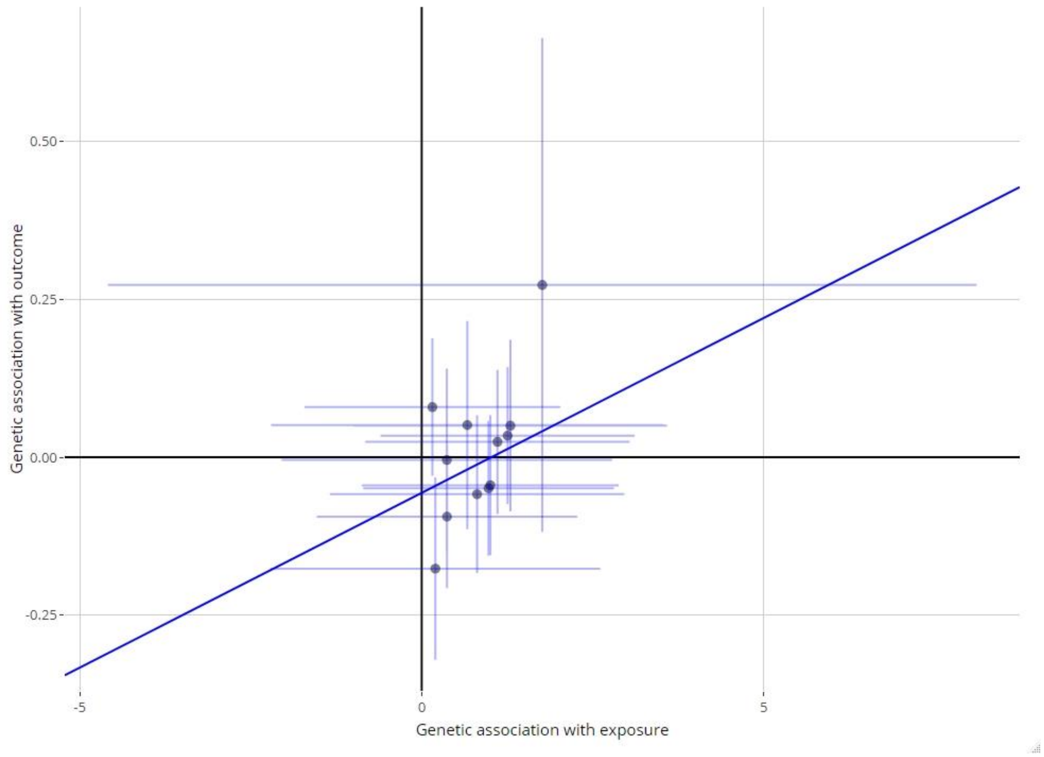

g) FEF2575 long-term cross-sectional model, BMI main effect

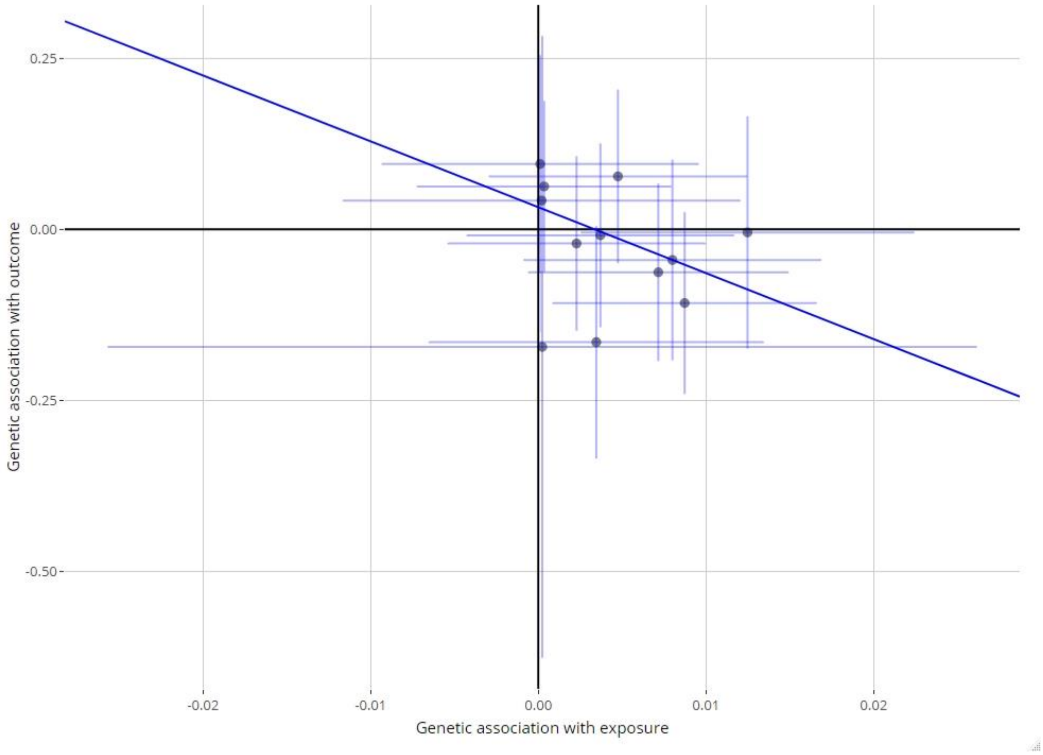

h) FEF2575 long-term cross-sectional model, BMI:Age interaction effect

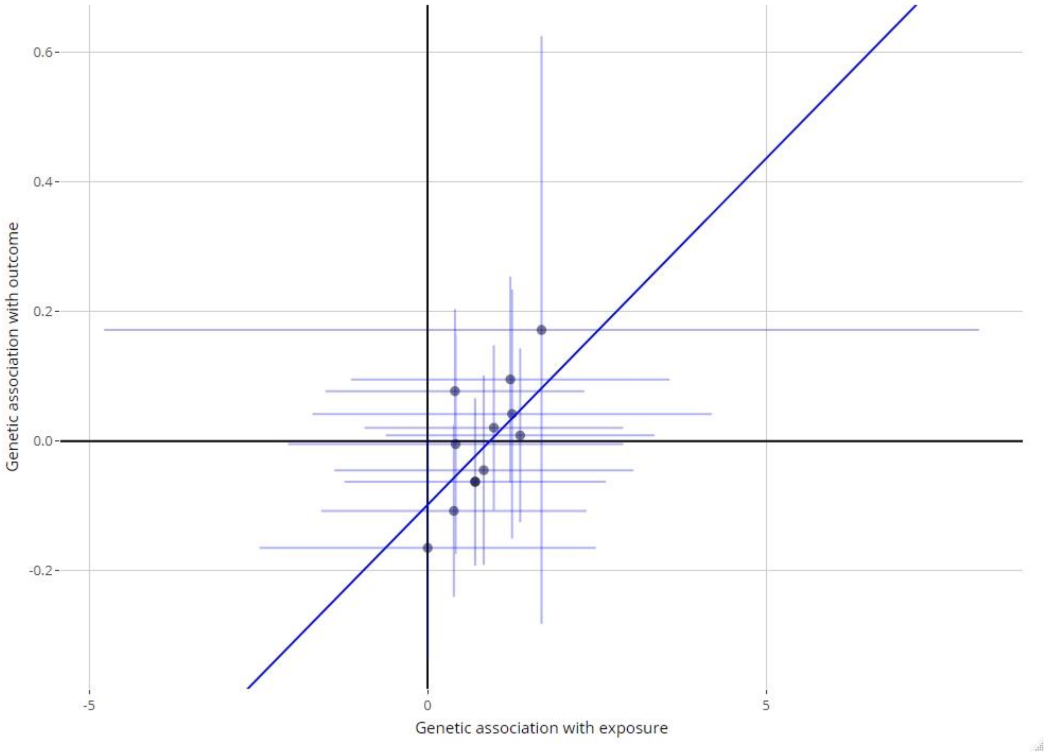

i) FEF2575/FVC predictive model, BMI main effect

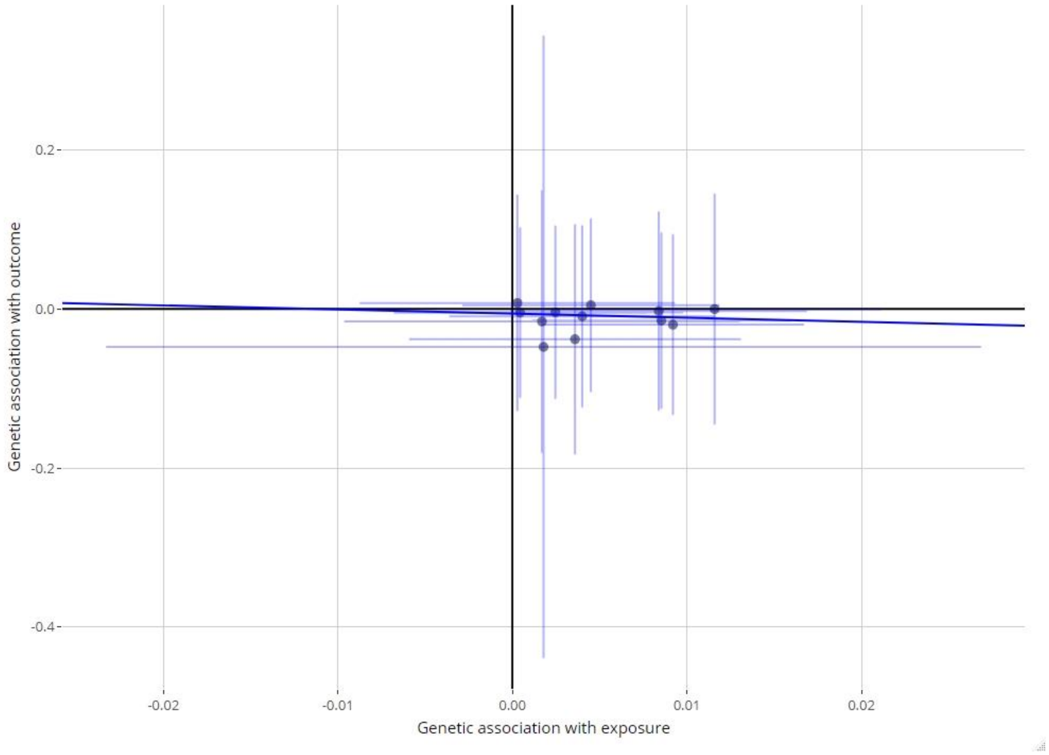

j) FEF2575/FVC predictive model, BMI:Age interaction effect

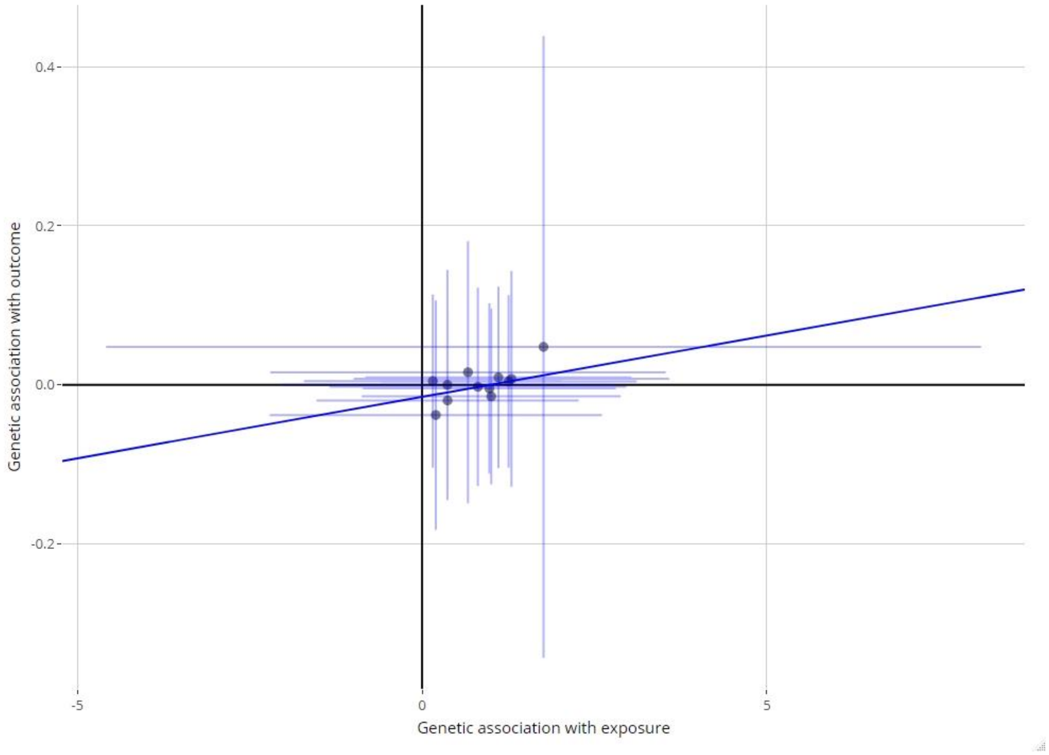

k) FEF2575/FVC long-term cross-sectional model, BMI main effect

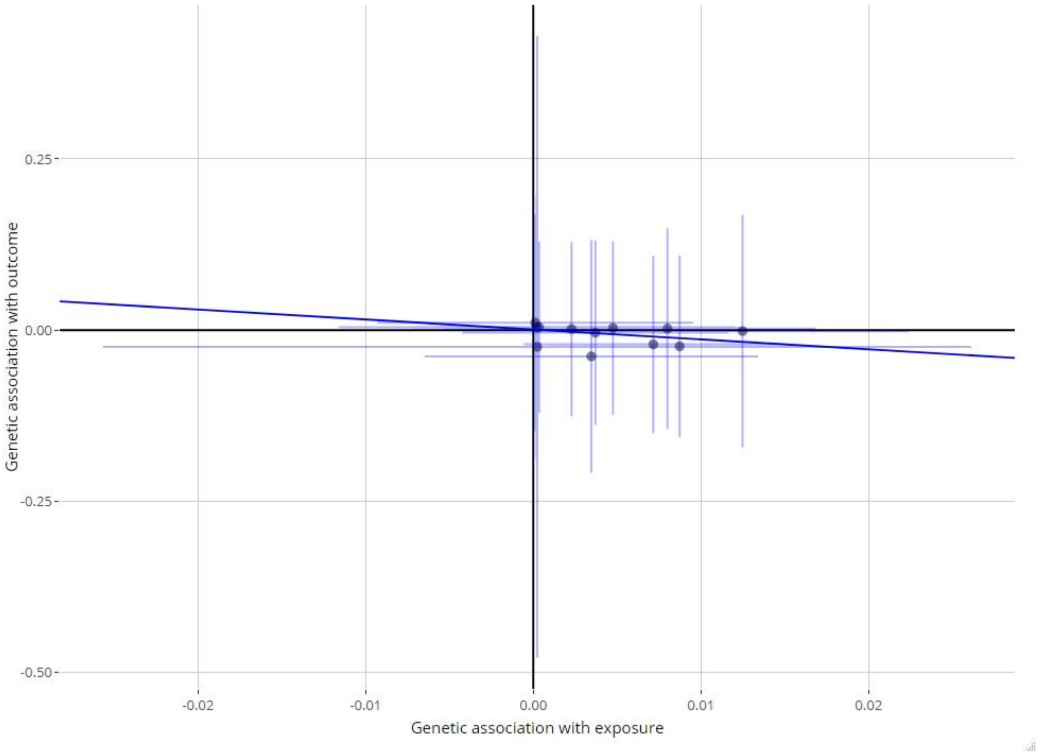

l) FEF2575/FVC long-term cross-sectional model, BMI:Age interaction effect

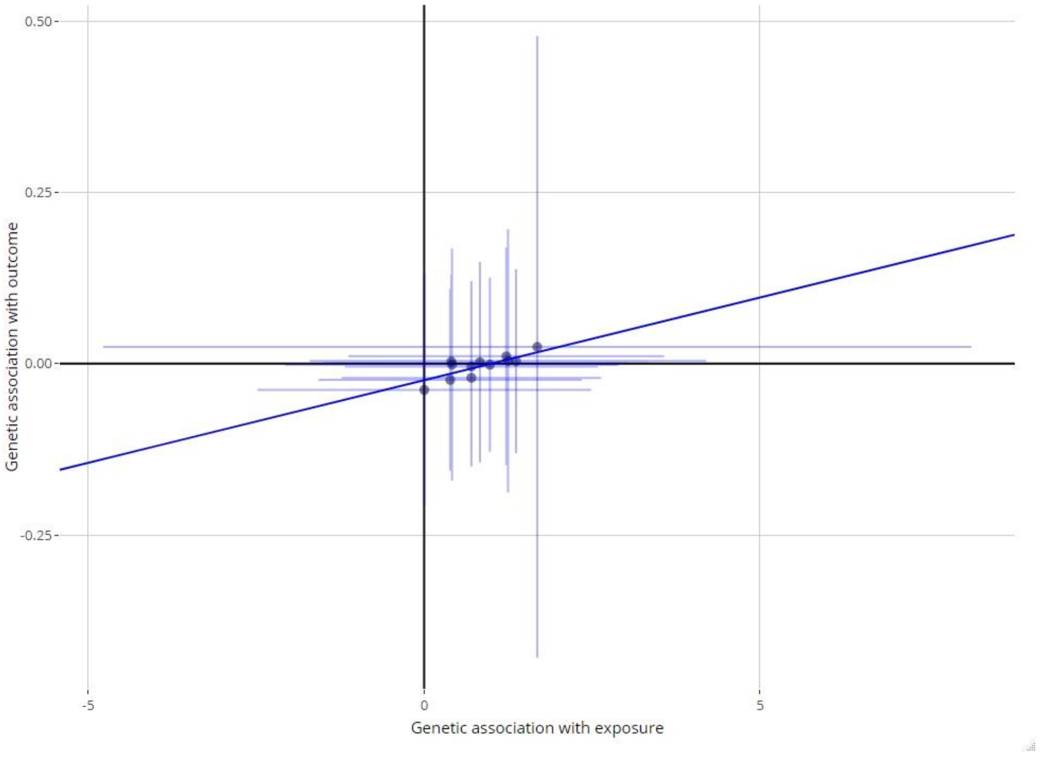

Supplement Figure 7. Histograms of packyears variables at SAP1, SAP2 and SAP3

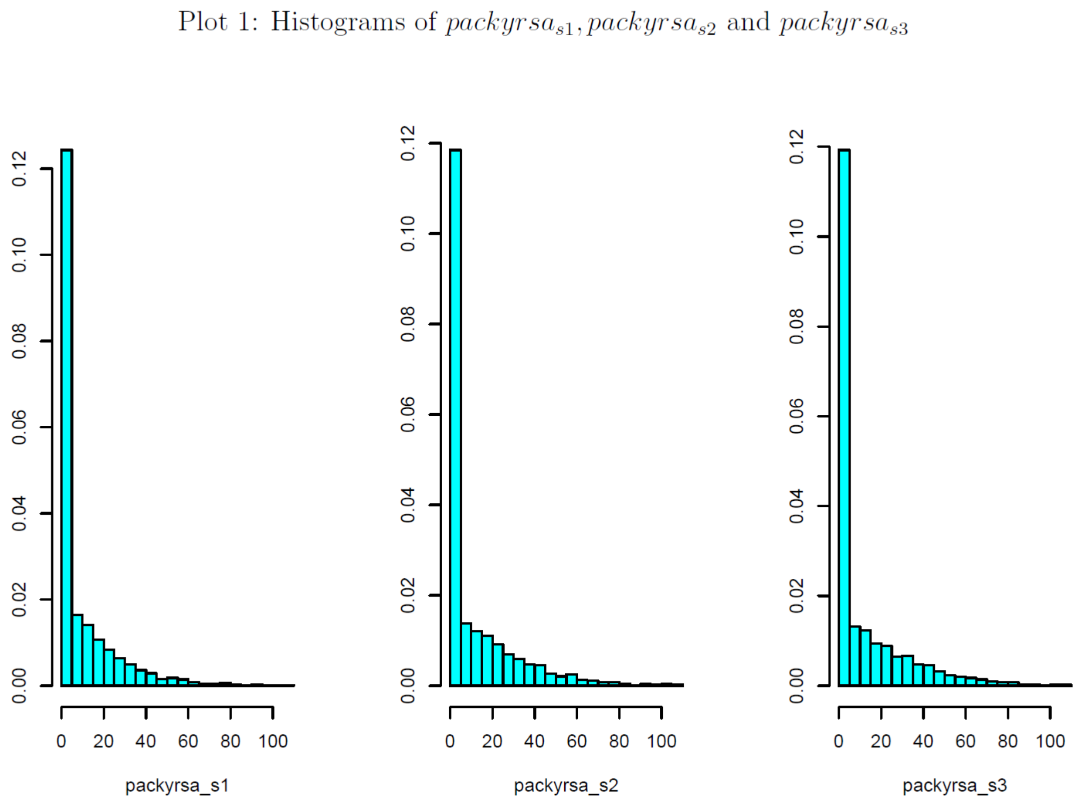

Acknowledgement:

Study directorate: NM Probst-Hensch (PI; e/g); C Schindler (s), N Künzli (e/exp), D Stolz (p)

Scientific team: JC Barthélémy (c), W Berger (g), R Bettschart (p), A Bircher (a), C Brombach (n), PO Bridevaux (p), L Burdet (p), Felber Dietrich D (e), T Sigrist (p), U Frey (pd), MW Gerbase (p), D Gold (e), E de Groot (c), W Karrer (p), F Kronenberg (g), B Martin (pa), A Mehta (e), D Miedinger (o), M Pons (p), F Roche (c), T Rothe (p), P Schmid-Grendelmeyer (a), D Stolz (p), A Schmidt-Trucksäss (pa), J Schwartz (e), A Turk (p), A von Eckardstein (cc), E Zemp Stutz (e).

Scientific team at coordinating centers: M Adam (e), I Aguilera (exp), A Beckmeyer-Borowko (e), S Brunner (s), D Carballo (c), S Caviezel (pa), I Curjuric (e), A Di Pascale (s), J Dratva (e), R Ducret (s), E Dupuis Lozeron (s), M Eeftens (exp), I Eze (e), E Fischer (g), M Foraster (e), M Germond (s), L Grize (s), S Hansen (e), A Hensel (s), M Imboden (g), A Ineichen (exp), A Jeong (g), D Keidel (s), A Kumar (g), N Maire (s), A Mehta (e), R Meier (exp), E Schaffner (s), T Schikowski (e), M Tsai (exp)

(a) allergology, (c) cardiology, (cc) clinical chemistry, (e) epidemiology, (exp) exposure, (g) genetic and molecular biology, (m) meteorology, (n) nutrition, (o) occupational health, (p) pneumology, (pa) physical activity, (pd) pediatrics, (s) statistics

Participants, technical and administrative support

The study could not have been done without the help of the study participants, technical and administrative support and the medical teams and field workers at the local study sites.

Local fieldworkers : Aarau: S Brun, G Giger, M Sperisen, M Stahel, Basel: C Bürli, C Dahler, N Oertli, I Harreh, F Karrer, G Novicic, N Wyttenbacher, Davos: A Saner, P Senn, R Winzeler, Geneva: F Bonfils, B Blicharz, C Landolt, J Rochat, Lugano: S Boccia, E Gehrig, MT Mandia, G Solari, B Viscardi, Montana: AP Bieri, C Darioly, M Maire, Payerne: F Ding,

P Danieli A Vonnez, Wald: D Bodmer, E Hochstrasser, R Kunz, C Meier, J Rakic, U Schafroth, A Walder.

Administrative staff epidemiological center: N Bauer Ott, C Gabriel

References

1. Miller MR, Hankinson J, Brusasco V, Burgos F, Casaburi R, Coates A, et al. Standardisation of spirometry. Eur Respir J. 2005;26(2):319-38.
2. Bridevaux PO, Dupuis-Lozeron E, Schindler C, Keidel D, Gerbase MW, Probst-Hensch NM, et al. Spirometer Replacement and Serial Lung Function Measurements in Population Studies: Results From the SAPALDIA Study. Am J Epidemiol. 2015;181(10):752-61.
3. Delaneau O, Marchini J, The Genomes Project C, McVean GA, Donnelly P, Lunter G, et al. Integrating sequence and array data to create an improved 1000 Genomes Project haplotype reference panel. Nature communications. 2014;5:3934.
4. Fuchsberger C, Abecasis GR, Hinds DA. minimac2: faster genotype imputation. Bioinformatics (Oxford, England). 2014;31(5):782-4.
5. Speliotes EK, Willer CJ, Berndt SI, Monda KL, Thorleifsson G, Jackson AU, et al. Association analyses of 249,796 individuals reveal 18 new loci associated with body mass index. Nat Genet. 2010;42(11):937-48.
6. Felix JF, Bradfield JP, Monnereau C, van der Valk RJ, Stergiakouli E, Chesi A, et al. Genome-wide association analysis identifies three new susceptibility loci for childhood body mass index. Hum Mol Genet. 2016;25(2):389-403.
7. Yengo L, Sidorenko J, Kemper KE, Zheng Z, Wood AR, Weedon MN, et al. Meta-analysis of genome-wide association studies for height and body mass index in approximately 700000 individuals of European ancestry. Hum Mol Genet. 2018;27(20):3641-9.
8. Millard LA, Davies NM, Timpson NJ, Tilling K, Flach PA, Davey Smith G. MR-PheWAS: hypothesis prioritization among potential causal effects of body mass index on many outcomes, using Mendelian randomization. Sci Rep. 2015;5:16645.
9. Locke AE, Kahali B, Berndt SI, Justice AE, Pers TH, Day FR, et al. Genetic studies of body mass index yield new insights for obesity biology. Nature. 2015;518(7538):197-206.
10. Didelez V, Sheehan N. Mendelian randomization as an instrumental variable approach to causal inference. Stat Methods Med Res. 2007;16(4):309-30.
11. Bun M, Harrison T. OLS and IV etimation of regression models including endogenous interaction terms. : LeBow College of Business, Drexel University; 2014.
12. VanderWeele TJ, Tchetgen Tchetgen EJ, Cornelis M, Kraft P. Methodological challenges in mendelian randomization. Epidemiology. 2014;25(3):427-35.
13. Abston E, Comellas A, Reed RM, Kim V, Wise RA, Brower R, et al. Higher BMI is associated with higher expiratory airflow normalised for lung volume (FEF25-75/FVC) in COPD. BMJ Open Respir Res. 2017;4(1):e000231.
14. Thorgeirsson TE, Gudbjartsson DF, Surakka I, Vink JM, Amin N, Geller F, et al. Sequence variants at CHRNB3-CHRNA6 and CYP2A6 affect smoking behavior. Nat Genet. 2010;42(5):448-53.
15. Tobacco, Genetics C. Genome-wide meta-analyses identify multiple loci associated with smoking behavior. Nat Genet. 2010;42(5):441-7.
16. Liu JZ, Tozzi F, Waterworth DM, Pillai SG, Muglia P, Middleton L, et al. Meta-analysis and imputation refines the association of 15q25 with smoking quantity. Nat Genet. 2010;42(5):436-40.
