# Supplementary material for: Causal Effects of Body Mass Index on Airflow Obstruction and Forced Mid-Expiratory Flow: A Mendelian Randomization Study Taking Interactions and Age-Specific Instruments Into Consideration Toward a Life Course Perspective
Source: Front Public Health. 2021 May 11;9:584955. doi: 10.3389/fpubh.2021.584955 (PMC8144328; doi:10.3389/fpubh.2021.584955)
Supplement: Supplementary file 2 [file Data_Sheet_2.docx]

#****************************************************************************

# *

# I. MENDELIAN RANDOMISATION ANALYSES using command ivreg() in library AER *

# *

# I.1 predictive causal model for FEV_1/FVC *

# I.2 predictive causal model for FEF2575 *

# I.3 long-term cross-sectional causal model for FEV_1/FVC *

# I.4 long-term cross-sectional causal model for FEF2575 *

# *

#****************************************************************************

library(AER)

#---------------------------------------------------------------------------#

# #

# predictive causal model: #

# SAP1-SAP2 averaged covariates -> SAP2-SAP3 averaged LungFunction #

# #

#---------------------------------------------------------------------------#

#

# compute additional explanatory variables for predictive model

#

BMI_logmean_s1s2 <- log(bmi_mean_s1s2)

age_mean_s1s2 <- (age_s1 + age_s2)/2

age_mean_s1s2_c18 <- age_mean_s1s2 - 18

height_mean_s1s2 <- (height_s1 + height_s2)/2

logBMIs1s2xAges1s2.prod <- BMI_logmean_s1s2*age_mean_s1s2_c18

BMIgsxAges1s2.prod <- score.32snps*age_mean_s1s2_c18

#------------------------------------------------------------------------------#

# #

# I.1 TiffR = FE1/FVC #

# #

#------------------------------------------------------------------------------#

#*******************************************************************************

# *

# IV estimated with 2SLS *

# using: i) FEV1/FVC_s3 corrected for new spirometry instrument *

# ii) mean(Age)_s1s2 and then centering it at 18 years *

# iii) mean(height)_s1s2, without centering *

# iv) Packyrsa_s2 as Smoking variable *

# v) Speliotes(2010) BMI genetic score (32 SNPs) *

# *

#*******************************************************************************

BMIgs_BMIlogmean_TiffRs2s3.MR <-

ivreg(TiffR_mean_corr_s2s3 ~ BMI_logmean_s1s2 + age_mean_s1s2_c18 +

sex_s2 + height_mean_s1s2 + packyrsa_s2 +

logBMIs1s2xAges1s2.prod + age_mean_s1s2_c18:sex_s2 +

age_mean_s1s2_c18:height_mean_s1s2 |

score.32snps + BMIgsxAges1s2.prod + age_mean_s1s2_c18 +

sex_s2 + height_mean_s1s2 + packyrsa_s2 +

I(age_mean_s1s2_c18*sex_s2) + I(age_mean_s1s2_c18*height_mean_s1s2))

summary(BMIgs_BMIlogmean_TiffRs2s3.MR)

#------------------------------------------------------------------------------#

# #

# I.2 FEF2575 #

# #

#------------------------------------------------------------------------------#

#******************************************************************************#

# #

# IV estimated with 2SLS #

# using: i) FEF2575_s3 corrected for new spirometry instrument #

# ii) mean(Age)_s1s2 and then centering it at 18 years #

# 111) mean(height)_s1s2, without centering #

# iv) Packyrsa_s2 as Smoking variable #

# v) Speliotes(2014) BMI genetic score #

# #

#******************************************************************************#

BMIgs_BMIlogmean_FEFs2s3.MR <-

ivreg(FEF_mean_corr_s2s3 ~ BMI_logmean_s1s2 + age_mean_s1s2_c18 +

sex_s2 + height_mean_s1s2 + packyrsa_s2 +

logBMIs1s2xAges1s2.prod + age_mean_s1s2_c18:sex_s2 +

age_mean_s1s2_c18:height_mean_s1s2 |

score.32snps + BMIgsxAges1s2.prod + age_mean_s1s2_c18 +

sex_s2 + height_mean_s1s2 + packyrsa_s2 +

I(age_mean_s1s2_c18*sex_s2) + I(age_mean_s1s2_c18*height_mean_s1s2))

summary(BMIgs_BMIlogmean_FEFs2s3.MR)

#------------------------------------------------------------------------------#

# #

# long-term cross-sectional causal model: #

# SAP1-SAP2-SAP3 averaged covariates -> SAP1-SAP2-SAP3 averaged LungFunction #

# #

#------------------------------------------------------------------------------#

#

# compute additional explanatory variables for long-term cross-sectional model

#

BMI_logmean <- log(bmi_mean)

age_mean <- (age_s1 + age_s2 + age_s3)/3

age_mean_c18 <- age_mean - 18

height_mean <- (height_s1 + height_s2 + height_s3)/3

BMIlogmeanxAgemean.prod <- BMI_logmean*age_mean_c18

BMIgsxAgemean.prod <- score.32snps*age_mean_c18

#------------------------------------------------------------------------------

#

# I.3 TiffR= FE= FE1/FVC 1/FVC

#

#------------------------------------------------------------------------------

#*******************************************************************************

# *

# IV estimated with 2SLS *

# using: i) FEV1/FVC_s3 corrected for new spirometry instrument *

# ii) mean(Age)_s1s2 and then centering it at 18 years *

# iii) mean(height)_s1s2, without centering *

# iv) Packyrsa_s3 as Smoking variable *

# v) Speliotes(2010) BMI genetic score (32 SNPs) *

# *

#*******************************************************************************

BMIgs_BMIlogmean_TiffRmean.MR <-

ivreg(TiffR_mean_corr ~ BMI_logmean + age_mean_c18 + sex_s2 + height_mean +

packyrsa_s3 + BMIlogmeanxAgemean.prod + age_mean_c18:sex_s2 +

age_mean_c18:height_mean|

score.32snps + BMIgsxAgemean.prod + age_mean_c18 +

sex_s2 + height_mean + packyrsa_s3 + I(age_mean_c18*sex_s2) +

I(age_mean_c18*height_mean))

summary(BMIgs_BMIlogmean_TiffRmean.MR)

#------------------------------------------------------------------------------#

# #

# I.4 FEF2575 #

# #

#------------------------------------------------------------------------------#

#******************************************************************************#

# #

# IV estimated with 2SLS #

# using: i) FEF2575_s3 corrected for new spirometry instrument #

# ii) mean(Age)_s1s2 and then centering it at 18 years #

# 111) mean(height)_s1s2, without centering #

# iv) Packyrsa_s3 as Smoking variable #

# v) Speliotes(2014) BMI genetic score #

# #

#******************************************************************************#

BMIgs_BMIlogmean_FEFmean.MR <-

ivreg(FEF_mean_corr ~ BMI_logmean + age_mean_c18 + sex_s2 + height_mean +

packyrsa_s3 + BMIlogmeanxAgemean.prod + age_mean_c18:sex_s2 +

age_mean_c18:height_mean|

score.32snps + BMIgsxAgemean.prod + age_mean_c18 +

sex_s2 + height_mean + packyrsa_s3 + I(age_mean_c18*sex_s2) +

I(age_mean_c18*height_mean))

summary(BMIgs_BMIlogmean_FEFmean.MR)
